# *Nmnat1* Deficiency Causes Mitoribosome Excess in Diabetic Nephropathy Mediated by Transcriptional Repressor HIC1

**DOI:** 10.3390/ijms25126384

**Published:** 2024-06-09

**Authors:** Kazuhiro Hasegawa, Masanori Tamaki, Yusuke Sakamaki, Shu Wakino

**Affiliations:** 1 Department of Nephrology, Tokushima University Graduate School of Biomedical Sciences, 3-18-15 Kuramoto-cho, Tokushima 770-8503, Japan; tamaki.masanori@tokushima-u.ac.jp (M.T.); shuwakino@tokushima-u.ac.jp (S.W.); 2 Department of Internal Medicine, Tokyo Dental College Ichikawa General Hospital, Chiba 272-8583, Japan; ysakamaki@tdc.ac.jp

**Keywords:** *Nmnat1*, mitoribosome, diabetic nephropathy

## Abstract

Nicotinamide adenine dinucleotide (NAD) is involved in renal physiology and is synthesized by nicotinamide mononucleotide adenylyltransferase (NMNAT). NMNAT exists as three isoforms, namely, NMNAT1, NMNAT2, and NMNAT3, encoded by *Nmnat1*, *Nmnat2*, and *Nmnat3*, respectively. In diabetic nephropathy (DN), NAD levels decrease, aggravating renal fibrosis. Conversely, sodium–glucose cotransporter-2 inhibitors increase NAD levels, mitigating renal fibrosis. In this regard, renal NAD synthesis has recently gained attention. However, the renal role of *Nmnat* in DN remains uncertain. Therefore, we investigated the role of *Nmnat* by establishing genetically engineered mice. Among the three isoforms, NMNAT1 levels were markedly reduced in the proximal tubules (PTs) of db/db mice. We examined the phenotypic changes in PT-specific *Nmnat1* conditional knockout (CKO) mice. In CKO mice, *Nmnat1* expression in PTs was downregulated when the tubules exhibited albuminuria, peritubular type IV collagen deposition, and mitochondrial ribosome (mitoribosome) excess. In CKO mice, *Nmnat1* deficiency-induced mitoribosome excess hindered mitoribosomal translation of mitochondrial inner membrane-associated oxidative phosphorylation complex I (CI), CIII, CIV, and CV proteins and mitoribosomal dysfunction. Furthermore, the expression of hypermethylated in cancer 1, a transcription repressor, was downregulated in CKO mice, causing mitoribosome excess. *Nmnat1* overexpression preserved mitoribosomal function, suggesting its protective role in DN.

## 1. Introduction

Diabetic nephropathy (DN) or diabetic kidney disease remains the leading background disease that worsens chronic kidney disease and progresses to end-stage renal disease [1]. The pathogenesis of DN has been reported previously. Sodium–glucose cotransporter-2 inhibitors and new nonsteroidal mineral corticoid blockers have been recently used to treat patients with DN in clinical settings [1]. However, these new treatments cannot completely delay or control DN progression. This may be because the DN-specific renal metabolism has not yet been elucidated.

We previously reported that type 1 diabetes mellitus (T1DM) induced by streptozotocin reduces the number of renal mitochondrial ribosomes (mitoribosomes), which is recovered by phosphoenolpyruvate carboxykinase 1 (*Pck1*) transgene (TG) [1]. Meanwhile, db/db-induced T2DM increases the number of renal mitoribosomes, which is not rescued by Pck1 TG [1]. Thus, some other molecular mechanism may be involved in the regulation of mitoribosomes.

The antibiotic doxycycline increases the level of nicotinamide adenine dinucleotide (NAD) and reduces the number of mitoribosomes, leading to organ protection and life prolongation through mild stress induced by the mitochondrial unfolded protein response (UPR^mt^) [2]. Thus, mitoribosome excess may result in organ injury and life shortening, which may be avoided by ensuring appropriate NAD levels.

The overexpression of nicotinamide mononucleotide adenylyltransferase (NMNAT), one of the NAD-biosynthetic enzymes, reportedly increases NAD levels [3]. NAD synthesis involves two pathways. NAD is predominantly synthesized through the salvage pathway, wherein sirtuin, nicotinamide phosphoribosyltransferase (NAMPT), and NMNAT catalyze the synthesis of NAD, nicotinamide mononucleotide (NMN), and nicotinamide (NAM) [4]. However, the ability of NMNAT to boost NAD levels and renal protection in vivo remains unclear. NMNAT has three isozymes: NMNAT1, NMNAT2, and NMNAT3. In our previous study, NMNAT1 levels were reduced in the kidneys of db/db mice but were significantly elevated after NMN treatment [5]. In addition, NMNAT1 is a potential target for several diseases [6]. We also previously demonstrated the important role of NAD salvage pathways in the proximal tubules (PTs) using PT-specific conditional knockout (CKO) and transgenic (TG) mice of SIRT1 [7] and NAMPT [8]. We proposed that changes in NAD metabolites affect glomerular phenotypes [9], and this interaction is known as tubuloglomerular interplay. Furthermore, NAMPT overexpression suppresses renal fibrosis, suggesting that renal tubular metabolism additionally affects renal fibrosis [8]. Furthermore, NMN administration helps maintain NAD levels and reverses diabetic podocyte injury [5]. Thus, NAD metabolism may play a significant role in renal fibrosis.

This study aimed to establish PT-specific *Nmnat1* CKO mice and investigate the pathogenic role of *Nmnat1* deficiency in DN.

## 2. Results

### 2.1. Changes in Nmnat1 Expression in db/db Mice

**Figure 1:**
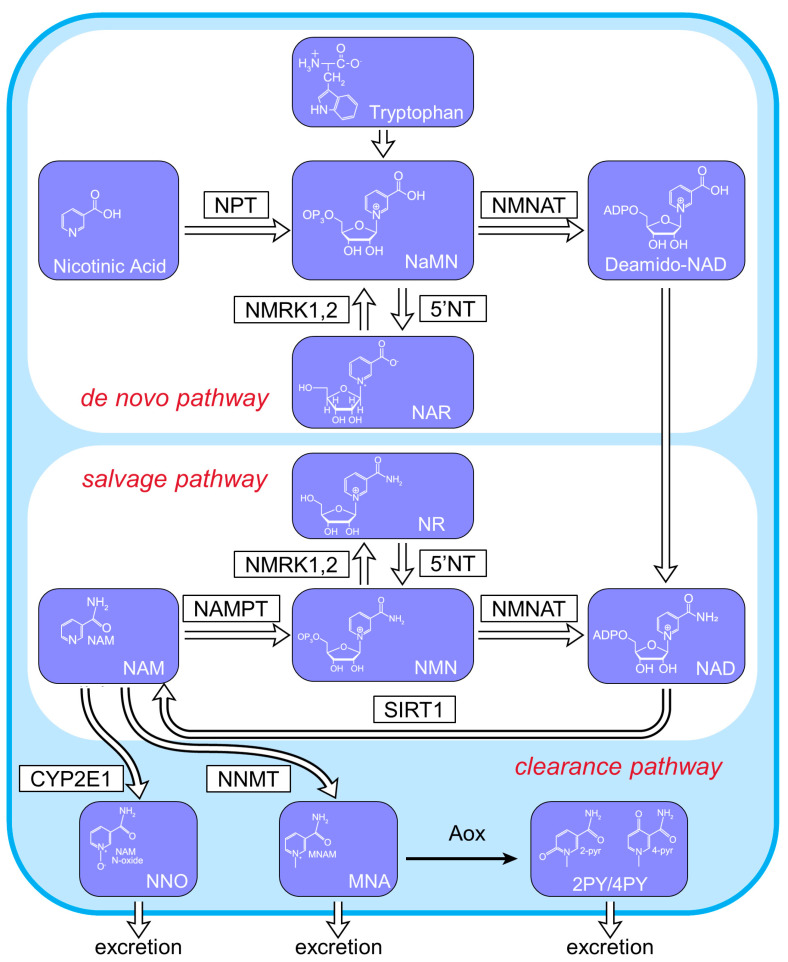
Schematic diagram showing the NAD^+^ metabolic pathway. NPT, nicotinic acid phosphoribosyltransferase; NMNAT, nicotinamide mononucleotide adenylyl transferase; NRK, nicotinamide riboside kinase; 5′-NT, 5′-nucleotidase; iNampt, intracellular NAM phosphoribosyl transferase; SIRT1, Sirtuin1; CYP2E1, cytochrome P450 2E1; NNMT, nicotinamide N-methyltransferase; AOX, aldehyde oxidase; NANM, nicotinic acid mononucleotide; NAR, nicotinic acid riboside; NAM, nicotinamide; NMN, nicotinamide mononucleotide; NR, nicotinamide riboside; NAD, nicotinamide adenine dinucleotide; NNO, NAM N-oxide; MNA, N1-methylniacinamide; 2PY, N1-methyl-2-pyridone-5-carboxamide; 4PY, N1-methyl-4-pyridone-3-carboxamide; PARP1, poly ADP-ribose polymerase 1.

**Figure 2:**
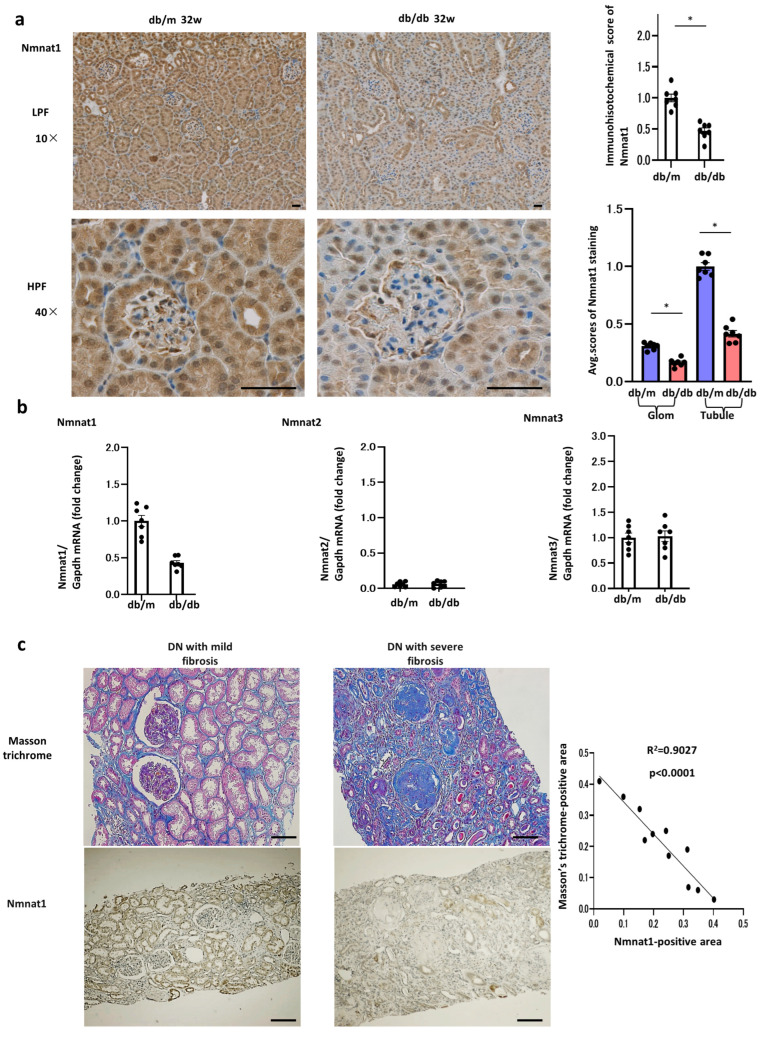
Reduced NMNAT1 levels in db/db mice and human renal fibrosis of DN. (**a**) Immunolocalization of NMNAT1 in the kidneys of nondiabetic control mice (db/m) and diabetic mice (db/db) at 32 weeks of age. The protein expression in the control and diabetic kidneys was examined through immunohistochemistry; representative images are shown. Positive protein expression was stained brown by a 3,3′-diaminobenzidine agent. Scale bar, 50 µm; N = 7. Results of immunohistochemical scoring assessing whole kidneys are expressed as the mean ± standard error of the mean (SEM). * *p* < 0.05. In addition, we have scored the Nmnat1 levels in tubular and glomerular areas separately. All data are depicted as the mean ± SEM. Horizontal bars denote statistically significant differences between the two groups. * *p* < 0.05. (**b**) Real-time quantitative reverse transcription–polymerase chain reaction (RT–PCR) analysis of the renal mRNA of NMNAT1, NMNAT2, and NMNAT3. The kidney tissue specimens for RT–PCR were derived from db/m and db/db mice at 32 weeks of age. N = 7 mice per group. Glyceraldehyde 3-phosphate dehydrogenase was used as a control. All data are depicted as the mean ± SEM. Horizontal bars denote statistically significant differences between the two groups. * *p* < 0.05. (**c**) Masson’s trichrome and NMNAT1 immunostaining in human kidneys. Representative photomicrographs of needle renal biopsy specimens from patients with DN with mild or severe fibrosis. The relationship between the Masson’s trichrome stain-positive area and the immunostaining intensity for NMNAT1 in renal biopsy specimens from patients with DN (N = 11). Pearson’s correlation analysis was used to calculate *r* and *p* values. Bars; 50 nm.

NMNAT converts NMN, an NAD^+^ precursor, into NAD in the salvage pathway (Figure 1). We assessed the expression of three isoforms of NMNAT, namely, NMNAT1, NMNAT2, and NMNAT3, in the kidneys of db/db mice and compared it with that in the kidneys of db/m mice at 32 weeks of age (Figure 2a,b). The expression of NMNAT1 was lower in db/db mice than in db/m mice, that of NMNAT3 remained unchanged, and that of NMNAT2 was undetectable in db/m mice. Therefore, we focused on the role of NMNAT1 in DN. Although some previous studies have described the nuclear localization of NMNAT1 in other organs [4], renal NMNAT1 was found in both the cytoplasm and nucleus. Consistent with this finding, the cytoplasmic localization [10] and function [11] of NMNAT1 have been described in other tissues. We also assessed the immunohistochemistry (IHCs) of NMNAT2 and 3. The immunostaining of NMNAT2 was absent in both the db/m and db/db mice. NMNAT3 was detectable in both murine groups, but no difference was noted between these groups (Supplementary Figure S1).

### 2.2. Nmnat1 Levels in Human Renal Biopsy Samples

We subsequently investigated whether *Nmnat1* has any relationship with human clinical parameters or tissue findings using renal biopsy samples and human data (Table 1). Based on the results of immunohistochemistry and Masson’s trichrome staining in kidney tissue specimens from 11 humans with DN, *Nmnat1* expression was inversely correlated with diabetic renal fibrosis in human kidneys (Table 1, Figure 2c). The kidneys of patients with severe renal fibrosis showed lower *Nmnat1* expression in PT regions and greater Masson’s trichrome staining than those of patients with milder fibrosis. Masson’s trichrome stain-positive areas were negatively correlated with *Nmnat1*-positive areas in PTs. Thus, the results of human renal biopsy suggest that reduced *Nmnat1* levels enhance diabetic fibrosis, consistent with the murine experimental results. Among several factors, only histological renal fibrosis based on Masson’s trichrome intensities negatively correlated with Nmnat1 immunostaining (Supplementary Figure S2). The clinical parameters eGFR, proteinuria, and serum creatine scores showed no correlations with the immunostaining levels for Nmnat1. 

### 2.3. Effects of Nmnat1 Deficiency in PTs on Proteinuria

**Figure 3:**
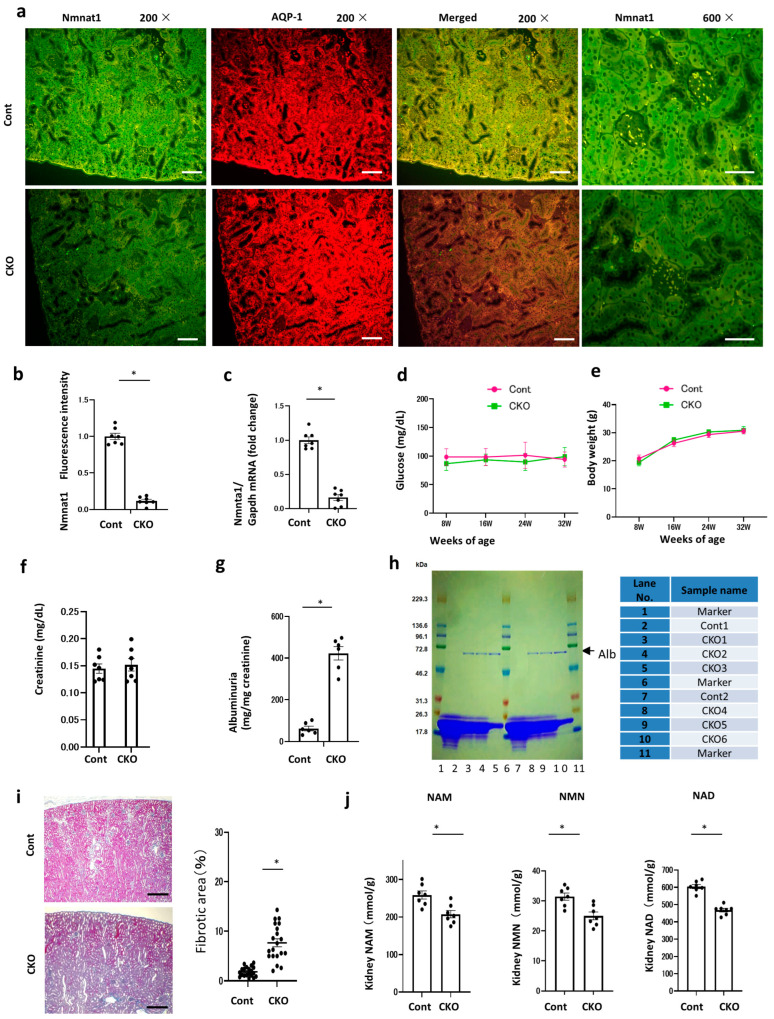
Effects of *Nmnat1* CKO on urinary albuminuria. (**a**) *Nmnat1* immunofluorescence intensity levels in each mouse group. Kidney tissue specimens obtained from each 32-week-old mouse were stained using immunofluorescence for *Nmnat1* (green) and AQP1 (red). (**b**) The panel shows the results of the quantitative analysis of *Nmnat1* fluorescence intensity. Scale bar, 50 µm; N = 7. (**c**) Real-time quantitative reverse transcription–PCR analysis of renal mRNA of *Nmnat1* (N = 7) in control and CKO mice at 32 weeks of age. (**d**) Temporal changes in the mean plasma glucose concentrations in mice from each group. We assessed the mice at 8, 16, 24, and 32 weeks of age. N = 7 mice per group. (**e**) Changes in mouse body weight from 8 to 32 weeks of age. N = 7 mice per group. Horizontal bars indicate statistically significant differences in each group. * *p* < 0.05. (**f**) Serum creatine levels in each mouse at 32 weeks of age. N = 6 mice per group. (**g**) Urinary albumin excretion in each mouse at 32 weeks of age. N = 6 mice per group. (**h**) Sodium dodecyl-sulfate–polyacrylamide gel electrophoresis (SDS–PAGE) of mouse urine samples. Urine samples of mice from each group at 32 weeks of age was tested using 15% SDS–PAGE before staining with Coomassie blue. N = 2 mice per group. AQP1, anti-aquaporin-1; *Nmnat1*, nicotinamide mononucleotide adenylyl transferase1; PCR, polymerase chain reaction; Cont, control; CKO, conditional knockout. (**i**) Representative histological findings of the kidneys of control and CKO mice via Masson’s trichrome staining. Scale bar = 500 nm. Percentage of renal fibrosis area (N = 20 mice per group). Horizontal bars indicate statistically significant differences in each group. * *p* < 0.05. (**j**) Renal tissue concentrations of NAD^+^ metabolites, NAM, NMN, and NAD at 32 weeks of age in the control and CKO groups (N = 7). Statistical significance between each group is represented by a horizontal bar. * *p* < 0.05.

To further evaluate the role of NMNAT1 in PTs, we established PT-specific *Nmnat1* CKO mice (Figure 3a). Based on the results of immunofluorescence staining, 32-week-old *Nmnat1* CKO mice demonstrated reduced NMNAT1 expression compared with the control mice (Figure 3b). Reverse transcription–polymerase chain reaction (RT–PCR) and Western blot analysis for *Nmnat1* revealed similar results (Figure 3c, Supplementary Figure S3b). Furthermore, serum glucose levels did not differ between control and CKO mice at 8, 16, 24, and 32 weeks of age (Figure 3d). Body weights were not significantly different between the two groups at 8, 16, 24, and 32 weeks of age (Figure 3e). Although *Nmnat1* deficiency-mediated NAD reduction may affect glucose metabolism and body weights, the serum glucose concentrations and body weights of mice remained unchanged. Moreover, the serum creatinine concentrations were identical in the two groups (Figure 3f), but urinary albumin excretion was significantly higher in CKO mice than in control mice (Figure 3g). To verify this finding, we analyzed urinary protein excretion via sodium dodecyl-sulfate–polyacrylamide gel electrophoresis (SDS–PAGE), which revealed the presence of albuminuria in CKO mice (Figure 3h). Masson’s trichrome staining demonstrated renal fibrotic changes in CKO mice (Figure 3i). These results were consistent with the renal fibrotic changes observed in patients with DN when *Nmnat1* expression was decreased in PTs (Figure 2c). To evaluate the changes in NAD metabolites, we examined kidney NAM, NMN, and NAD concentrations in CKO and control mice at 32 weeks of age. Compared with the control mice, CKO mice had lower NAM, NMN, and NAD^+^ concentrations in the kidneys at 32 weeks of age (Figure 3j). 

### 2.4. Effects of Nmnat1 Deficiency in PTs on Albuminuria

**Figure 4:**
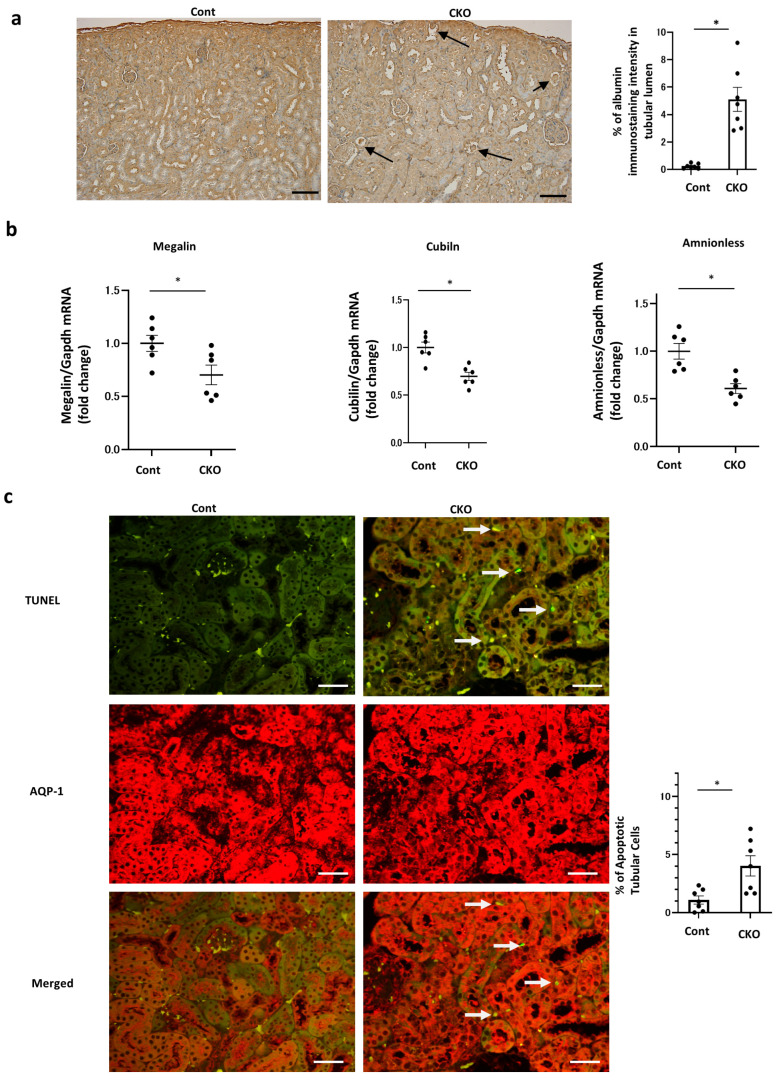
Renal phenotypes in *Nmnat1* CKO mice. (**a**) Representative images of albumin staining in each mouse. Arrows denote albumin cast in CKO mice. The right panel shows the relative staining intensity. N = 7 mice per group. (**b**) Changes in albumin uptake-related molecules in *Pck1* CKO mice. The kidney tissue specimens for RT–PCR were derived from CKO and control mice at 32 weeks of age. N = 7 mice per group. Real-time quantitative reverse transcription–polymerase chain reaction (RT–PCR) analysis of renal mRNA of megalin, cubilin, and amnionless. Glyceraldehyde 3-phosphate dehydrogenase was used as a control. All data are depicted as the mean ± standard error of the mean. Horizontal bars denote statistically significant differences between the two groups. * *p* < 0.05. (**c**) Representative immunofluorescence double staining of TUNEL (green) and AQP1 (red, proximal tubules) in kidney tissues (N = 7). Scale bars, 50 µm. The kidney tissue specimens were derived from Cont and CKO mice at 32 weeks of age. White arrows denote apoptotic tubular cells.

To investigate the underlying mechanisms of albuminuria and detect albumin reabsorption, we conducted albumin staining and found that its intensity in the intracellular tubular area, which demonstrates albumin reabsorption, was lower in CKO mice than in control mice. Additionally, CKO mice demonstrated intratubular albumin casts, which indicated fewer albumin reuptakes (Figure 4a). Consistent with this finding, RT–PCR assays and Western blot analysis revealed that the levels of albumin reuptake molecules such as megalin, cubilin, and amnioless in PTs were reduced in CKO mice (Figure 4b, Supplementary Figure S4), which could result in albumin casts in tubular lumens and concomitant albuminuria. Furthermore, the proportion of apoptotic tubular cells was higher in CKO mice than in control mice, as shown via TUNEL staining (Figure 4c). These data suggested that CKO mice exhibited tubular damage that reduced albumin reabsorption and caused a concomitant increase in albuminuria. 

### 2.5. Effects of Nmnat1 Deficiency in PTs on Renal Fibrotic Changes

**Figure 5:**
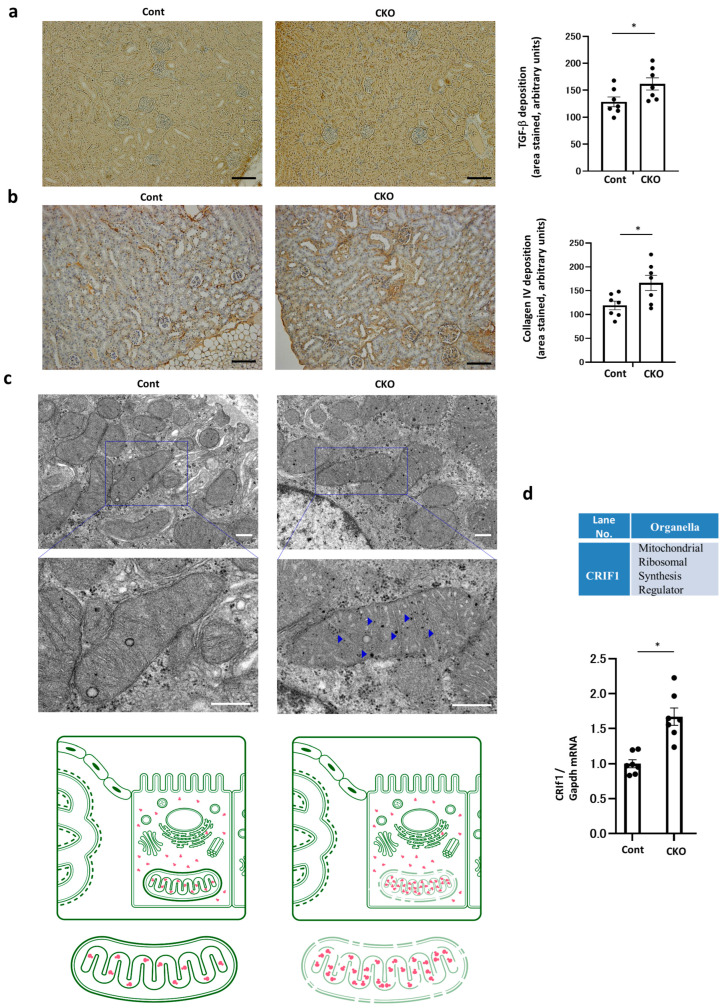

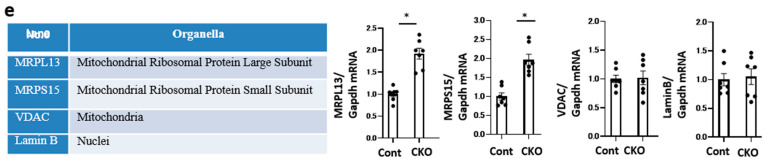
Mitoribosome excess in *Nmnat1* CKO mice. (**a**) Representative photomicrographs showing TGF-β immunostaining in each group. The bar graph represents the quantitative analysis of TGF-β staining. N = 7 mice per group. Scale bar = 100 μm. (**b**) Representative photomicrographs indicate collagen IV immunostaining in each group. The bar graph shows the quantitative analysis of collagen IV-stained areas. N = 7 mice per group. Scale bar = 100 μm. Kidney tissue specimens for immunostaining were derived from the four 32-week-old mouse groups. All data are depicted as mean ± standard error of the mean. Horizontal bars indicate statistically significant differences in each group. * *p* < 0.05. (**c**) Representative electron micrograph in each group. Scale bar = 500 nm. Blue squares indicate the enlarged regions. Expanded images are also presented. Blue arrowheads indicate mitoribosomes. Scale bar = 500 nm. Illustration depicts mitoribosome excess in CKO mice. The number of mitoribosomes was intact in Cont mice. For electron microscopy, the kidney tissue specimens were embedded into Epon epoxy resin. Electron micrographs of 10 proximal tubules (PTs) per kidney were randomly obtained for each mouse to evaluate the morphometry of PTs. The red symbols represent mitoribosomes. (**d**) Real-time quantitative reverse transcription–polymerase chain reaction (RT–PCR) analysis of the renal mRNA of CRIF1, a mitoribosomal synthesis regulator. Glyceraldehyde 3-phosphate dehydrogenase was used as a control. The kidney tissue specimens for RT–PCR were obtained from CKO and control mice at 32 weeks of age. N = 7 mice per group. (**e**) Real-time quantitative reverse transcription–polymerase chain reaction (RT–PCR) analysis of the renal mRNA of intracellular organelle markers. MRPL13 and MRPS15 are mitoribosomal proteins, VDAC is a mitochondrial protein, and Lamin B is a nuclear protein. Glyceraldehyde 3-phosphate dehydrogenase was used as a control. For RT–PCR, the kidney tissue specimens were obtained from CKO and control mice at 32 weeks of age. N = 7 mice per group.

To explore whether the levels of main renal profibrotic factors, such as TGF-β and type IV collagen, were elevated in CKO mice, similar to the results of Masson’s trichrome staining, an immunostaining assay and RT–PCR assays were performed. CKO mice demonstrated higher levels of TGF-β and deposition of type IV collagen, resulting in renal fibrotic changes (Figure 5a,b, Supplementary Figure S5). CKO leads to renal peritubular fibrotic changes even under nondiabetic conditions, indicating that proximal tubular *Nmnat1* deficiency has profibrotic effects on the kidney. In addition, the fibronectin and α-SMA protein levels were also higher in CKO mice than in Cont mice, indicating the fibrotic changes in CKO mice; this result is consistent with that of TGF- and type IV collagen depositions (Supplementary Figure S6). 

Previous reports have shown that mitoribosome defects cause the upregulation of TGF-β expression [12]. Thus, we examined the electron microscopy (EM) findings to further elucidate whether morphological or functional changes in intracellular organelles were associated with such profibrotic phenotypes, by specifically focusing on the observation of mitoribosomes. The EM images of the mitochondria clearly revealed that the tiny black (electron dense) spots inside the mitochondria were more abundant in CKO mice than in control mice (Figure 5c). These spots appeared as praying beads, corresponding to mitoribosomes (Figure 5c). Thus, mitoribosome excess in CKO mice showed an increased density of tiny black spots. In the cytoplasm, the number of dense black spots was not altered in CKO mice compared with that of control mice. These cytoplasmic spots indicate the ribosomes present in the cytoplasm (cytoribosomes). In the expanded images of EM, the arrowheads indicate mitoribosomes, which were observed previously [1]. In addition, Supplementary Figure S7 shows a much higher magnification of mitoribosomes in CKO and Cont mice (Supplementary Figure S7). Our novel findings suggest that *Nmnat1* deficiency increases the number of mitoribosomes in the kidneys (Figure 5c). The proliferation of ribosomes is mediated by CRIF1, a key regulator of mitoribosomes [1]. Therefore, we assessed the CRIF1 levels in CKO and control mice and found that CRIF1 was greatly augmented in CKO mice (Figure 5d, Supplementary Figure S8). Thus, increased CRIF1 levels induced mitoribosome excess in CKO mice. To quantitatively assess the number of mitoribosomes in CKO mice, we monitored MRPs as mitoribosmal markers and compared them with other organelle marker proteins. MRPS15 and MRPL13 are mitoribosomal markers, whereas voltage-dependent anion channel (VDAC) and LaminB are mitochondrial and nuclear markers. The levels of MRPs, such as MRPS15 and MRPL13, increased in CKO mice (Figure 5e). However, VDAC and LaminB levels remained unchanged in both CKO and control mice. Additionally, considering that mitoribosomal dysfunction reportedly promotes the deposition of type IV collagen (the main component of renal fibrosis) [1], we next investigated whether increased mitoribosomes cause mitoribosomal dysfunction.

### 2.6. Regulatory Mechanism of Nmnat1 Deficiency-Induced CRIF1 Upregulation

**Figure 6:**
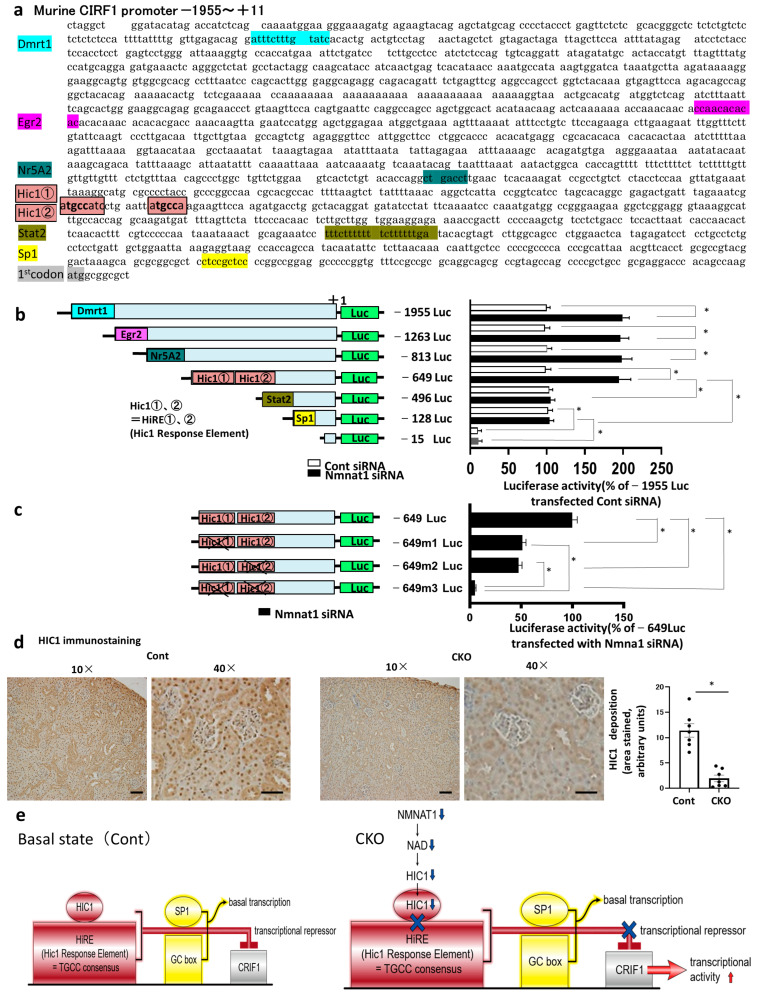
CRIF1 gene regulation by HIC1 and SP1. (**a**) Localization and nucleotide sequence in the murine CRIF1 promoter region. The red characters represent the effects of *Nmnat1* deficiency–mediated by two putative HIC1-responsive element (HiREs) on CRIF1 upregulation. The transcription start sites are indicated in gray. (**b**) The schematic diagram describes seven deletion mutants in the *CRIF1* promoter sequences (−1955, −1263, −813, −649, −496, −128, and −15) that were cloned upstream from a luciferase reporter gene. The bar graphs present the results of transient transfection of cultured proximal tubules (PTs), illustrating the promoter activities associated with each deletion. Luciferase activity is shown relative to that of the −1955 Luc vector in the control vector-transfected cells. Values are expressed as the mean ± standard error of the mean (SEM). * *p* < 0.05 vs. each Luc transfected PTs (N = 3 independent experiments). (**c**) Mutation analysis of *CRIF1* promoter activity in cultured PT cells. −649 Luc, WT *Nmnat1* promoter; M1, proximal HiRE mutation; M2, distal HiRE mutation; M3, mutation in both HiREs corresponding to the HIC1 binding sites. * *p* < 0.05 vs. −649 Luc in control cells (N = 3 independent experiments). (**d**) Representative photomicrographs showing HIC1 immunostaining in each group. The bar graph illustrates the results of quantitative analysis. N = 7 mice per group. Light micrograph; scale bar = 100 μm. Kidney tissue specimens for immunostaining were obtained from four mouse groups at 32 weeks of age. All data are shown as mean ± SEM. Horizontal bars indicate statistically significant differences in each group. * *p* < 0.05. (**e**) Schematic representation of the murine *CRIF1* gene and promoter. The solid boxes indicate the binding of HiRE to HIC1 and binding of GC box to Sp1, highlighted in red and yellow, respectively. Cont, control; CKO, conditional knockout; CRIF1, CR6-interacting factor 1; Dmrt1, double sex and mab-3 related transcription factor 1; Egr2, early growth response protein 2; Nr5A2, nuclear receptor subfamily 5 group A member 2; HIC1, HIC ZBTB transcriptional repressor 1; STAT2, signal transducer and activator of transcription 2; Sp1, specificity protein 1; Luc, luciferase; *Nmnat*, nicotinamide mononucleotide adenylyl transferase; DN, diabetic nephropathy.

We analyzed the regulatory mechanisms of *Nmnat1* deficiency-induced CRIF1 upregulation by assessing the 5′-flanking region (2 kb) of murine CRIF1 gene (Figure 6a). To initially locate the functional regions that regulate *CRIF1* expression in cultured PTs, we used several 5′-deletion constructs, with luciferase as the reporter gene, for transient transfection studies (Figure 6b). Sequence analysis using TRANSFAC software 7.0 revealed the localization of putative transcription factor binding sites for DMRT1, EGR2, NR5A2, HIC1, STAT2, and SP1 within a 1966-bp region in the *Nmnat1* promoter (−1955 to +1) surrounding the major transcriptional start site (Figure 6a,b). We identified two HIC1 binding sites (HIC1-responsive element [HiRE]) in the promoter region (TGCC consensus) and detected the Sp1-binding site (GC box). Luciferase assays were conducted in cultured PTs to measure the *Nmnat1* siRNA-treated promoter activities of seven deletion constructs (−1955 Luc, −1263 Luc, −813 Luc, −649 Luc, −496 Luc, −128 Luc, and −15 Luc) that were cloned upstream from luciferase reporter genes. In the −1955 Luc, −1263 Luc, −813 Luc, and −649 Luc deletion constructs, the transcriptional activities of CRIF1 promoters were not affected. Following *Nmnat1* siRNA transfection, similar upregulation levels were observed in cells containing these four promoter constructs. Nevertheless, the activity of the CRIF1 promoter was markedly suppressed in cells transfected with the promoter that deleted the region from −649 to −496 (−496 Luc) following *Nmnat1* siRNA treatment, demonstrating similar promoter activities with or without *Nmnat1* siRNA transfection. Thus, the promoter region spanning from −649 to −496 is essential for upregulating *Nmnat1* deficiency-induced CRIF1 expression. Furthermore, CRIF1 promoter activity was distinctly depressed in cells transfected with the promoter that deleted the region from −128 to −15 (−15 Luc), demonstrating similarly low activities with and without *Nmnat1* siRNA transfection. Therefore, the promoter region spanning from −128 to −15 may be crucial for basal CRIF1 transcription. TRANSFAC analysis showed that this *Nmnat1* deficiency response region (between −649 and −496) contained two consensus sites for HIC1 binding (Figure 6c), corresponding to the HiREs containing TGCC consensus sites. Luciferase assays conducted with the mutated HiRE sites (one or both) showed that both sites were functional (Figure 6c). Our results also indicated that Sp1 was mainly involved in the basic transcription of *CRIF1* transcription. Consistent with this notion, Sp1 binding sites involving GC box were located in the promoter region spanning from −125 to −15. Regarding HIC1 expression changes, the intensity of HIC1 immunostaining and RT-PCR analysis significantly decreased in CKO mice compared with that of control mice (Figure 6d, Supplementary Figure S9). HIC1, a key transcriptional repressor, suppressed the downstream target genes. In CKO mice, *Nmnat1* deficiency decreased HIC1 expression and increased CRIF1 expression (Figure 6e, right panel). Meanwhile, basal CRIF1 transcription was maintained by Sp1, as observed in the control mice (Figure 6e, left panel).

To determine whether CIRF1 promoter activity is dispensable for Hic1 activity and its binding activity to HiRE, we constructed a Hic1 vector and transfected it into cultured proximal tubular cells. The overexpression of Hic1 significantly reduced CRIF1 promoter activity. In addition, by introducing a mutation in the HiRE sites, the HIC1-induced transcription activation of the CRIF1 promoter ceased (Supplementary Figure S10). Next, we assessed and confirmed the regulatory mechanisms that control CRIF1 expression via Nmnat1/HIC1. Treatment with a specific siRNA for Nmnat1 suppressed HIC1 expression and upregulated CRIF1 expression (Supplementary Figure S11). Thus, reduced Nmnat1 upregulated CRIF1 expression by downregulating HIC1. 

### 2.7. Mitoribosome Excess and CRIF1 Downregulation Are Responsible for Diabetic Peritubular Fibrotic Changes via Nmnat1 Deficiency

**Figure 7:**
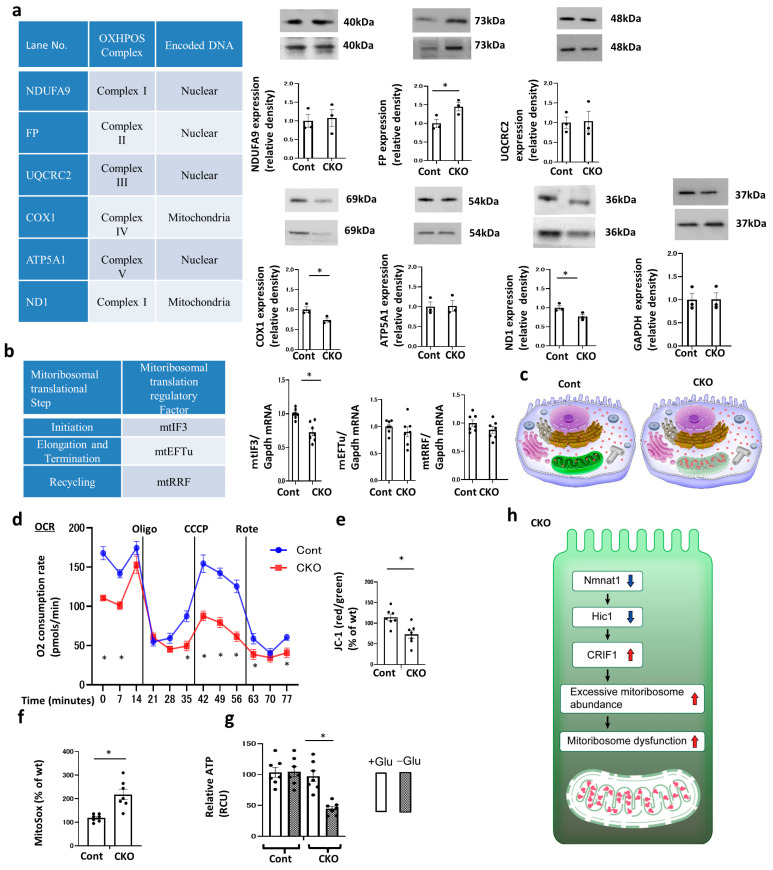
Mitoribosomal dysfunction and mitochondrial dysfunction in CKO mice. (**a**) Western blot analysis of renal protein levels of OXPHOS subunits encoded by nDNA and mtDNA. The kidney tissue specimens were obtained from CKO and control mice at 32 weeks of age. The results of a representative experiment among the three performed are shown. All data are indicated as the mean ± standard error of the mean (SEM). Horizontal bars denote statistically significant differences between the two groups. * *p* < 0.05. The bar graph in the lower panels illustrates the quantification of the band intensity. Glyceraldehyde 3-phosphate dehydrogenase was used as a control. N = 3 mice per group. (**b**) Real-time quantitative RT–PCR analysis of the renal mRNA of mitoribosomal translational regulators. Glyceraldehyde 3-phosphate dehydrogenase was used as a control. For RT–PCR, the kidney tissue specimens were obtained from CKO and control mice at 32 weeks of age. N = 7 mice per group. (**c**) Illustration depicting the dysfunctional mitoribosomes and their concomitant mitochondrial dysfunction in CKO mice. These functions are intact in control mice. (**d**) The oxygen consumption rate (OCR) of TECs isolated from CKO and control mice was measured using a Seahorse XF-24 flux analyzer. N = 3. (**e**) The ratio of red/green fluorescence of JC-1 in TECs isolated from CKO and control mice was used as a measure of mitochondrial membrane potential. N = 7. (**f**) Fluorescence of MitoSox in TECs isolated from CKO and control mice as a measure of mitochondrial levels of reactive oxygen species. N = 7. (**g**) ATP content in TECs isolated from CKO and control mice. N = 7. All data are presented as mean ± SEM. Horizontal bars indicate statistically significant differences between the two groups. * *p* < 0.05. (**h**) Scheme depicting the new mitoribosome excess-mediated mechanism of renal profibrotic changes and mitochondrial dysfunction in CKO mice. The downregulation of *Nmnat1* expression decreased HIC1 expression, resulting in increased CRIF1 expression and ultimately mitoribosome excess. The upregulation of mitoribosome excess leads to mitoribosomal dysfunction, deposition of collagen IV in addition to OXPHOS impairment and tubular mitochondrial dysfunction. MRPL13, mitochondrial ribosomal protein L13; MRPS15, mitochondrial ribosomal protein S15; VDAC, voltage-dependent anion channel; Cont, control; CKO, conditional knockout; OXPHOS, oxidative phosphorylation; ND1, NADH-ubiquinone oxidoreductase chain 1; NDUFA9, NADH: ubiquinone oxidoreductase subunit A9; FP, fluorescent protein; Cyto b, cytochrome b; UQCRC2, ubiquinol-cytochrome c reductase core protein 2; COX1, cytochrome c oxidase 1; ATP8, adenosine triphosphate 8; ATP5A1, ATP synthase F1 subunit alpha; mtIF3, mitochondrial translational initiation factor 3; mtEFTu, mitochondrial elongation factor EFTu; mtRRF, mitochondrial ribosome recycling factor; GAPDH, glyceraldehyde-3-phosphate dehydrogenase; CCCP, carbonyl cyanide m-chlorophenyl hydrazone.

We investigated whether mitoribosome excess is associated with mitoribosomal dysfunction to detect the expression of proteins synthesized by mitoribosomes; of these, mRNA was encoded by mit-DNA. Mitoribosomes generated the following oxidative phosphorylation (OXPHOS) subunits of complex I, III, IV, and V (except complex II): ND1 and COX1 (Figure 7a, Supplementary Figures S12–S23). The expression of these subunits was considerably reduced in CKO mice despite the increased number of mitoribosomes. However, the nuclear DNA-encoded and cytoribosome-synthesized OXPHOS subunits, such as NDUFA9, UQCRC2, and ATP5A1, remained unaltered in both CKO and control mice (Figure 7a). Thus, mitoribosome excess was associated with mitoribosomal function loss, possibly because of the high density of ribosomes, leading to a decrease in mitochondrial translational efficiency. Conversely, the expression level of FP, one of the important subunits of mitochondrial complex II (CII), was elevated in CKO mice. Therefore, we examined whether mitochondrial translational regulators, which mainly mediate three steps (initiation, elongation and termination, and recycling), were affected by CKO. Among these regulators, the expression level of mtIF3, a translational initiation regulator, was significantly reduced in CKO mice, whereas the expression levels of mtEFTu, which regulates elongation and termination, and mitochondrial ribosome recycling factor (mtRRF), which influences recycling, remained unaltered (Figure 7b). Thus, mitoribosome excess in CKO mice hindered mitoribosomal translational initiation and lowered the levels of mitoribosome-synthesized proteins related to OXPHOS CI, CIII, CIV, and CV (Figure 7a,b).

### 2.8. Impaired Mitochondrial OXPHOS Function in Nmnat1 CKO Mice

As mitoribosomes synthesize crucial OXPHOS proteins, such as CI-, CIII-, CIV-, and CV-related components, mitoribosomal dysfunction may induce mitochondrial dysfunction (Figure 7c). To investigate the influence of mitoribosome excess and its concomitant mitoribosomal dysfunction on mitochondrial dysfunction in CKO mice (Figure 7d), we analyzed oxygen consumption rate (OCR) and used JC-1 to determine mitochondrial membrane potentials and MitoSox red to determine the level of mitochondrial reactive oxygen species (ROS). We also examined ATP production by primary tubular epithelial cells (TECs) isolated and harvested from the kidneys of CKO and control mice. The OCR was lower in CKO mice than in control mice (Figure 7d), and the OCR in CKO mice did not respond to treatment with chemical inhibitors of mitochondrial respiratory chains; thus, CKO may have lost its OXPHOS activity in the basal state. The marked reduction in the incorporation of mitochondrial JC-1 (Figure 7e) into CKO cells suggested that the mitochondria were depolarized and dysfunctional. CKO cells exhibited higher mitochondrial superoxide levels, as shown in MitoSox analysis data (Figure 7f). 

In control cells, mitochondria were marked by punctate red fluorescence of JC-1 with green signal barely detected, indicating good mitochondrial membrane potential (Supplementary Figure S24a, control). In sharp contrast, CKO mice exhibited a drastic decrease of red JC-1 fluorescence in PT cells, with many cells displaying intense green monomer fluorescence (Supplementary Figure S24a, CKO). The shift from red to green JC-1 fluorescence indicated the loss of mitochondrial membrane potential in CKO cells. Moreover, immunofluorescence assays, with the use MitoSOX Red to quantify mitochondrial ROS production (Supplementary Figure S24b), revealed that CKO enhanced mtROS generation in PT cells. 

However, within 4 days after selection, the ATP levels in CKO cells did not differ from those in control cells grown in a high-glucose culture medium. Conversely, the ATP levels decreased in CKO cells cultured in a glucose-free medium; hence, enhanced glycolysis in CKO cells may be the main pathway for cellular ATP production (Figure 7g). Overall, mitoribosome excess in CKO cells enhanced OXPHOS dysfunction because of the loss of OXPHOS subunit (Figure 7h).

**Figure 8:**
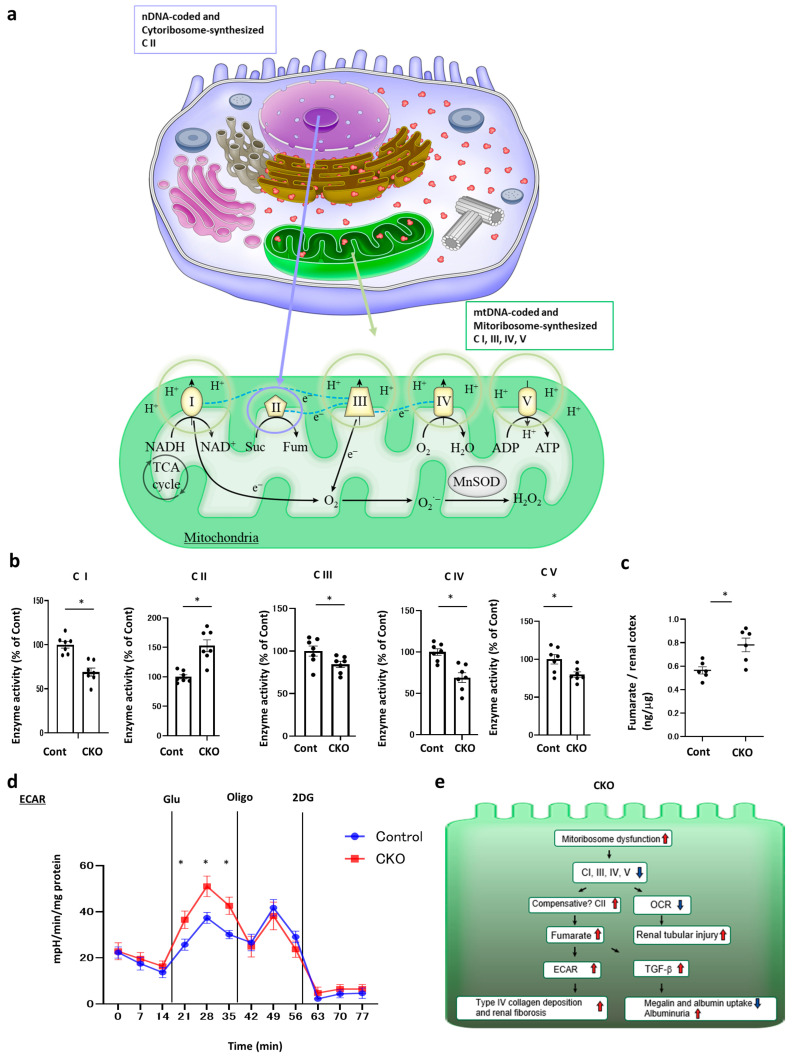
Measurement of mitochondrial electron transport chain (ETC) complex enzyme activities in the kidneys of CKO mice. (**a**) Scheme depicting the synthesis of OXPHOS complexes I–V (CI–CV). CII-related genes and proteins were only coded by nuclear DNA (nDNA) and cytoplasmic ribosomes (cytoribosomes), which were not influenced by mitochondrial DNA (mtDNA) and mitoribosomes. Conversely, mtDNA encodes and mitoribosomes synthesize CI-, CIII-, CIV-, CV-related genes and proteins, some of which were also encoded by nDNA and synthesized by cytoribosomes. Thus, mitoribosomes synthesize CI-, CIII-, CIV-, and CV-related component proteins but not CII-related proteins. (**b**) Complex activities were analyzed as described in the Methods section. All activity levels were calculated as the average of seven assays from each group of mice. Citrate synthase activity was used to normalize the level of mitochondrial proteins. Activities of CI–CV are plotted. All data are shown as the mean ± standard error of the mean (SEM). Horizontal bars indicate statistically significant differences between the two groups. * *p* < 0.05. The kidney tissue specimens were obtained from CKO and control mice at 32 weeks of age. (**c**) Fumarate levels in kidney cortex lysate from each group of mice were determined using the Fumarate Assay Kit. * *p* < 0.05 vs. control. N = 7 mice per group. (**d**) Glycolysis assay was performed. Glycolysis assay of extracellular acidification rate (ECAR) in primary renal tubular cells isolated from the kidneys of each group of mice (N = 3 independent experiments). Three chemical reagents were included: glucose (10 mM), oligomycin (1 μM), and 2-deoxyglucose (50 mM). Data are presented as mean ± SEM and were assessed using Student’s *t*-test (* *p* < 0.05 vs. Cont). The kidney tissue specimens were obtained from CKO and control mice at 32 weeks of age. (**e**) Scheme depicting mitoribosome excess-mediated renal tubular phenotypic changes in CKO mice. Mitoribosomal dysfunction decreased CI, CIII, CIV, and CV activity, which led to a reduction in oxygen consumption rate (OCR). Elevated CII activity might compensate for the reduction in CI, CIII, CIV, and CV activities. Considering that CII converts succinate into fumarate, the upregulation of CII activity increased local fumarate levels. In accordance with previous findings showing that fumarate enhanced TGF-β levels and glycolysis, renal TGF-β and ECAR levels were increased, both of which contributed to type IV collagen deposition and renal fibrosis. This result was consistent with previous findings demonstrating that ectopic tubular glycolysis and elevated local lactate production boosted renal fibrosis and type IV collagen deposition.

Ribosomes can be categorized into two types, cytoribosomes and mitoribosomes (Figure 8a). Mitoribosomes synthesize 13 OXPHOS proteins including CI-, CIII-, CIV-, CV-related components, except for CII; 2 rRNAs; and 22 tRNAs. Hence, major component proteins of CI, CIII, and CIV were synthesized in mitoribosomes, whereas CII-related proteins were synthesized in cytoplasmic ribosomes. We next examined whether CKO-induced mitoribosomal dysfunction decreases OXPHOS activity by measuring the activity of its complexes (Figure 8b). Notably, we found that CKO reduced the activities of CI, CIII, CIV, and CV but not that of CII. Therefore, the mitoribosome defect in CKO mice reduced the activities of CI, CIII, CIV, and CV. Contrary to the prediction that CKO does not influence CII, our results showed that CKO increased CII activity. Previous studies involving other tissues have revealed that CII may compensate for the defects in CI, CIII, CIV, and CV [13,14]. CII activity was based on a chemical conversion with succinate as the substrate and fumarate as the product, as shown in Figure 8a. Consistent with this, the upregulation of CII expression increased local fumarate levels in the kidneys of CKO mice (Figure 8c). Moreover, previous studies have revealed that fumarate enhances glycolysis [15]; hence, we measured extracellular acidification rate (ECAR) to provide more specific evidence regarding increased glycolytic flux in CKO mice. Based on the glycolytic capacity of CKO mice, glycolysis increased with increasing fumarate levels. However, mitochondrial inhibitors had no effect on glycolytic capacity. In CKO mice, mitoribosomal dysfunction caused mitochondrial dysfunction. Thus, mitochondrial inhibitors had no effect on these glycolytic conditions (Figure 8d). Taken together, our results showed that *Nmnat1* deficiency in CKO mice manifested as mitoribosomal dysfunction and inactivation of CI, CIII, CIV, and CV concomitant with the reduction in OCR. This might cause the compensative upregulation of CII activity, consistent with the elevated FP levels, as shown via immunoblotting (Figure 7a), and enhanced fumarate production. Reports have shown that fumarate elevates local TGF-β levels [15] as well as glycolysis [16] in the kidneys, which was also observed in CKO mice. These changes contributed to type IV collagen deposition and renal fibrosis in CKO mice (Figure 8e).

### 2.9. Protective Effects of Nmnat1 Overexpression on db/db-Induced Mitoribosome Excess and Mitochondrial OXPHOS Dysfunction

To assess the protective role of *Nmnat1* overexpression in the PTs with DN and compare with that of *Nmnat1* deficiency-induced diabetic mitoribosomal damage, and mice with PT-specific *Nmnat1* overexpression (*Nmnat1* TG) were developed (Supplementary Figure S25a,b). Further, we compared the kidney phenotypes in the four experimental groups, namely, WT-ND, TG-ND, WT-db/db, and TG-db/db (Supplementary Figure S25c). Immunofluorescence revealed that the *Nmnat1* levels were lower in WT-db/db mice than in WT-ND mice and significantly higher in TG-ND mice than in WT-ND mice (Supplementary Figure S25d). Similar results were obtained by RT–PCR for *Nmnat1* (Supplementary Figure S25e). Serum glucose levels did not differ between WT-ND and TG-ND mice or WT-db/db and TG-db/db mice at 8, 16, 24, and 32 weeks old (Supplementary Figure S25f). At the same ages, body weight also showed no significant difference between WT-ND and TG-ND mice or WT-db/db and TG-db/db mice (Supplementary Figure S25g). Serum creatinine levels were unaltered in all four experimental groups (Supplementary Figure S25h). However, TG-db/db mice showed reduced diabetic albuminuria compared with WT-db/db mice; hence, *Nmnat1* may have antialbuminuric effects in the PTs (Supplementary Figure S25i). SDS–PAGE analysis of urine protein excretion further demonstrated that TG had a protective effect against albuminuria (Supplementary Figure S25j). 

To determine the underlying mechanism of the protective effects of *Nmnat1* overexpression on albuminuria, we conducted immunostaining for albumin by using an antialbumin-specific antibody to examine changes in albumin reabsorption in proximal tubular cells. Compared with TG-db/db mice, WT-db/db mice exhibited reduced albumin staining in the intracellular tubular area, indicating reduced albumin reabsorption. The formation of intratubular albumin cast was increased in WT-db/db mice while inhibited in TG-db/db mice (Supplementary Figure S26a). The rate of type IV collagen deposition was significantly higher in WT-db/db mice than in WT-ND mice. However, these changes were attenuated in TG-db/db mice (Supplementary Figure S25b). Taken together, *Nmnat1* overexpression in the PTs has a protective role against db/db-induced renal peritubular fibrotic changes in addition to reducing db/db-induced albuminuria.

Morphological changes in intracellular organelles were further elucidated by assessing the EM findings. EM images of the mitochondria remarkably demonstrated that WT-db/db mice had more tiny black spots indicating mitoribosomes inside the mitochondria than WT-ND mice (Supplementary Figure S25c). The number of dense black spots in the cytoplasm corresponding to cytoribosomes remained unchanged in WT-db/db mice compared with that in TG-db/db mice (Supplementary Figure S25c). We confirmed the RT–PCR analysis results for correctly measuring the number of mitoribosomes by counting MRPs, as described in the following results (Supplementary Figure S26). The level of CRIF1, which regulates mitoribosomal number, was upregulated in WT-db/db mice, and this upregulation was improved in TG-db/db mice (Supplementary Figure S25d). In the immunostaining of Hic1, a regulatory transcriptional repressor of CRIF1, its levels were higher in TG-ND mice than in WT-ND mice. Additionally, the Hic1 levels were markedly lowered in WT-db/db mice than in WT-ND mice; this decrement was blocked in TG-db/db mice (Supplementary Figure S25e). Taken together, under basal state as seen in WT + ND or TG + db/db, Sp1 bound to the GC box that maintained CRIF1 transcriptional activity. Given that CRIF1 expression was detectable under basal state, the influence of Sp1 on CRIF1 basal expression may overcome the effect of Hic1 on CRIF1 transcriptional repression. Even when Nmnat1 was overexpressed (as observed in TG + ND), Sp1-mediated CRIF1 basal transcription may be superior to such repression. Therefore, CRIF1 expression was retained in TG + ND mice, consistent with the immunostaining result. In CKO or WT + db/db mice, Hic1 was downregulated, abolishing the Hic1-induced repression of CRIF1 expression. Therefore, CRIF1 expression was elevated (Supplementary Figure S25f). 

We evaluated MRPs to confirm whether mitoribosomes were maintained in TG-db/db mice. The expression levels of MRPL13 and MRPS15 markedly increased in WT-db/db mice; however, this increment was significantly inhibited in TG-db/db mice (Supplementary Figure S26a). We next investigated whether TG mice could be protected from diabetes-induced mitoribosome excess. In TG-db/db mice, mtDNA-encoded OXPHOS subunits (ND1, Cyto b, COX1, and ATP8) synthesized in mitoribosomes were significantly retained, whereas nDNA-encoded OXPHOS subunits (NDUFA9, FP, UQCRC2, COX4, and ATP5A1) synthesized in cytoribosomes remained unchanged (Supplementary Figure S26b). Taken together, TG-db/db retained the mitoribosomal function of synthesizing mt-DNA-encoded OXPHOS subunits. The reduction of mtIF3 levels caused by mitoribosome excess corresponding to mitoribosomal translational initiation loss was seen in WT-db/db mice but was reversed in TG-db/db mice (Supplementary Figure S26c). 

To examine the effect of mitoribosomal function preserved in TG mice on mitochondrial function (Supplementary Figure S26d), we examined OCR, JC-1, MitoSox, and ATP production in primary TECs isolated from the kidneys of four mouse groups, namely, WT-ND, TG-ND, WT-db/db, and TG-db/db mice. The OCR was lower in WT-db/db cells than in WT-ND cells, and the OCR in TG-db/db cells was conserved at similar values as in WT-ND cells. The OCR in WT-db/db did not respond to treatment with chemical inhibitors of the mitochondrial respiratory chain, suggesting the OXPHOS activity loss in WT-db/db mice under basal conditions (Supplementary Figure S26e). In contrast to TG-db/db cells, the remarkable decrease in the rate of mitochondrial JC-1 incorporation (Supplementary Figure S26f) in WT-db/db cells suggests that the mitochondria in these cells were depolarized and dysfunctional. The WT-db/db cells exhibited high levels of mitochondrial superoxide (Supplementary Figure S26g); these levels were also retained in TG-db/db cells. However, within 4 days after selection, the ATP levels in WT-db/db cells were the same as those in WT-ND cells grown in a high-glucose medium. Conversely, when the WT-db/db cells were grown in a glucose-free medium, the ATP levels decreased, suggesting that enhanced glycolysis in WT-db/db cells is a key pathway for cellular ATP production (Supplementary Figure S26h). However, this decrease was reversed in TG-db/db cells. These results also indicate elevated levels of glycolysis in WT-db/db mice, but these levels were suppressed in TG-db/db mice (Supplementary Figure S26h). Therefore, OXPHOS dysfunction in WT-db/db cells resulted from mitoribosome excess, but it was reversed in TG-db/db cells (Supplementary Figure S26i). In db/db or *Nmnat1* CKO, excessive mitoribosomes reduced translational efficiency because of mitoribosomes’ high density or overloaded baggage on the platform of tracks. In WT-ND, TG-ND, and TG-db/db mice, the number of mitoribosomes was appropriate; thus, mitoribosomes could constantly translate mitochondrial DNA-encoded proteins. Therefore, translated proteins were produced in a suitable producing speed (Supplementary Figure S26j) in these proximal tubules. 

In summary, the expression level of *Nmnat1* was downregulated in mice with db/db-induced DN, causing Hic1 expression reduction and subsequently, CRIF1 level increase and mitoribosome excess. Excessive mitoribosomes hindered mitoribosomal translation initiation and caused mitoribosomal dysfunction concomitant with OXPHOS impairment and type IV collagen deposition, as observed in *Nmnat1* CKO and WT-db/db mice. However, TG-db/db blocked these changes (Supplementary Figures S25–S34). To our knowledge, this study is the first to report on the new renal roles of *Nmnat1* in retaining mitoribosomal function.

## 3. Discussion

DN caused by db/db-induced diabetes was associated with reduced expression of *Nmnat1*. The role of *Nmnat1* deficiency was investigated by establishing PT-specific *Nmnat1* CKO mice that exhibited mitoribosome excess resulting from HIC1 defect-induced CRIF1 upregulation and increased albuminuria.

HIC1 is a sequence-specific transcriptional repressor [17]. It binds to a specific sequence named HiRE, which consists of a TGCC or GGCA core motif [18]. To the best of our knowledge, the role of renal HIC1 using in vivo models such as CKO mice remains unclear. However, recent reports have suggested that HIC1 is involved in renal pathophysiological mechanisms, which was mainly demonstrated using in vitro models [19,20]. HIC1 levels were downregulated in CKO (Figure 6d), contributing to increased CRIF1 levels and mitoribosome excess (Figure 6e). Thus, HIC1 may be an important regulator in DN pathophysiology.

The number of mitoribosomes was prominently increased (Figure 5c) in CKO mice. We initially hypothesized that these changes compensate for the mitochondrial dysfunction observed in DN. If this hypothesis is true, mitoribosomes may produce mitochondrial inner membrane-associated OXPHOS complex, especially CI, CIII, CIV, and CV, and counteract mitochondrial dysfunction to some extent. However, contrary to our prediction, increased number of mitoribosomes downregulated the levels of mitoribosome-synthesized ND1 (CI) and COX1 (CIV) (Figure 7a), leading to mitochondrial dysfunction (Figure 7d–h). Mitoribosomes synthesize CI-, CIII-, CIV-, and CV-related component proteins but not CII-related proteins [21]. According to previous studies, CII compensates for other OXPHOS complex deficiencies [13,14]. Correspondingly, the expression of FP, a CII component, was upregulated in CKO mice (Figure 7a) whereas that of mitoribosome-synthesized proteins was downregulated. Therefore, FP upregulation may be an indicator of CII’s compensation for other OXPHOS complex defects.

Mitoribosome excess observed in CKO mice caused mitoribosomal dysfunction because of the hindered mitochondrial translation initiation, leading to a decline in mtIF3 level (Figure 7b). Ribosomes exist as two types: cytoribosomes and mitoribosomes. Cytoribosome excess reportedly decreases translational efficiency [22,23]. Translational efficiency can be retained by synthesizing an appropriate number of ribosomes. Although there are no previous reports on the relationship between mitoribosome excess and mitoribosomal translation, extremely few or many mitoribosomes may disrupt mitochondrial translational efficiency (Figure 7a), similar to cytoribosomes. However, further studies are warranted to investigate whether only initiation factors, such as mtIF3, were affected by *Nmnat1* deficiency in CKO mice (Figure 7b) although other regulators of elongation and termination (mtEFtU) as well as recycling (mtRRF) remained unaltered. Although kidney-specific *mtIF3* gene-engineered mice have not yet been reported, mtIF3 CKO in the heart showed striking phenotypes of mitochondrial dysfunction [24], suggesting the important role of mtIF3 in mitoribosomal translation.

*Nmnat1* overexpression rescued mitoribosomal dysfunction caused by mitoribosome excess (Supplementary Figures S25–S34). Considering that *Nmnat1*-specific activators have not yet been explored [25], using NMNAT1 as a therapeutic medication in human DN seems challenging in the near future. However, adeno-associated virus (AAV) vectors using a PT-specific promoter have been reported [26,27] in animal models. If this method can be applied to human DN, *Nmanat1* AAV would be a useful vector. NAD boosters such as NMN and NR might be other candidates for elevating renal NAD levels as a therapeutic method for protecting against DN damages induced by mitoribosome excess.

This study has some limitations. First, we did not create HIC1 and CRIF1 CKO and TG mice. Therefore, the in vivo role of these molecules in kidneys should be further investigated. Second, the reason why mitoribosome excess in CKO mice specifically diminished mtIF3 was not elucidated. Future research is also warranted to investigate whether the levels of other translational initiation regulators besides mtIF3 were reduced in CKO mice. Although cytoribosome excess reportedly suppressed translational efficiency, the reason why mitoribosome excess mitigated mitoribosomal efficiency was not determined. In addition, it would be interesting to estimate the number or density of mitoribosomes that cause mitoribosome excess. We believe that these ideas are the goals of future studies. Third, we assessed db/db mice as a T2DM-induced DN model. Further research is warranted to test whether other type 2 diabetes mellitus (T2DM) models show a decrease in *Nmnat1* expression and whether *Nmnat1* overexpression or NAD elevation can rescue DN-induced albuminuria. Assessing other T2DM models may also clarify the mechanism by which *Nmnat1* was specifically reduced but *Nmnat3* was not affected in T2DM.

Therefore, future studies are warranted to unveil the mechanisms by which HIC1 was downregulated by reduced *Nmnat1* expression in CKO. However, some studies have revealed that a NAD-dependent deacetylase, Sirt1, controls HIC1 activity [28]. Therefore, some Sirt1-related regulatory systems in *Nmnat1* deficiency might decrease HIC1 expression and binding activity to the CRIF1 promoter region. Further research is also needed to examine whether CRIF1 silencing can block PT-specific Nmnat1 CKO phenotypic changes to confirm that CRIF1 is involved in Nmnat1 deficiency-mediated mitoribosomal excess.

*Nmnat1* mutation in retinal tissues can significantly damage optical functions [29]. According to a recent study on liver-specific *Nmnat1* CKO, mitochondrial dysfunction impairs insulin resistance [30]. Moreover, *Nmnat1* knockdown increases the damage caused by respiratory infections [31]. Another report suggested that *Nmnat1* plays a significant role in maintaining mitochondrial function [32]. Thus, *Nmnat1* may contribute to renal mitoribosome formation and mitochondrial function and exhibit various protective functions in other tissues. Recent studies also proposed that Nmnat1 is involved in pancreatic cancer and diabetic retinopathy [33,34]. 

In conclusion, *Nmnat1* downregulation was associated with mitoribosome excess and mitoribosomal dysfunction, leading to diabetic albuminuria and type IV collagen deposition. Interventions targeting this novel pathway may be a therapeutic strategy for DN.

## 4. Materials and Methods

### 4.1. Establishment of CKO Mice

Renal PT-specific *Nmnat1* CKO (*Nmnat1*–mnat1 function, leading to dia*Nmnat1* flox/flox mice on a C57BL/6J background with γGT-Cre mice (Jackson ImmunoResearch Laboratories, West Grove, PA, USA), as previously described [1]. This breeding process was used to establish the following three types of control mice: *Nmnat1* flox/flox, *Nmnat1* flox/+, and *Nmnat1* flox/+/Γgt-Cre tg/+. In the subsequent studies, *Nmnat1* flox/+/γGT-Cre tg/+ mice were crossed with *Nmnat1* flox/flox mice to establish *Nmnat1* flox/flox/γGT-Cre tg/+ (PT-specific *Nmnat1*^−/−^) mice (*Nmnat1* CKO mice), and *Nmnat1* flox/flox mice were used as control mice. We utilized db/db mice as diabetic models and db/+ mice as nondiabetic controls. All animal studies conformed to the animal experimentation guidelines of the School of Medicine in Tokushima University. All mice used in the experiments were males and housed individually at 23 °C on a 12-h/12-h light/dark cycle, with free access to food and water.

### 4.2. RNA Isolation, Reverse Transcription, and Quantitative PCR

Total RNA was extracted from cultured cells and tissues, using the RNeasy Plus Mini Kit (QIAGEN, Hilden, Germany). Real-time PCR was conducted using the ABI Prism 7700 Sequence Detection System (Applied Biosystems, Foster City, CA, USA) and SYBR GREEN System (Applied Biosystems). The relative mRNA level of each gene was normalized to that of the housekeeping gene glyceraldehyde-3-phosphate dehydrogenase (*GAPDH*). We used the following primers: *Nmnat1*, F(5′-GTGGAGACTGTGAAGGTGCTC-3′) and R(5′-GTGAGCTTTGTGGGTAACTGC-3′); *Nmnat2*, F(5′-TGGAGCGCTTCACTTTTGTA-3′) and R(5′-CGATCTCCTCATACCGCATC-3′); *Nmnat3*, F(5′-CACCAAACAGGAAGGTACCA-3′) and R(5′-AAGCCACCAGGTCTTTCTTC-3′); mitochondrial ribosomal protein L13 (MRPL13), F(5′-ACATAAACCTGTGTACCATG CAC-3′) and R(5′-GGTAGCCAGTATGCGAAGAGT-3′); mitochondrial ribosomal protein S15 (MRPS15), F(5′-ATCCGTTCTAGAAGCACCAAGAG) and R(5′-CTCAGCATAGCGTTGATAGTGAG); voltage dependent anion channel (VDAC), F(5′-CTCCCACATACGCCGATCTT-3′) and R(5′-GCCGTAGCCCTTGGTGAAG-3′); LaminB, F(5′-GGGCGTCAGATTGAGTATGAG-3′) and R(5′-TTAGAGAGCTGTGAGGAGAGG-3′); CR6-interacting factor 1 (CRIF1), F(5′-TATCTCCTGCGGCTCTCTGT-3′) and R(5′-CTTCTGCTTTCGCCAGTTTT-3′); and GAPDH, F(5′-CCAGGGCTGCTTTTAACTC-3′) and R(5′-GCTCCCCCCTGCAAATGA-3′); TGF-β, F(5′-GGACTCTCCACCTGCAAGAC-3′) and R(5′-GACTGGCGAGCCTTAGTTTG-3′); type IV collagen, F(5′-CTCCAGGTCCCTACGATGTC-3′) and R(5′-TCCAAAGGGTCCTGTCTCTC-3′); Hic1, F(5′-AACCTGCTAAACCTGGACCAT-3′) and R(5′-CCACGAGGTCAGGGATCTG-3′). 

### 4.3. Blood and Urine Examination

The urine albumin level was assessed using an enzyme-linked immunosorbent assay (Albuwell M; Ethos Biosciences, Newtown Square, PA, USA). The urine and serum creatinine levels were assessed using QuantiChrom™ Creatinine Assay Kit (BioAssay Systems, Hayward, CA, USA). Levels of renal NAD were measured using liquid chromatography–tandem mass spectrometry (LC–MS/MS), as described previously [5].

### 4.4. Histopathological Examination

The kidney tissue specimens of mice were fixed in 10% neutral-buffered formaldehyde, embedded in paraffin, and sliced into 4-μm thick sections. Immunohistochemistry was conducted as described previously [8]. Briefly, 4-μm thick paraffin sections were fixed in 3% formaldehyde and stained with primary antibodies against *Nmnat1*, *2*, *3*, CRIF1 (Proteintech, Chicago, IL, USA), HIC1, αSMA, fibronectin (Cell Signaling Technology, Danvers, MA, USA), albumin (Nordic-MUbio, Rangeerweg, The Netherlands), type IV collagen, megalin, cubilin, and amnionless (Millipore, Bedford, MA, USA). Furthermore, these sections were stained with biotin-labeled goat anti-rabbit immunoglobulin G (IgG) (Vector Laboratories, Peterborough, UK) or biotin-labeled anti-mouse IgG (Vector) and then treated using Vectastain Elite ABC Kit (Vector). Each image of the stained sections was scanned using a 3CCD camera (Olympus Optical, Tokyo, Japan). Masson’s trichrome staining was performed to detect the fibrous area of the kidneys. The percentage of the fibrotic area was calculated using ImageJ (National Institutes of Health, Bethesda, MD, USA). Two randomly selected fields per section of the kidneys were analyzed in each mouse, and the average of 20 sections was used for statistical analysis.

### 4.5. Mitochondrial Function

Mitochondrial function was assessed using isolated primary kidney epithelial cells from CKO or control mice. Such cells were isolated as described previously [1]. The endogenous cellular OCR was measured using an XF-24 extracellular flux analyzer (Seahorse Bioscience, Santa Clara, CA, USA) as per the manufacturer’s protocol. For measuring the mitochondrial membrane potential, we used the JC-1 mitochondrial membrane potential detection kit (Peninsula Laboratories, Inc., San Carlos, CA, USA) according to the manufacturer’s instructions. Moreover, the harvested cells were stained with MitoSOX red (Molecular Probes, Eugene, OR, USA) to determine the level of mitochondrial ROS, followed by ATP measurement, as described previously [1].

### 4.6. Measurement of ECAR

The mitochondrial ECAR was measured in primary proximal tubular cells using Seahorse XF Glycolysis Stress Test Kit (#103020-100, Seahorse Bioscience) and Seahorse XF-24 extracellular flux analyzer. Primary tubular cells were seeded into Seahorse XF Culture microplates. The calibration cartridge was hydrated at 37 °C in a non-CO_2_-containing incubator 1 day before the assay. The growth medium was replaced with Seahorse XF Base medium (#103193-100, Seahorse Bioscience) containing glutamine (1 mM), and the cells were incubated at 37 °C in a non-CO_2_-containing incubator for 1 h. To calculate the ECAR, three reagents were used: glucose (10 mM), oligomycin (1 μM), and 2-deoxyglucose (50 mM).

### 4.7. EM

For EM, the kidney tissue specimens were embedded into Epon epoxy resin. To evaluate PT morphometry, we randomly obtained electron micrographs of 10 PTs per kidney for each mouse.

### 4.8. Immunofluorescence Staining

We conducted dual labeling by incubating overnight 5-μm thick cryostat kidney sections mixed with two primary antibodies, namely, rabbit polyclonal anti-*Nmnat1* (1:50, 11399-1AP, Proteintech, Chicago, IL, USA) and mouse polyclonal anti-aquaporin-1 (1:100, B-11, Santa Cruz Biotechnology, Santa Cruz, CA, USA). The secondary antibodies were obtained from Jackson ImmunoResearch Laboratories, West Grove, PA, USA.

### 4.9. Luciferase Assay

A 1956-bp fragment (−1955 to +1) of the 5′-flanking region of *CRIF1* was isolated from the murine BAC genomic clone using the restriction endonucleases *BlnI* and *BcnI*. The *EcoRI*, *SmiI*, *SphI*, *EcoRV*, *Psp1406I*, and *EcoO109I* inserts from −1955 Luc region were subcloned to prepare the plasmids −1263, −813, −649, −496, −128, and −15 Luc. The CRIF1/pGL3 plasmids (−1955 Luc, −1263 Luc, −813 Luc, −649 Luc, −496 Luc, and −128 Luc) containing the murine CRIF1 promoter sequences between −1955, −1263, −813, −649, −496, and −128 and +1 were fused to a pGL3 vector (a firefly luciferase reporter plasmid) and then transfected with Lipofectamine 2000 (Invitrogen, Waltham, MA, USA). HK2 cells, which were previously described [7], were transfected with *Nmnat1* siRNA or control siRNA in a regular medium. Additionally, pRL-CMV (Renilla luciferase reporter vector; Promega, Madison, WI, USA) was cotransfected into the cells. At 24 h after the treatment, the medium was harvested. Luciferase activity was measured according to a previous report [8]. We also mutated HiRE at this site (TGCC to GATT) to abolish the binding function of HIC1. The mutagenesis primers were generated using the −649 luciferase reporter plasmid through in vitro mutagenesis.

### 4.10. NAD^+^ Metabolite Measurement

The tissues were homogenized with 3 volumes of methanol containing 6% perchloric acid and 4% phosphoric acid. Next, 3 volumes of methanol, including deuterated internal standard, were added to the tissue homogenate or serum samples. The mixture was vortexed and centrifuged. The supernatant was diluted with water and analyzed using LC–MS/MS. A Shimadzu Nexera UHPLC system (Shimadzu, Kyoto, Japan) consisting of an LC-30 AD pump, DGU-20A5R degasser, CTO-20AC column oven, and SIL-30ACMP autosampler was used. Separation was performed on a Triart C18 column (3.0 × 150 mm, 5 μm, YMC, Kyoto, Japan) at 50 °C. Mobile phase A contained water/formic acid/undecafluorohexanoic acid (1000/0.1/0.2, *v*/*v*/*v*), whereas mobile phase B comprised methanol. The chromatographic conditions were 0–4 min (5–80% B, 0.5 mL/min), 4–4.01 min (80–95% B, 0.5–1.0 mL/min), 4.01–7 min (95% B, 1.0 mL/min), 7–7.01 min (95–5% B, 1.0–0.5 mL/min), and 7.01–13 min (5% B, 0.5 mL/min). MS detection was performed using an API5000 triple quadrupole mass spectrometer (SCIEX, Framingham, MA, USA) with electrospray ionization (ESI) operated in positive ion mode. The ESI–MS/MS parameters were optimized using standard solutions for each analyte. Quantitation was performed via multiple reaction monitoring with the following transitions: *m*/*z* 123 → 80 for NAM, *m*/*z* 335 → 123 for NMN, and *m*/*z* 664 → 136 for NAD^+^.

### 4.11. Immunoblotting

Immunoblotting was performed as previously described [1] with specific antibodies against NADH:ubiquinone oxidoreductase subunit A9 (NDUFA9), flavoprotein (FP), ubiquinol-cytochrome c reductase core protein 2 (UQCRC2), ATP synthase F1 subunit alpha (ATP5A1), NADH-ubiquinone oxidoreductase chain 1 (ND1), *Nmnat1,* CRIF1 (Proteintech), and HIC1 (Cell Signaling Technology). The GAPDH band recognized by a specific antibody (Sigma-Aldrich, St. Louis, MI, USA) was used as the loading control. Band intensities were quantified using Scion Image Software 4.0.3.2 (Scion Corp, Frederick, MD, USA).

### 4.12. Enzyme Activities

Kidney mitochondria were isolated using differential centrifugation as described previously [1]. Enzyme activities of mitochondrial CI, CII, CIII, CIV, and CV were measured at room temperature using a Beckman Coulter DU 530 spectrophotometer (Beckman Coulter, Brea, CA, USA), as described previously [3]. Citrate synthase activity was measured at 412 nm (e = 13.6 mM^−1^ cm^−1^). Rotenone-sensitive CI, malonate-sensitive CII, antimycin A-sensitive (AA) CIII, KCN-sensitive CIV, and oligomycin-sensitive CV activities were investigated. All activity levels are expressed as the average of seven assays from each group of mice’s pooled samples. 

### 4.13. Cell Culture and Transfection

We cloned and transfected the Hic1 expression vector and treated the HK2 cells with small interfering RNAs (siRNAs) targeting Nmnat1 (Santa Cruz Biotechnology Inc., Santa Cruz, CA, USA), as previously described [30,35]. For the negative control, siRNA for green fluorescent protein (5′-GGCTACGTCCAGCAGCGCACC-3′) mRNA was used.

### 4.14. db/db Mice

We utilized db/db mice as diabetic models and db/+ mice as nondiabetic controls. To produce compound mutant mice with a *Nmnat1* TG *db*/*db* genotype, PT-specific *Nmnat1* TG (TG) mice were bred to male mice heterozygous for the *db* mutation (*db*/*m*). Both the *Nmnat1* TG and *db* heterozygous breeders were on a C57BL/6J genetic background. Offspring from the F1 generation, which were heterozygous for the *db* mutation and also expressed the *Nmnat1* transgene, were then bred to mice heterozygous for the *db* mutation. The F2 generation contained the desired compound mutant *(Nmnat1*-TG, *db*/*db*) and the other genotypes used. The following 4 genotypes were used: (1) wild-type nontransgenic (WT)-nondiabetic (ND), (2) TG-ND, (3) WT-*db*/*db*, and (4) TG-*db*/*db*. All of the mice used in the experiments were males and housed individually at 23 °C on a 12-h/12-h light/dark cycle, with free access to food and water. 

### 4.15. Statistical Analyses

All statistical data were analyzed using Prism 8 (GraphPad Software, San Diego, CA, USA). Data were expressed as mean ± standard error of the mean. We employed one-way analysis of variance and Tukey’s post hoc test for comparing several groups. A *p*-value of <0.05 was considered to indicate statistical significance.

## Figures and Tables

**Table 1:** Clinical data of patients with diabetic nephropathy. eGFR, estimated glomerular filtration rate; HbA1c, glycosylated hemoglobin.

Sample Name	Sex	Age (Years)	Serum Creatine (mg/dL)	Proteinuria (g/day)	eGFR (mL/min/1.73 m^2^)	HbA1c (%)	Fasting Blood Glucose (mg/dL)	Masson’s Trichrome-Positive Area	Nmnat1-Positive Area
DN-1	male	83	1.81	1.6	28.5	6.6	191	0.250577	0.17
DN-2	male	42	1.13	4.9	58.1	6.3	139	0.197075	0.24
DN-3	male	62	1.73	1.5	32.6	6.1	97	0.316498	0.069
DN-4	male	47	1.82	5.4	33.4	6.1	98	0.312996	0.19
DN-5	male	80	1.23	10.2	44.0	6.0	97	0.2414909	0.25
DN-6	male	56	2.93	12.0	18.9	6.4	144	0.170324	0.22
DN-7	male	75	1.57	1.4	33.6	6.1	205	0.1534656	0.32
DN-8	male	59	1.88	0.5	71.2	5.8	136	0.018735	0.41
DN-9	female	64	1.89	2.9	36.3	6.1	167	0.402166	0.03
DN-10	female	51	0.54	4.6	91.0	5.9	135	0.3484332	0.06
DN-11	male	61	0.69	7.7	89.0	7.2	155	0.098988	0.36

## Data Availability

The original contributions presented in the study are included in the article/Supplementary Materials, further inquiries can be directed to the corresponding author.

## Supplementary Materials

The following supporting information can be downloaded at: https://www.mdpi.com/article/10.3390/ijms25126384/s1.
