# Supplementary material for: Nmnat1 Deficiency Causes Mitoribosome Excess in Diabetic Nephropathy Mediated by Transcriptional Repressor HIC1
Source: Int J Mol Sci. 2024 Jun 9;25(12):6384. doi: 10.3390/ijms25126384 (PMC11204038; doi:10.3390/ijms25126384)
Supplement: Supplementary file 1 [file ijms-25-06384-s001.zip › ijms-2970712-supplementary.pdf]

a Nmnat2

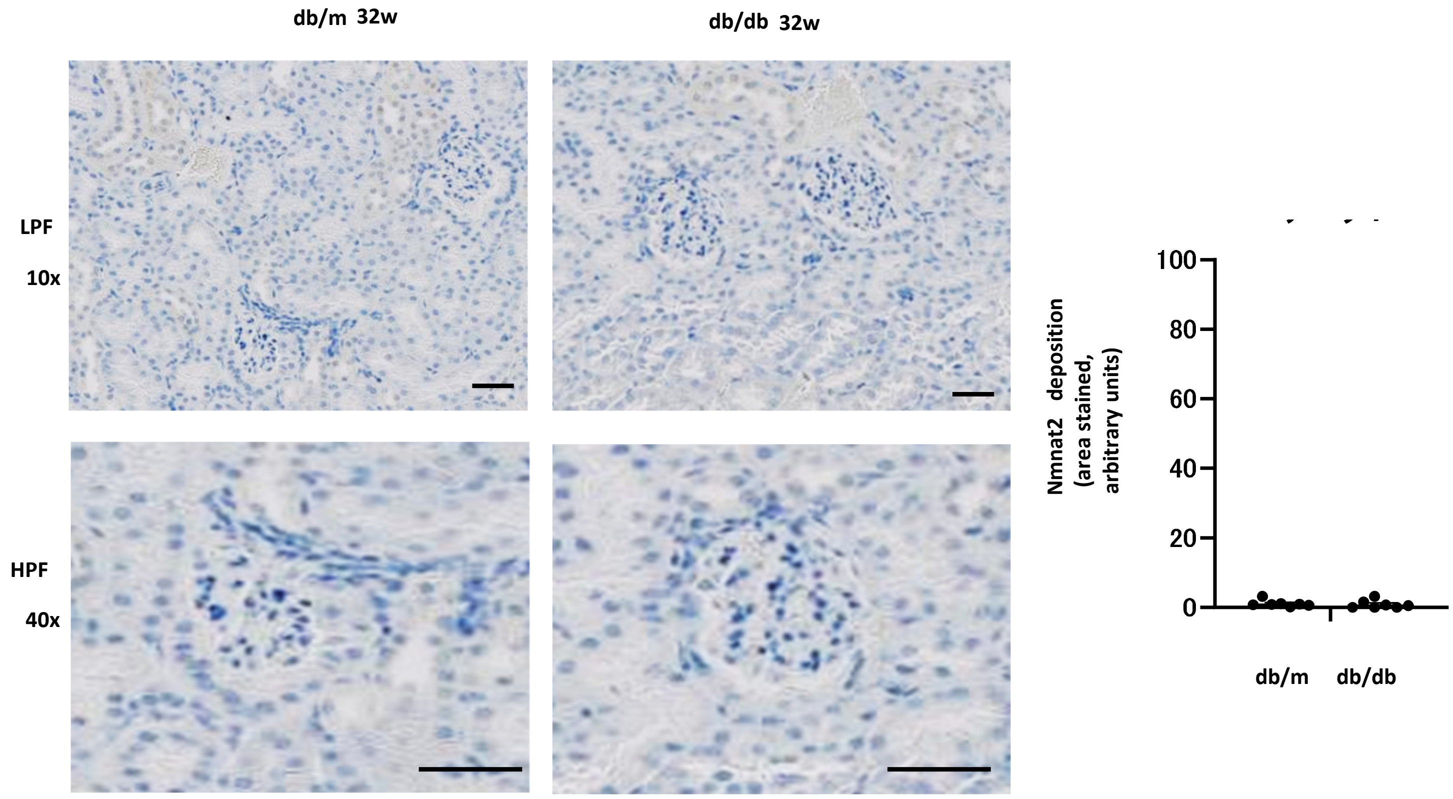

b Nmnat3

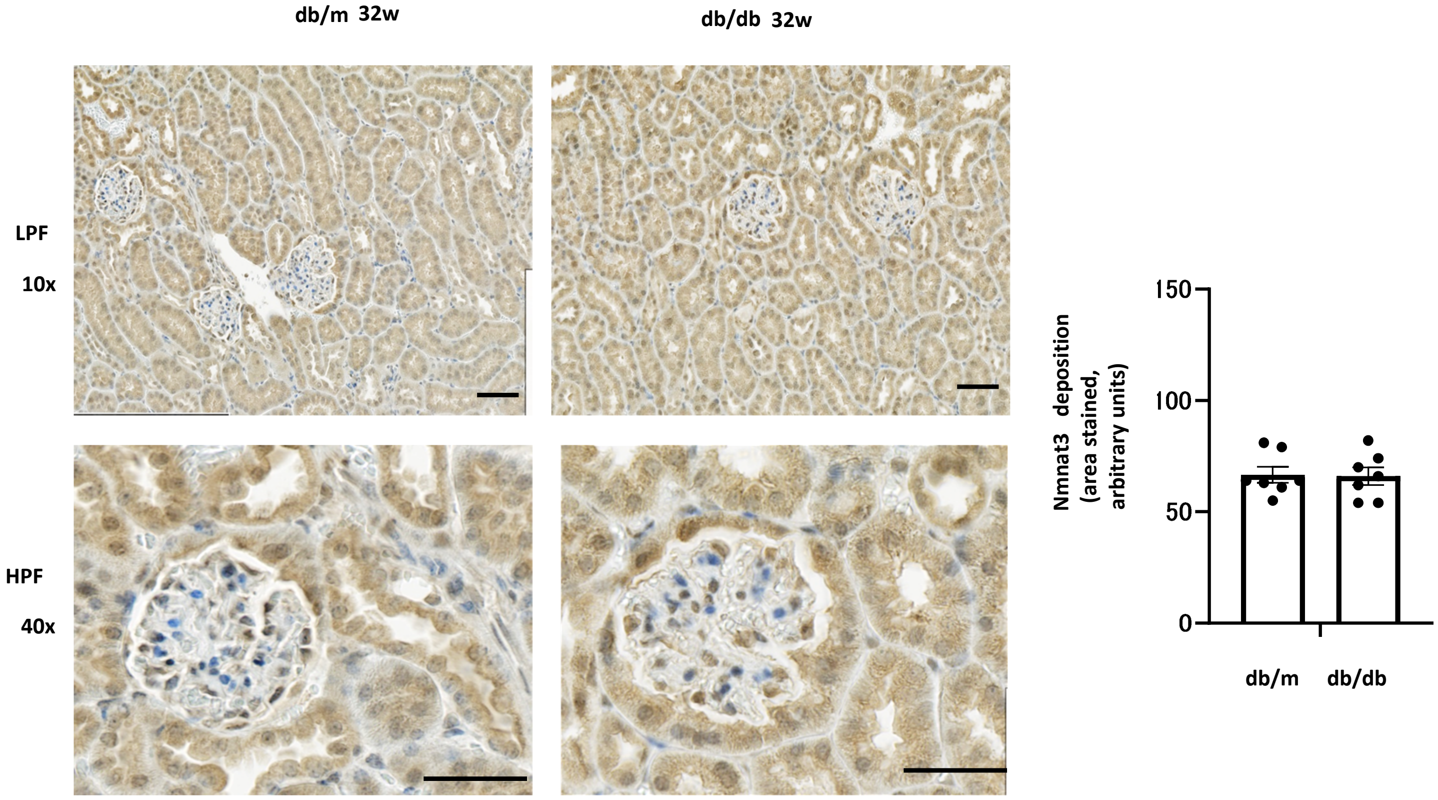

Supplementary Figure S1

a **eGFR vs Nmnat1**

R^2=0.07239

P=0.4237 Not significant

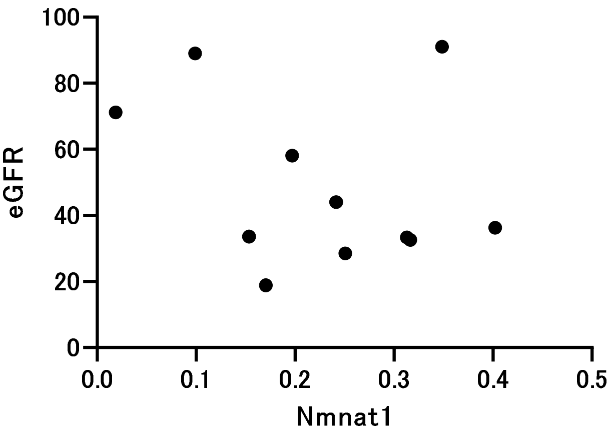

b **Proteinuria vs Nmnat1**

R^2=0.002910

P=0.8748 Not significant

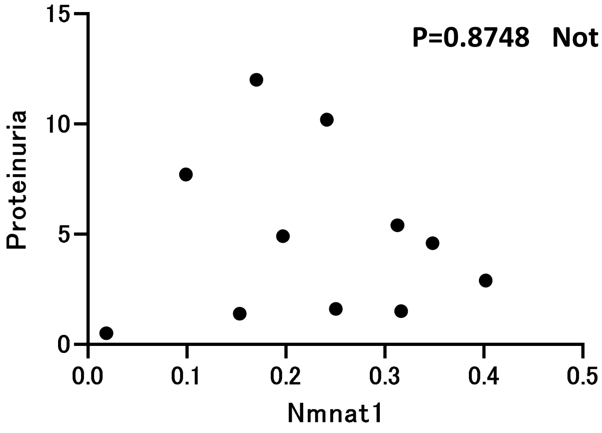

c **Serum creatinine vs Nmnat1**

R^2=0.004098

P=0.8517 Not significant

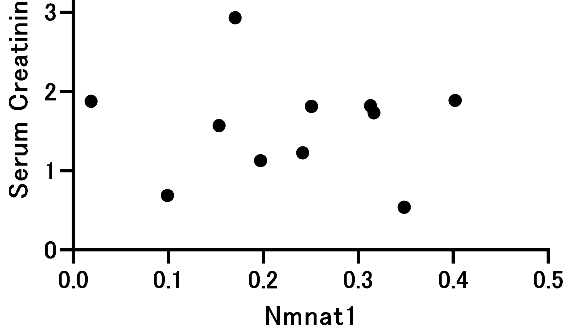

Supplementary Figure S2

a

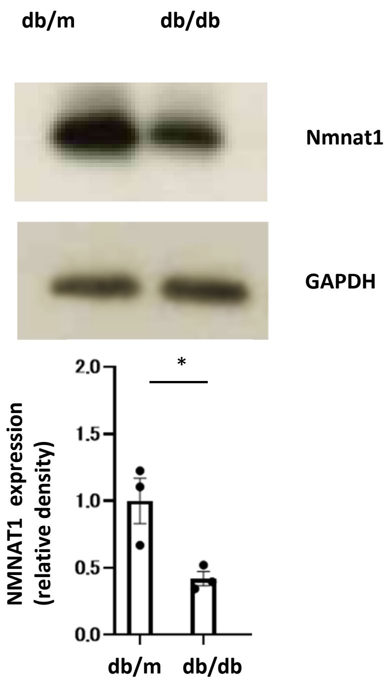

b

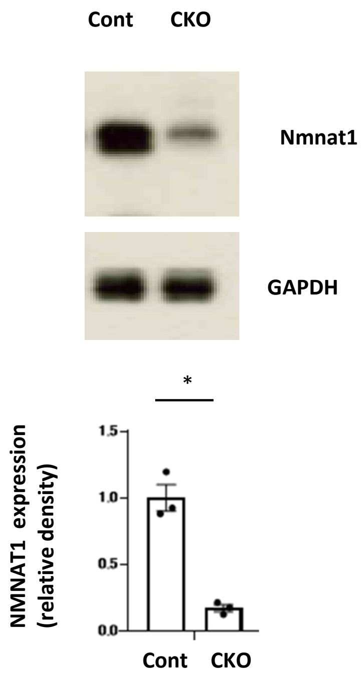

c Uncropped blots of a and b

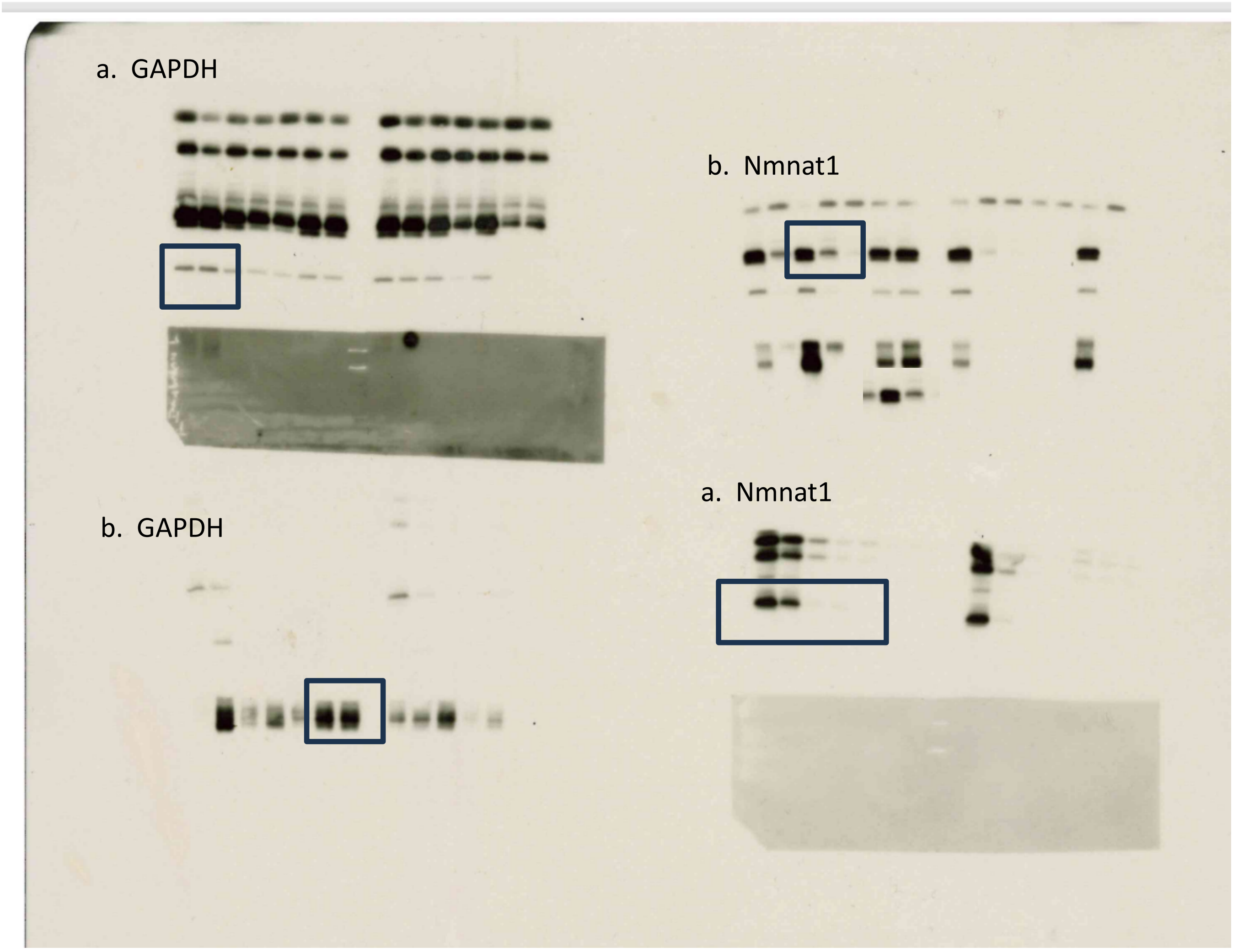

Supplementary Figure S3

a

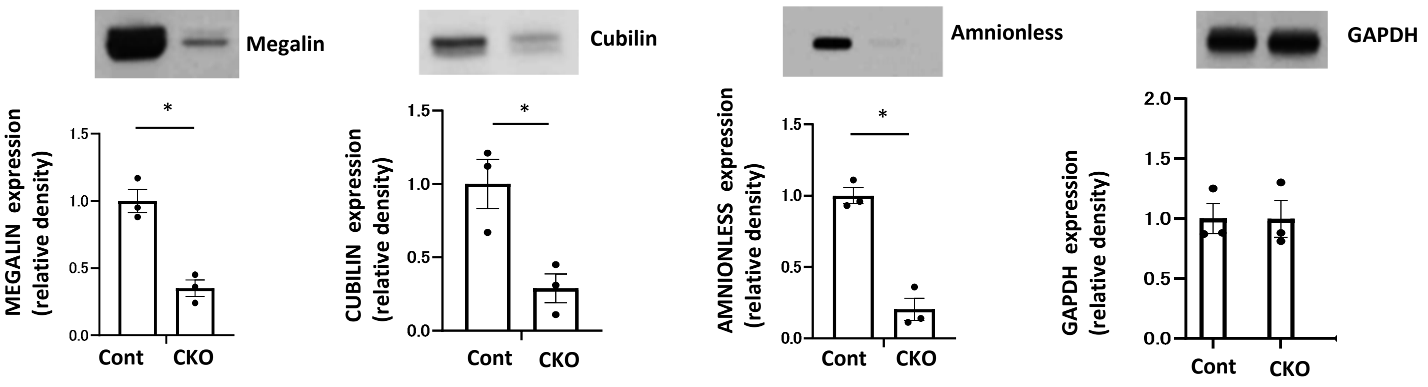

b Uncropped blots of a

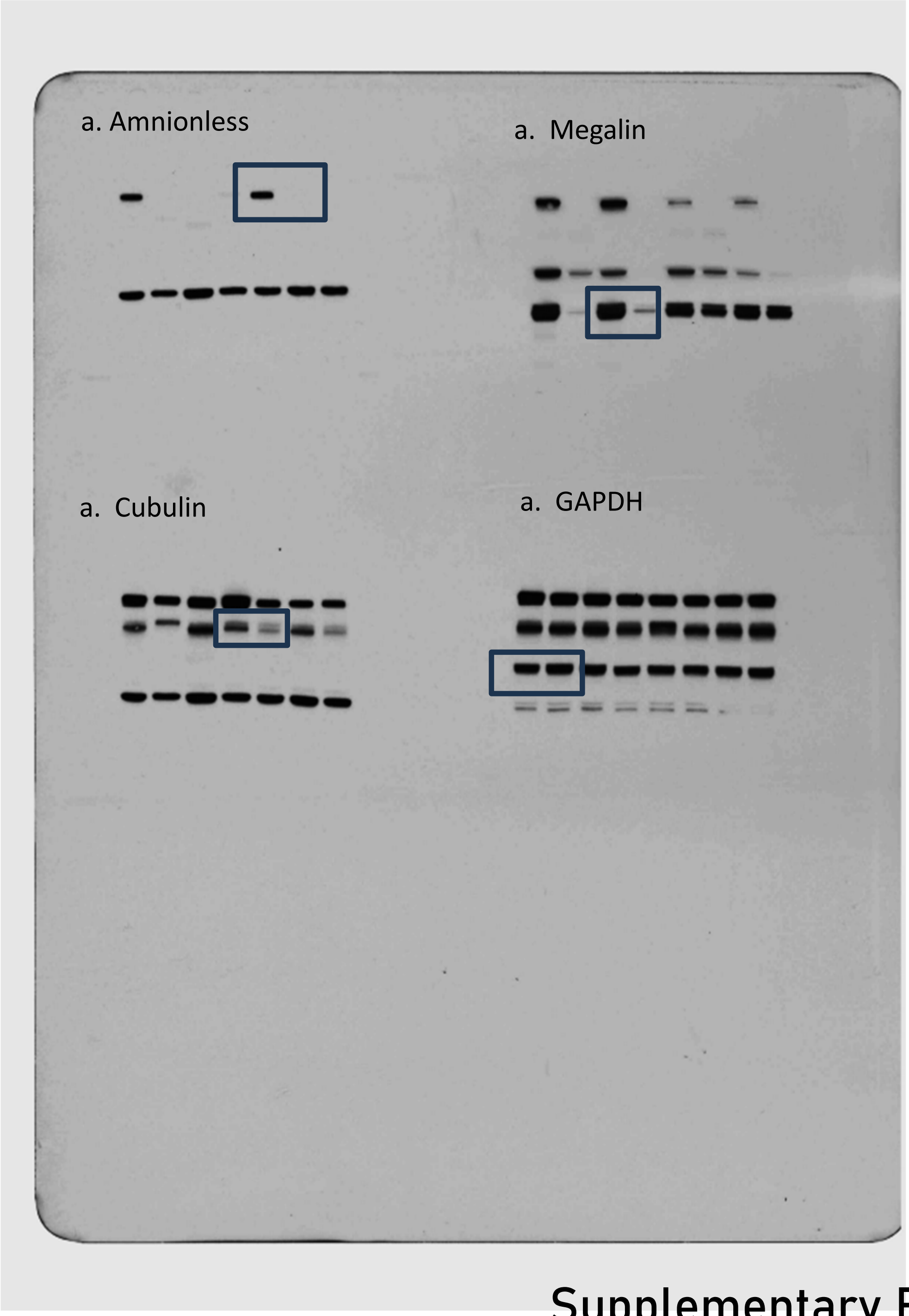

Supplementary Figure S4

TGF-β PCR

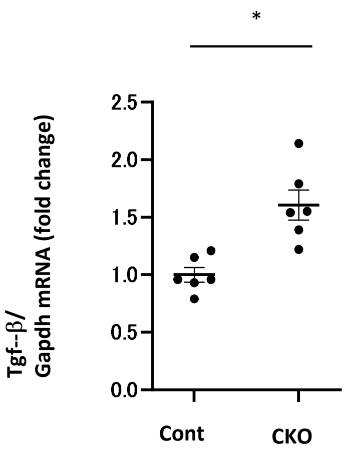

Type IV col

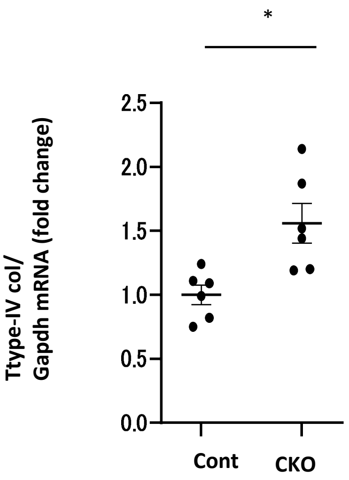

Supplementary Figure S5

a

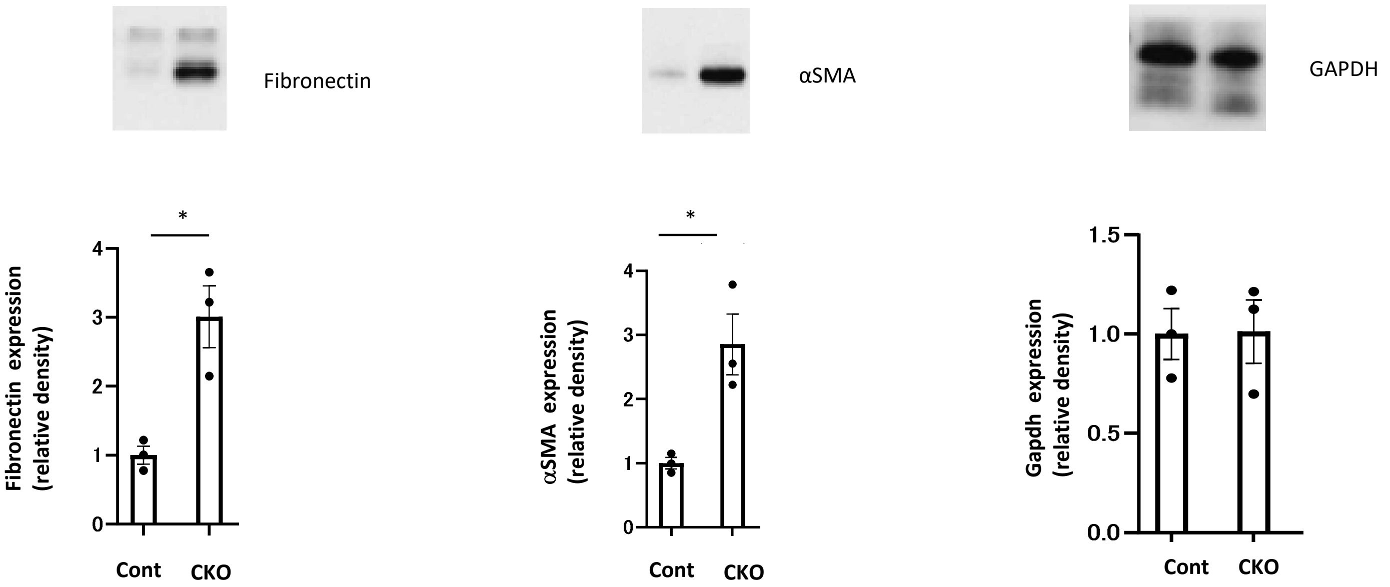

b Uncropped blots of a

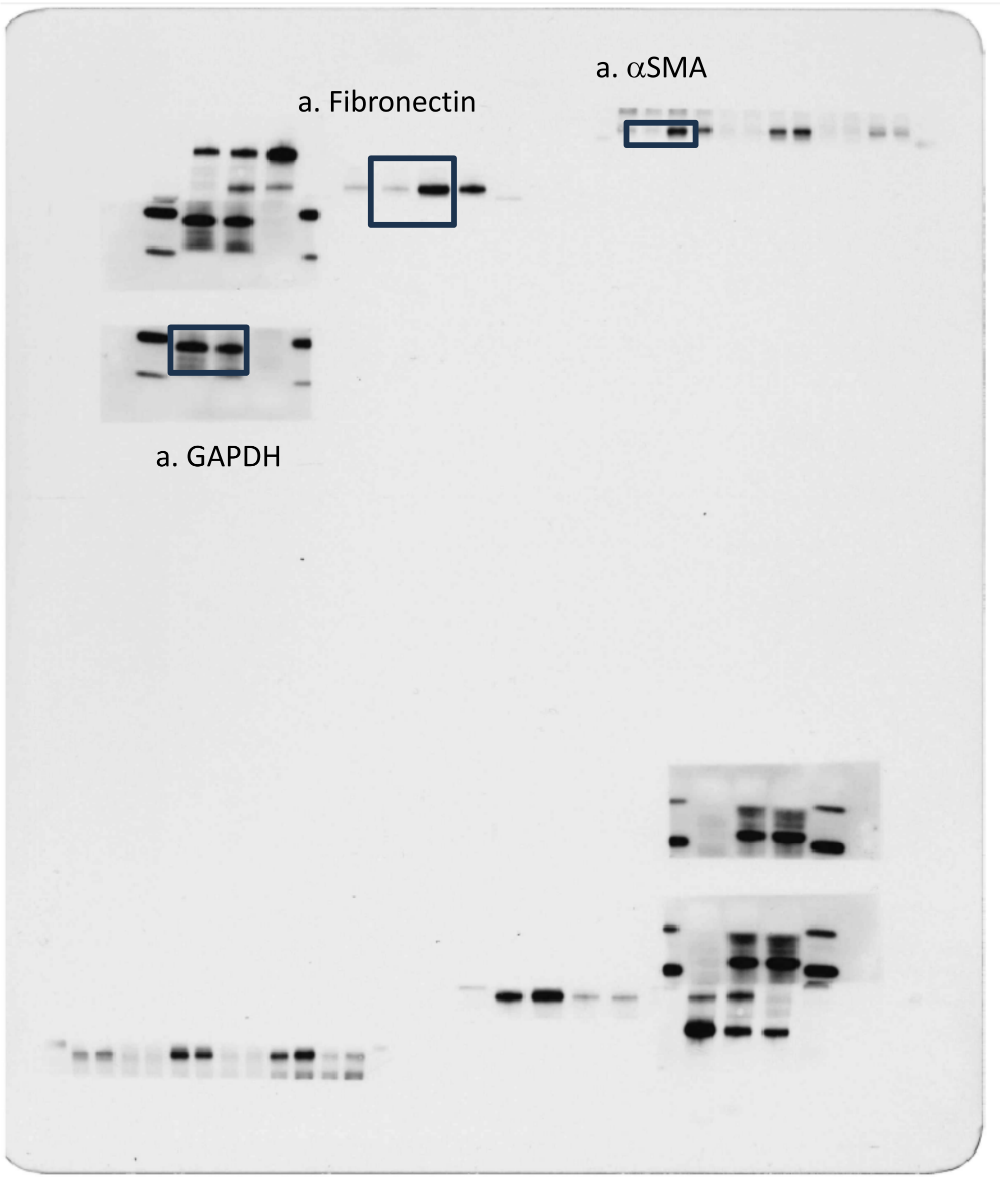

Supplementary Figure S6

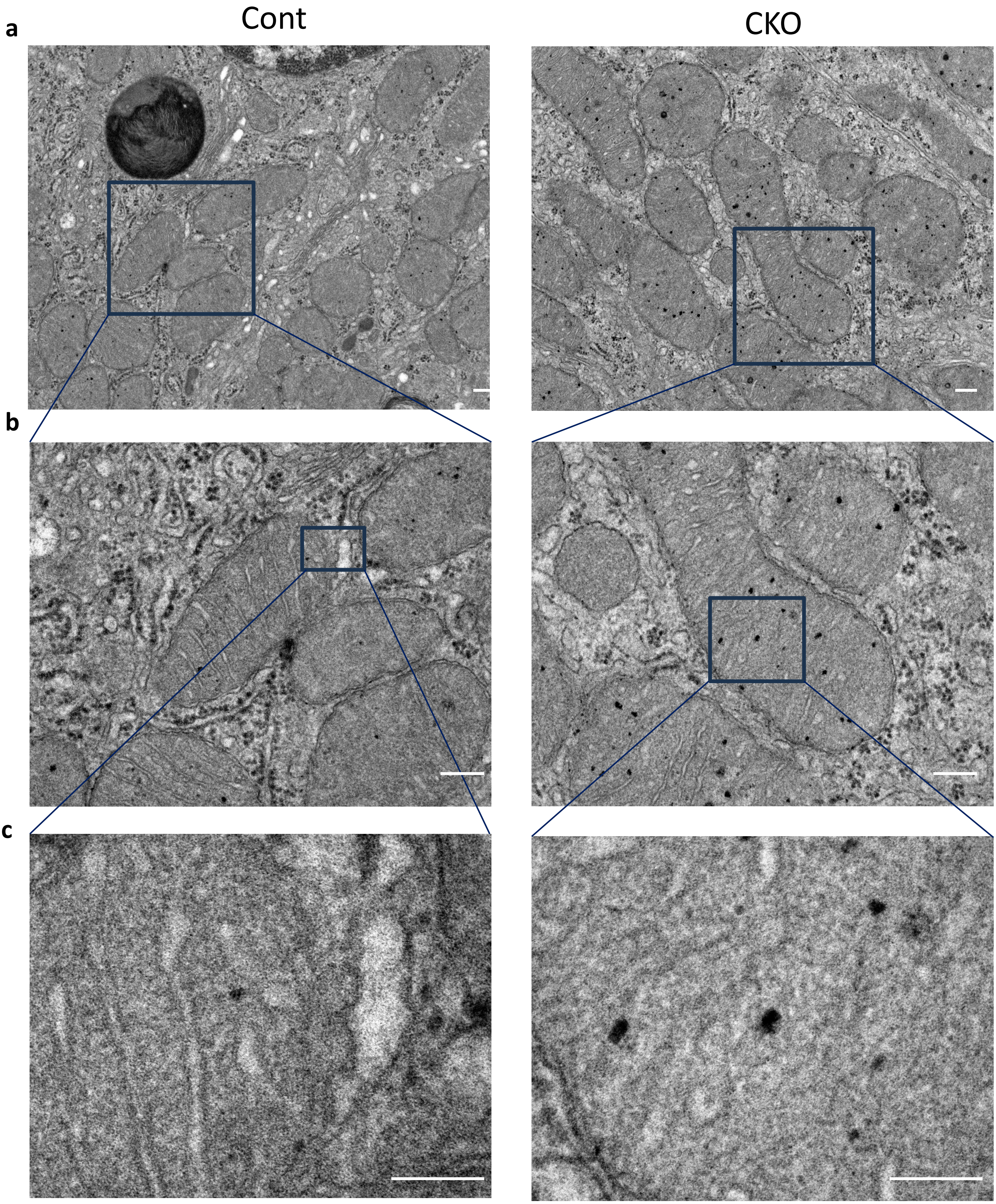

Supplementary Figure S7

a

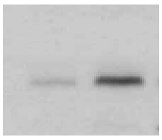

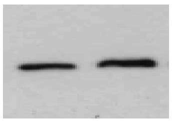

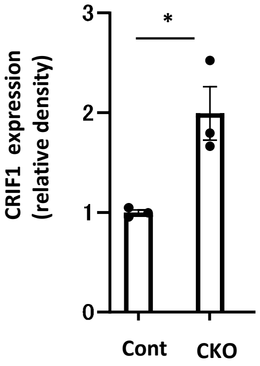

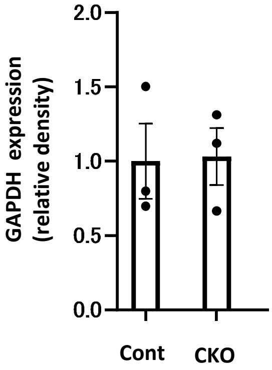

b **Uncropped blots of a**

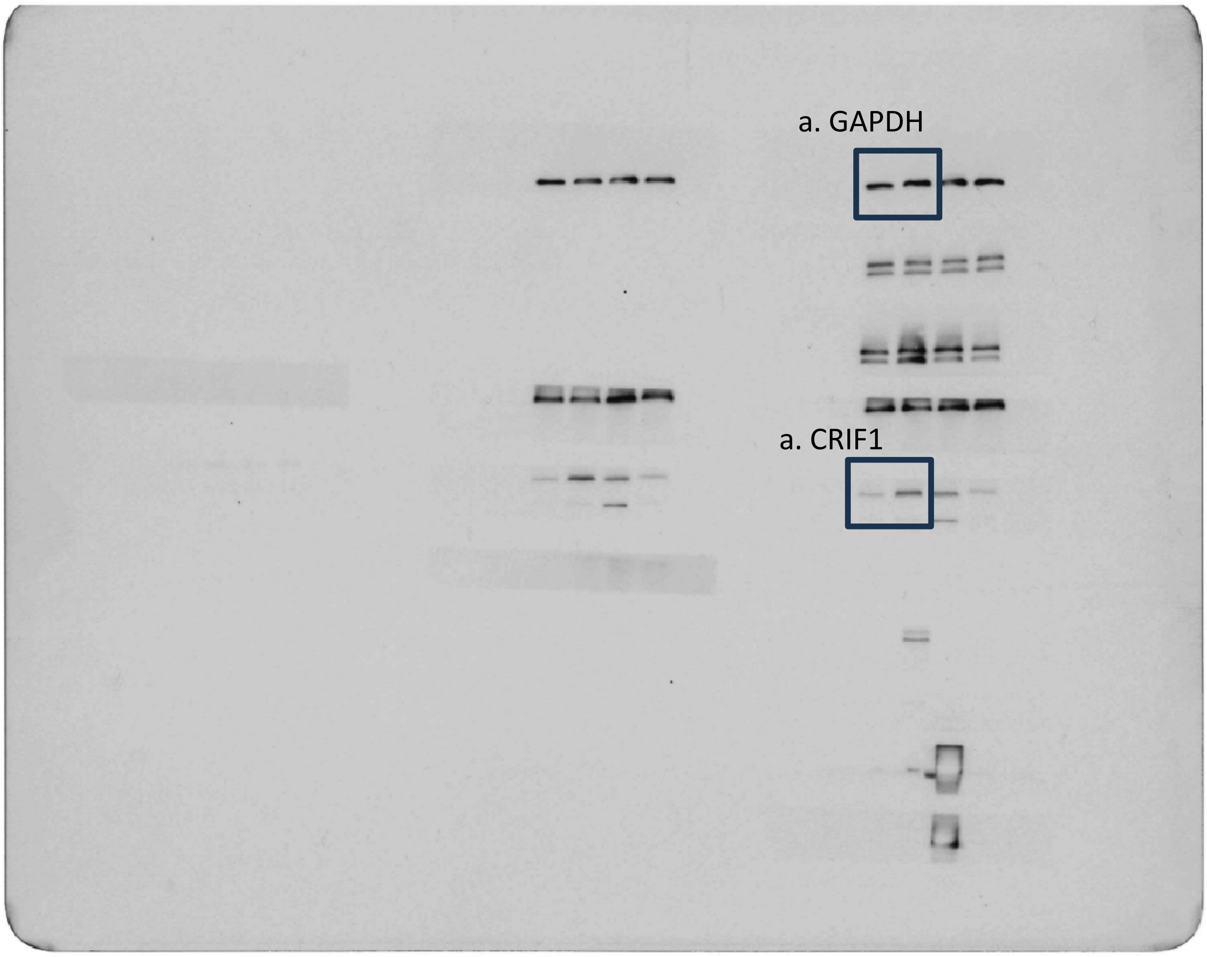

Supplementary Figure S8

Hic1 PCR

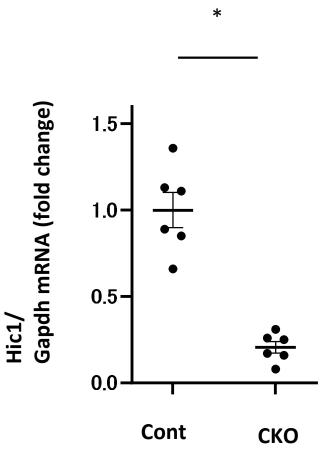

Supplementary Figure S9

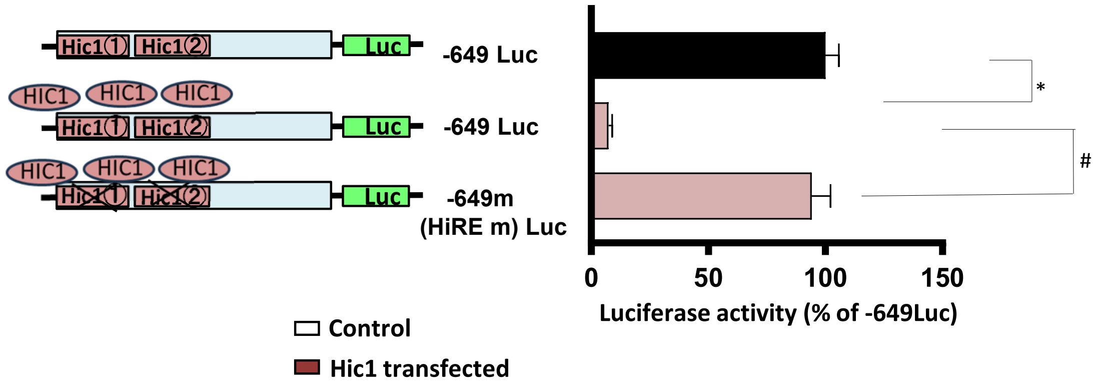

Supplementary Figure S10

a

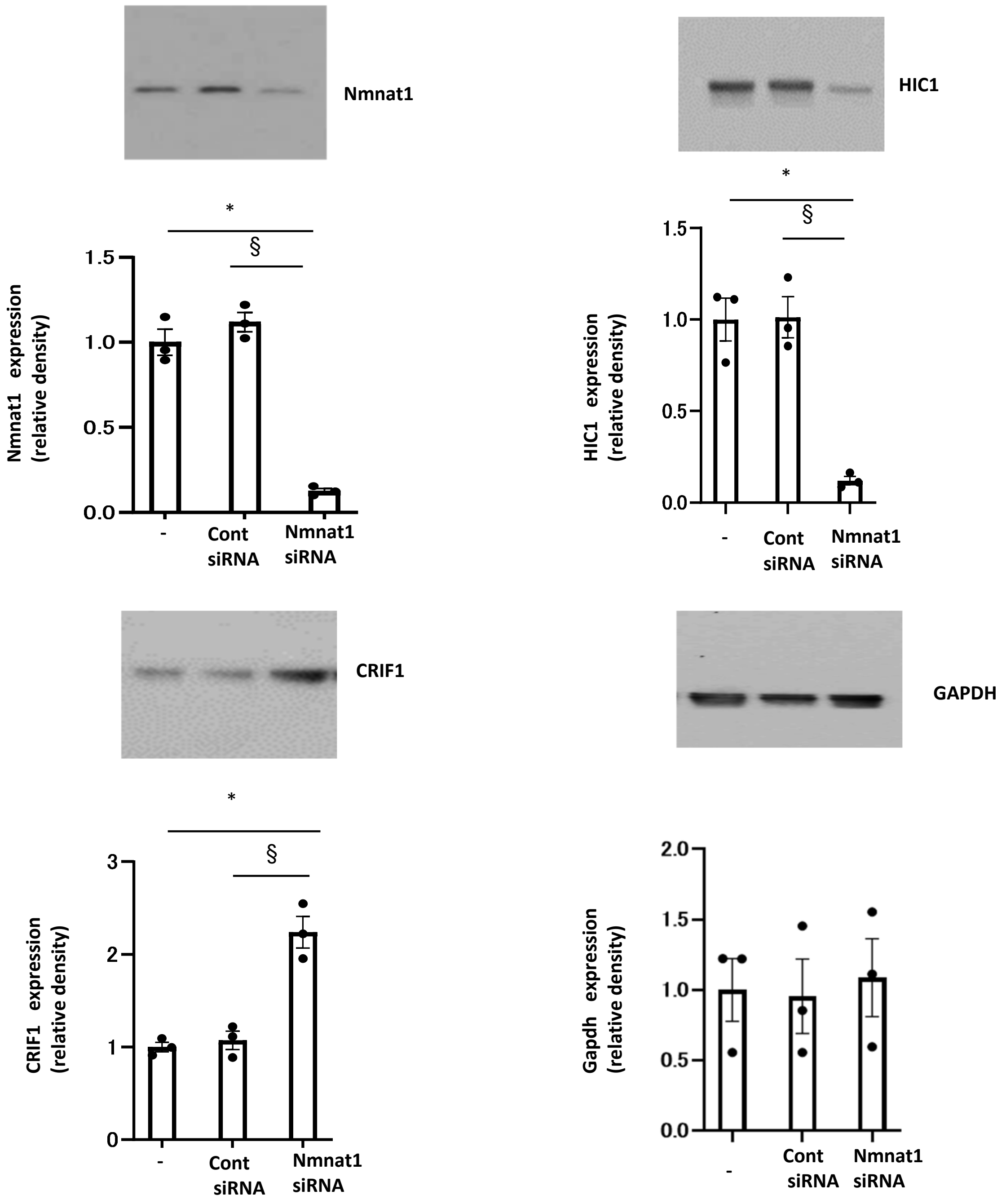

b Uncropped blots of a

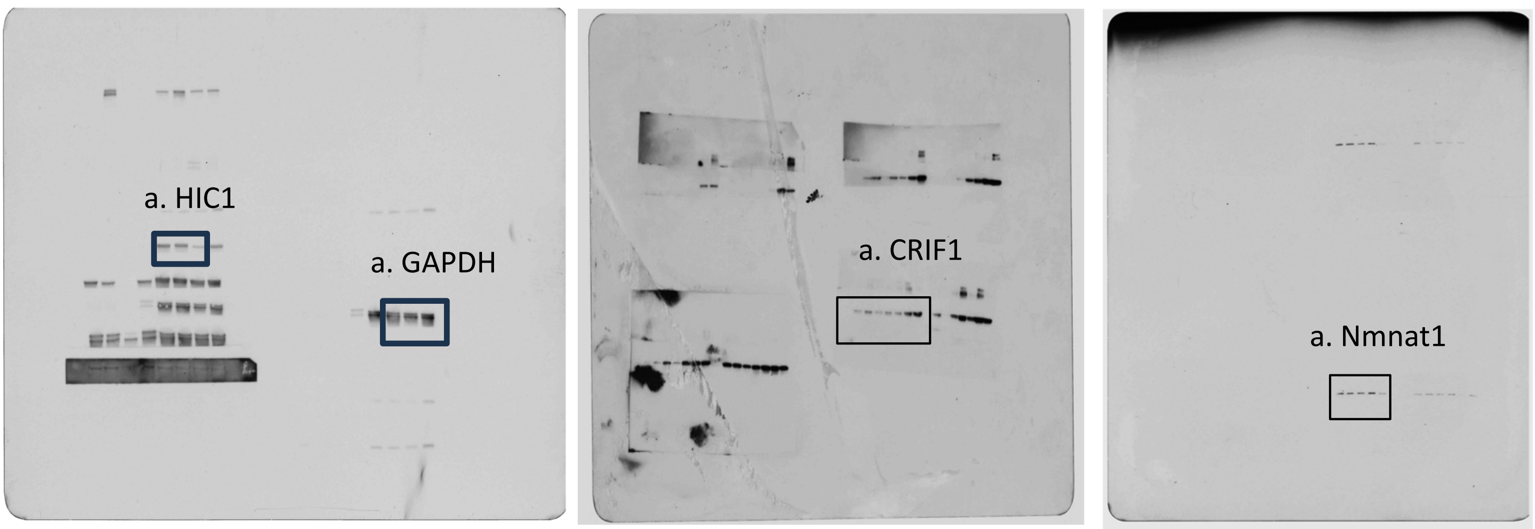

Supplementary Figure S11

Uncropped blots of NDUFA9 in Figure 7a

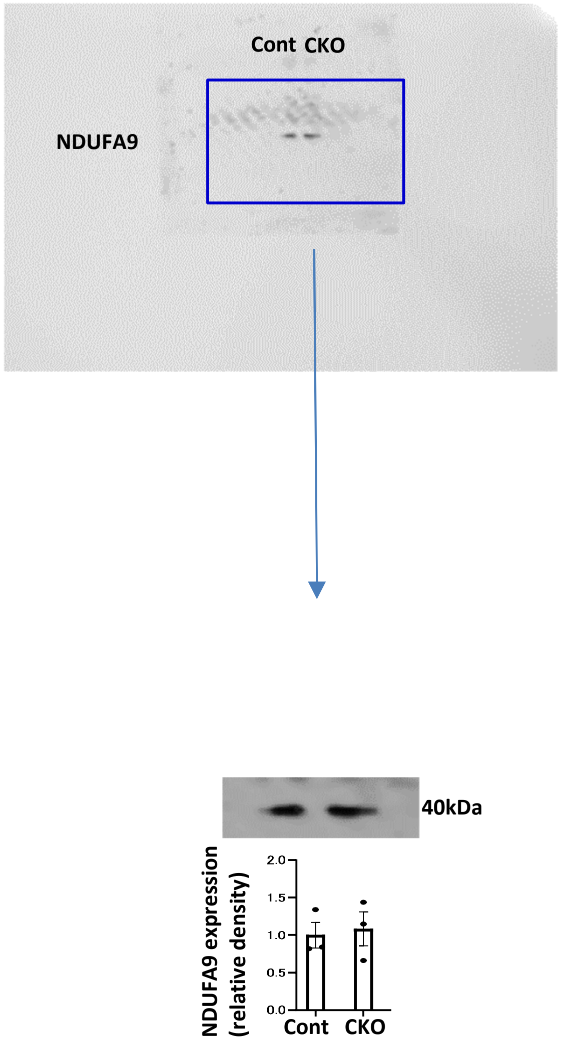

Supplementary Figure S12

Uncropped blots of FP in Figure 7a

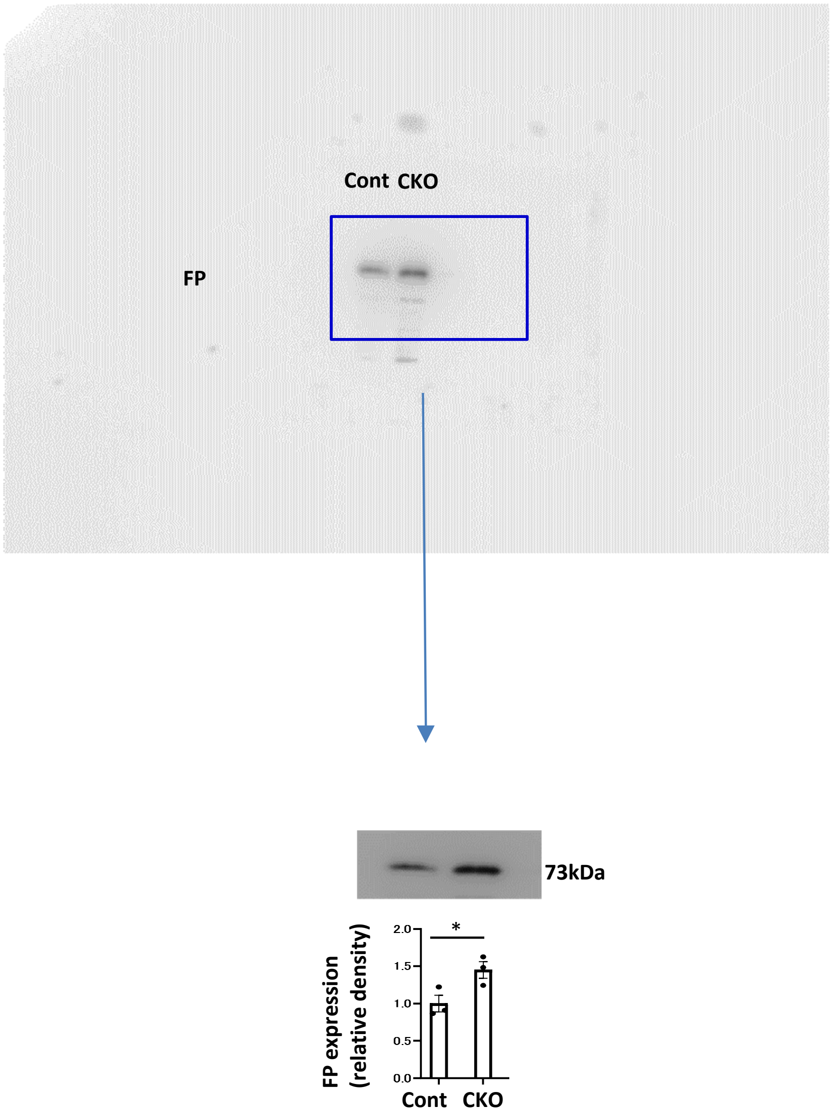

Supplementary Figure S13

Uncropped blots of UQCRC2 in Figure 7a

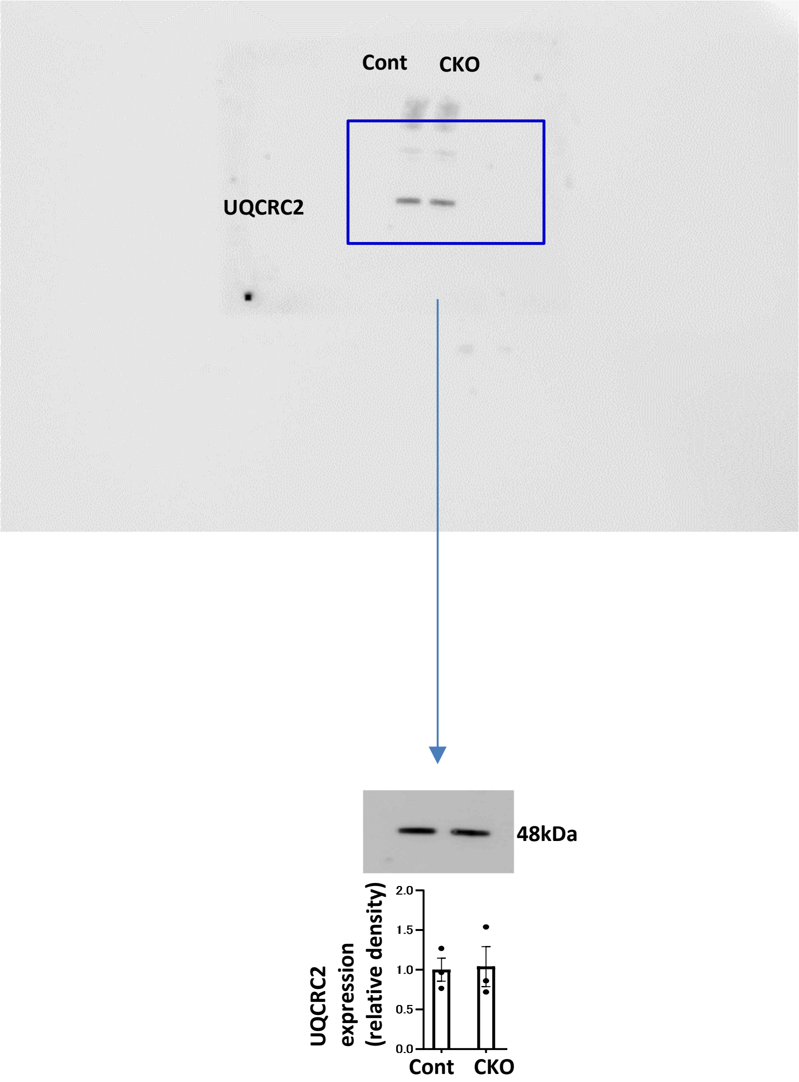

Supplementary Figure S14

Uncropped blots of COX1 in Figure 7a

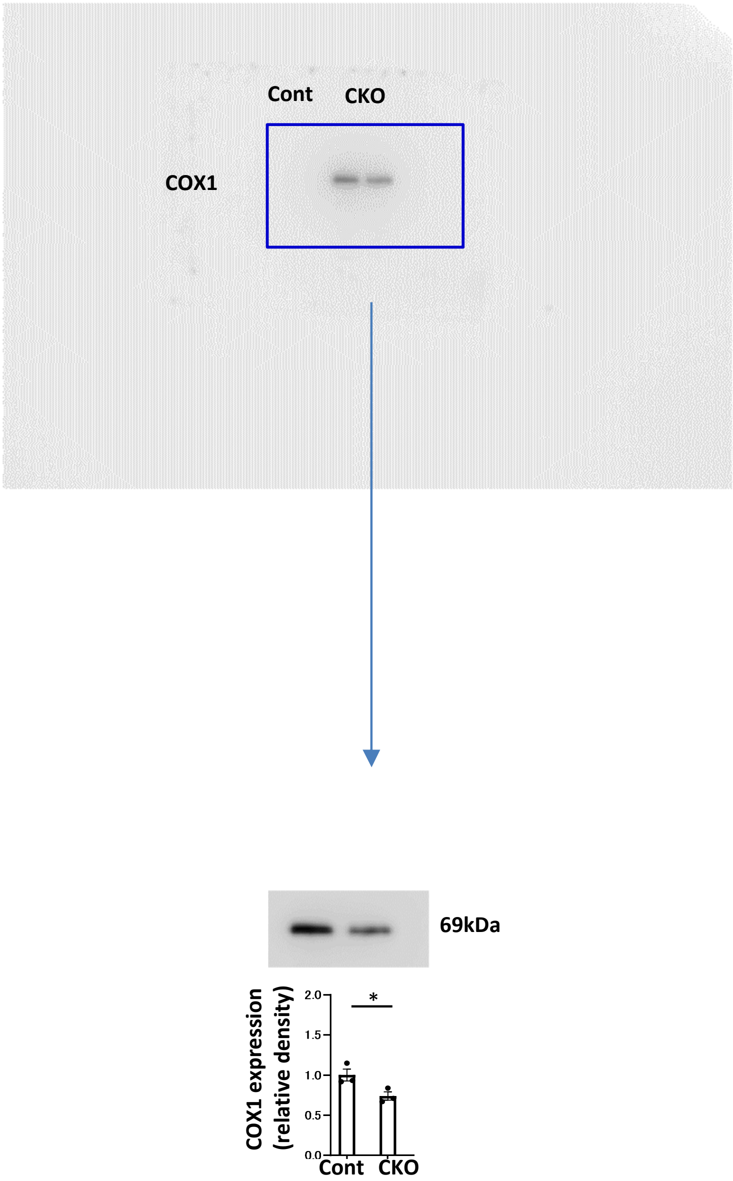

c

Supplementary Figure S15

Uncropped blots of ATP5A1 in Figure 7a

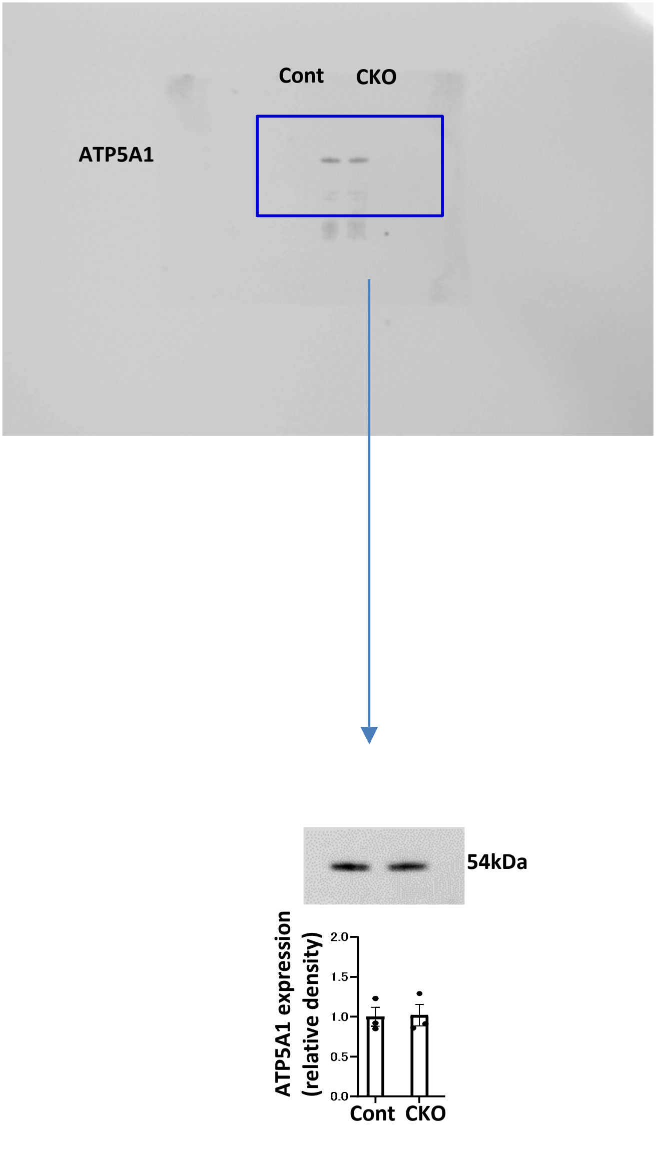

Supplementary Figure S16

Uncropped blots of ND1 in Figure 7a

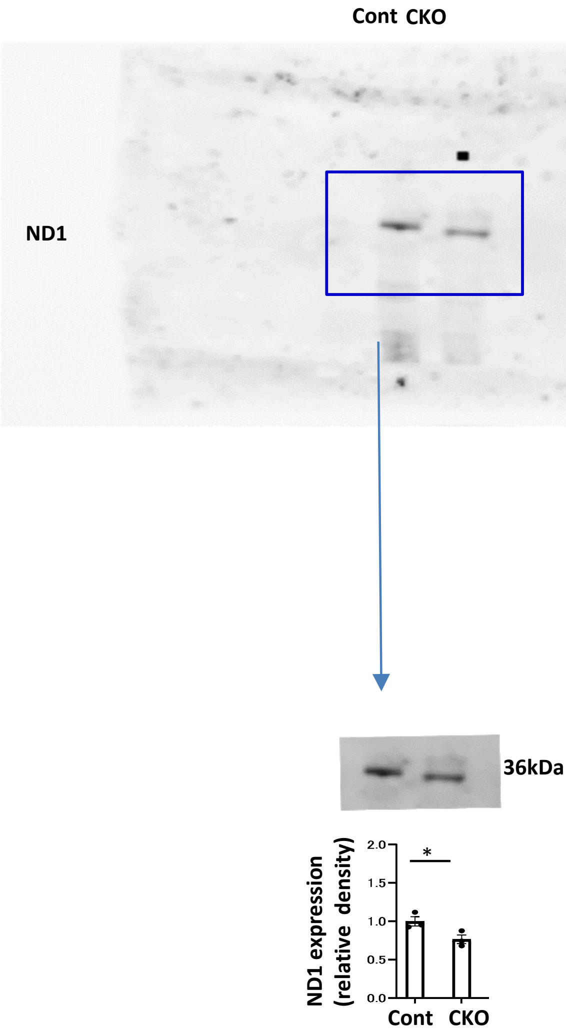

Supplementary Figure S17

Uncropped blots of GAPDH in Figure 7a

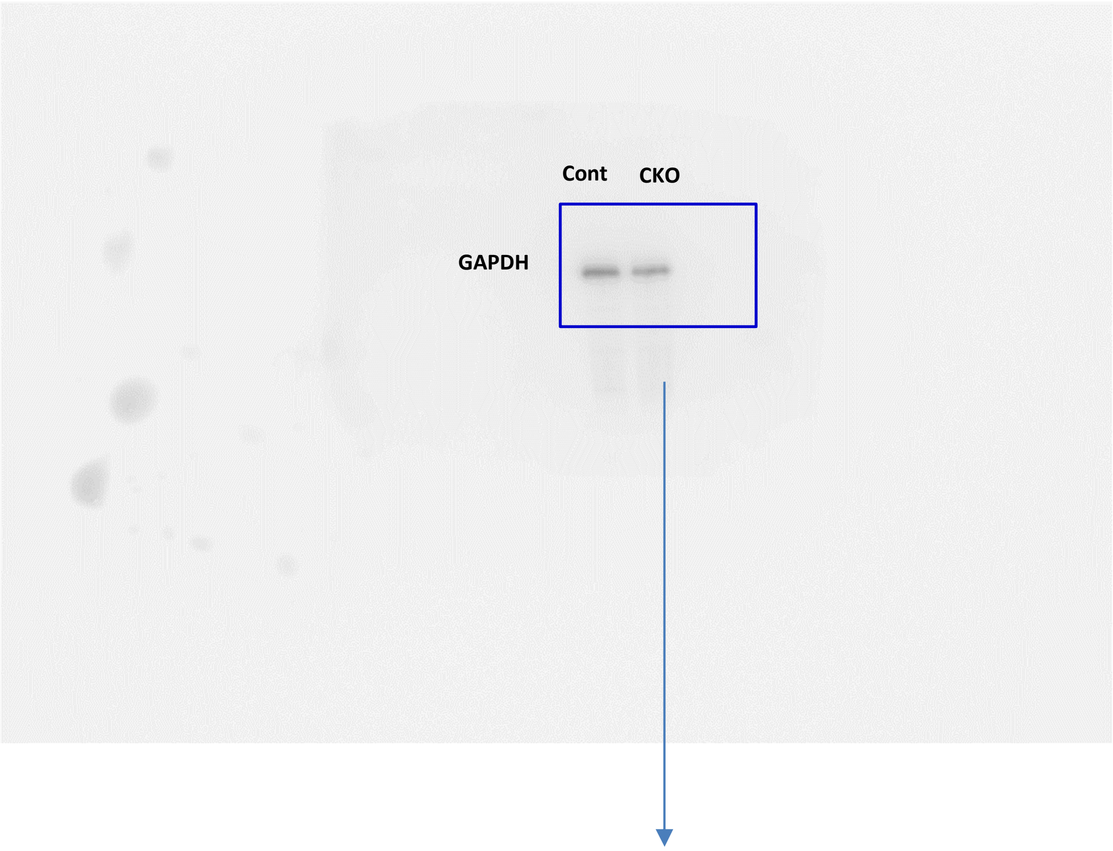

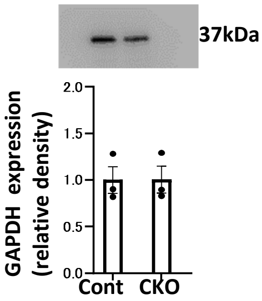

Supplementary Figure S18

Second set of uncropped blots of NDUFA9 in Figure 7a

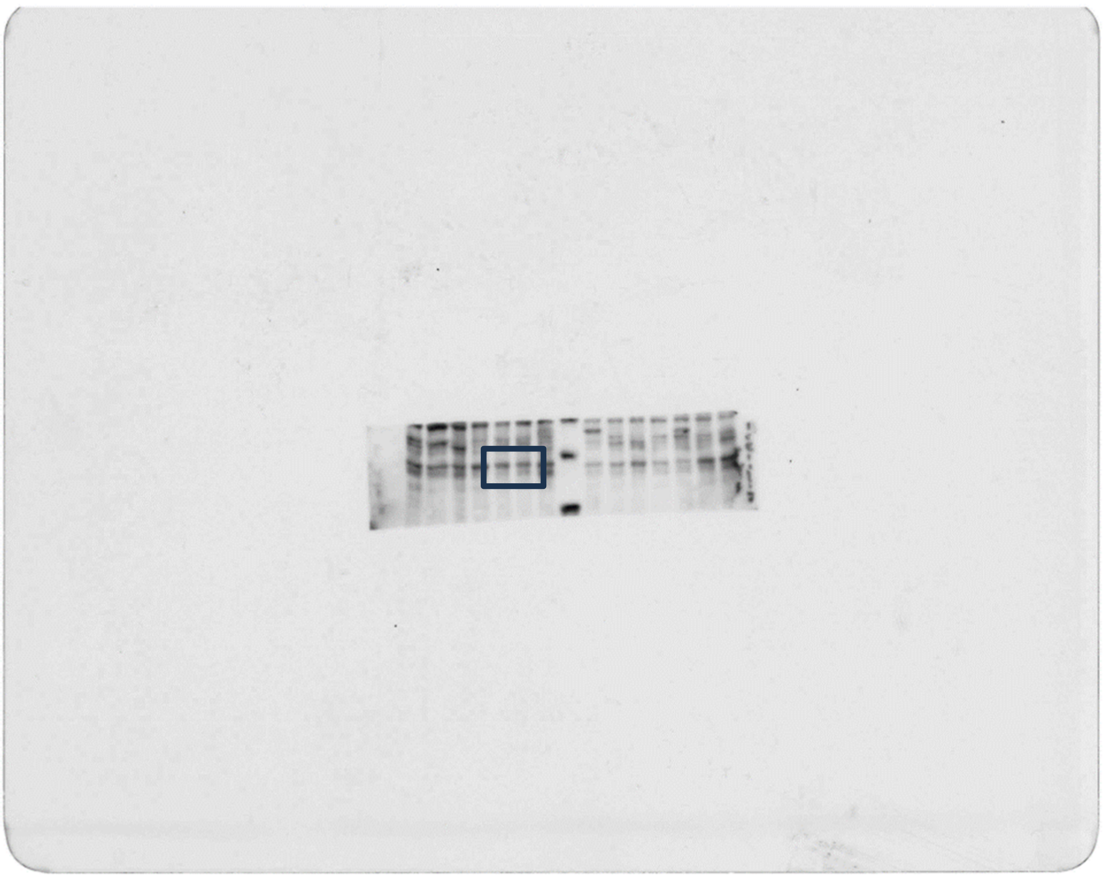

Fig7a
NDUFA9

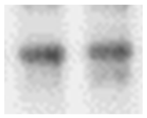

Supplementary Figure S19

Second set of uncropped blots of FP in Figure 7a

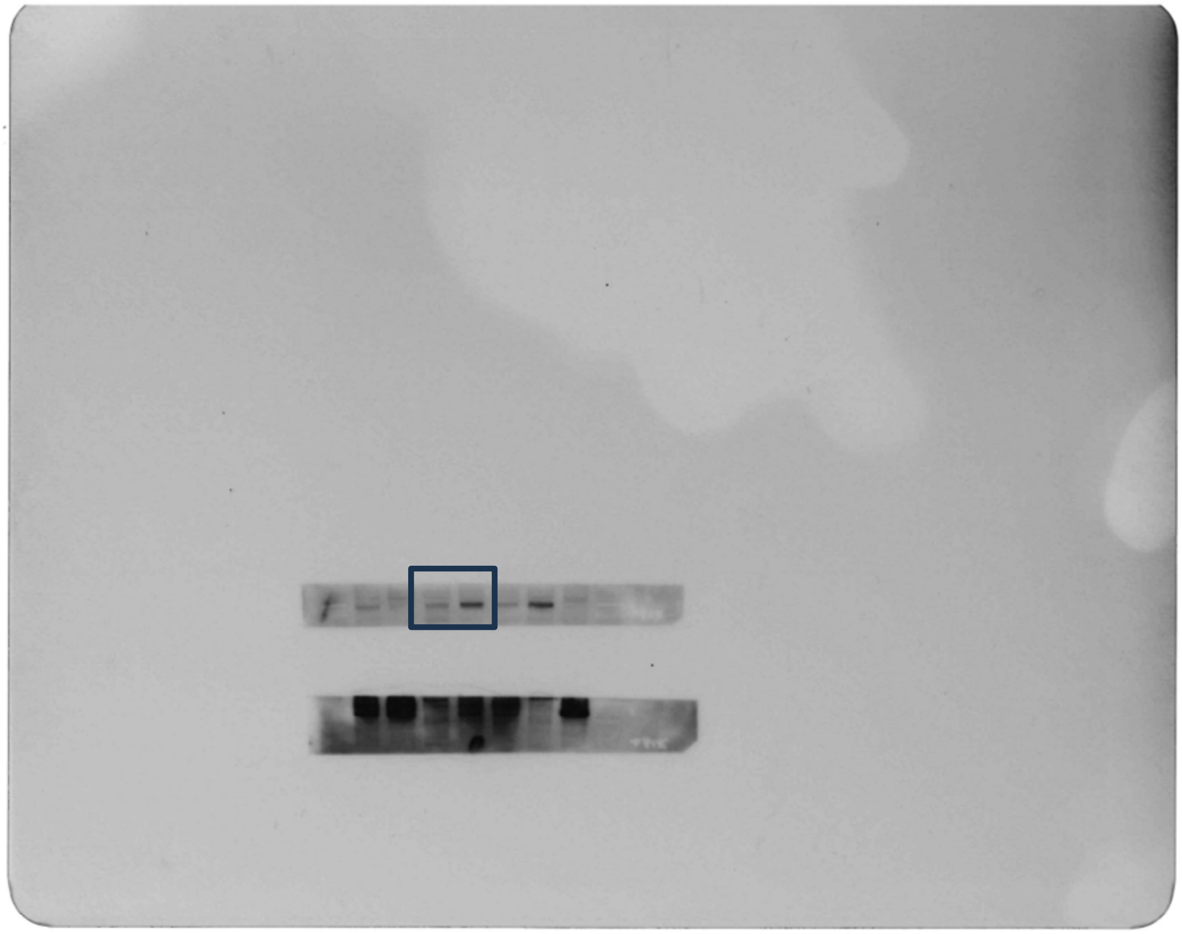

Fig7a
FP

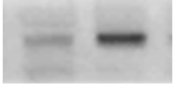

Cont CKO

Supplementary Figure S20

Second set of uncropped blots of
UQCRC2, ATP5A1, GAPDH in Figure 7a

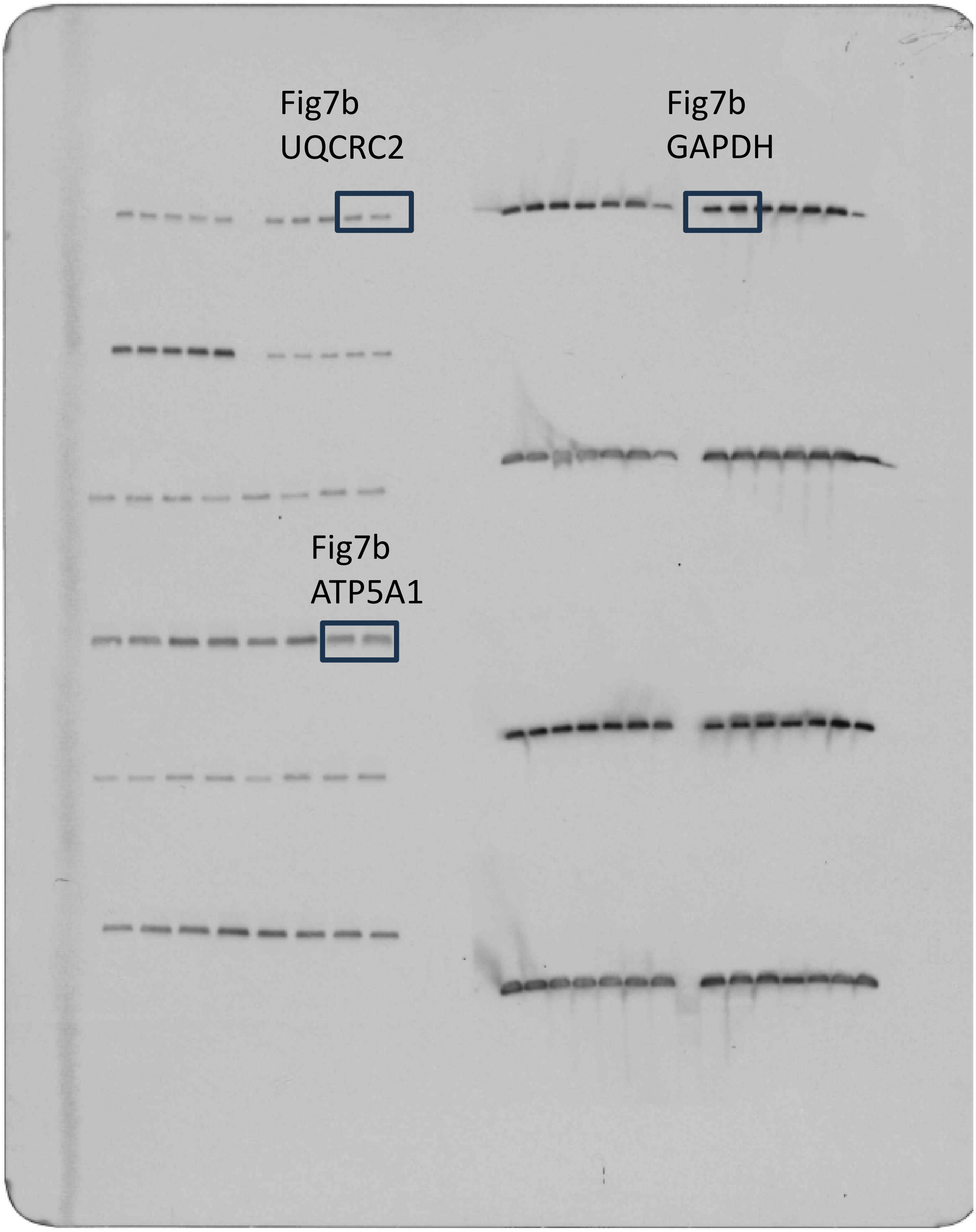

Fig7a

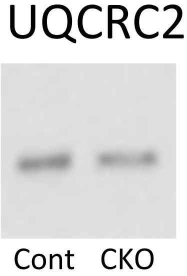

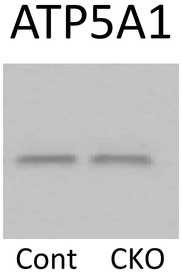

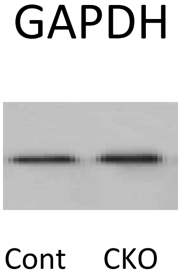

Supplementary Figure S21

Second set of uncropped blots of COX-1 in Figure 7a

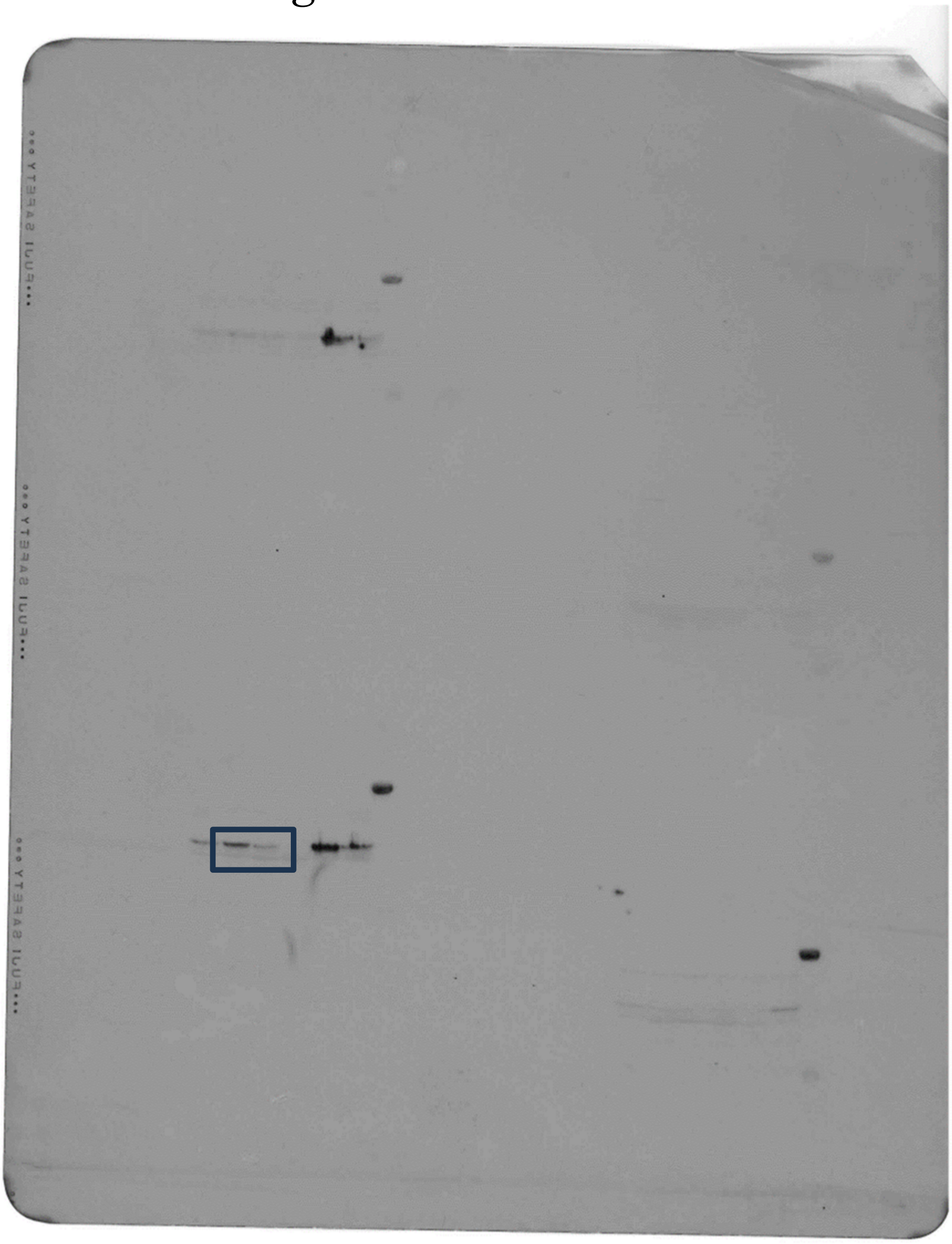

Fig7a
COX-1

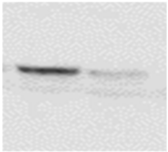

Cont CKO

Supplementary Figure S22

Second set of uncropped blots of ND1 in Figure 7a

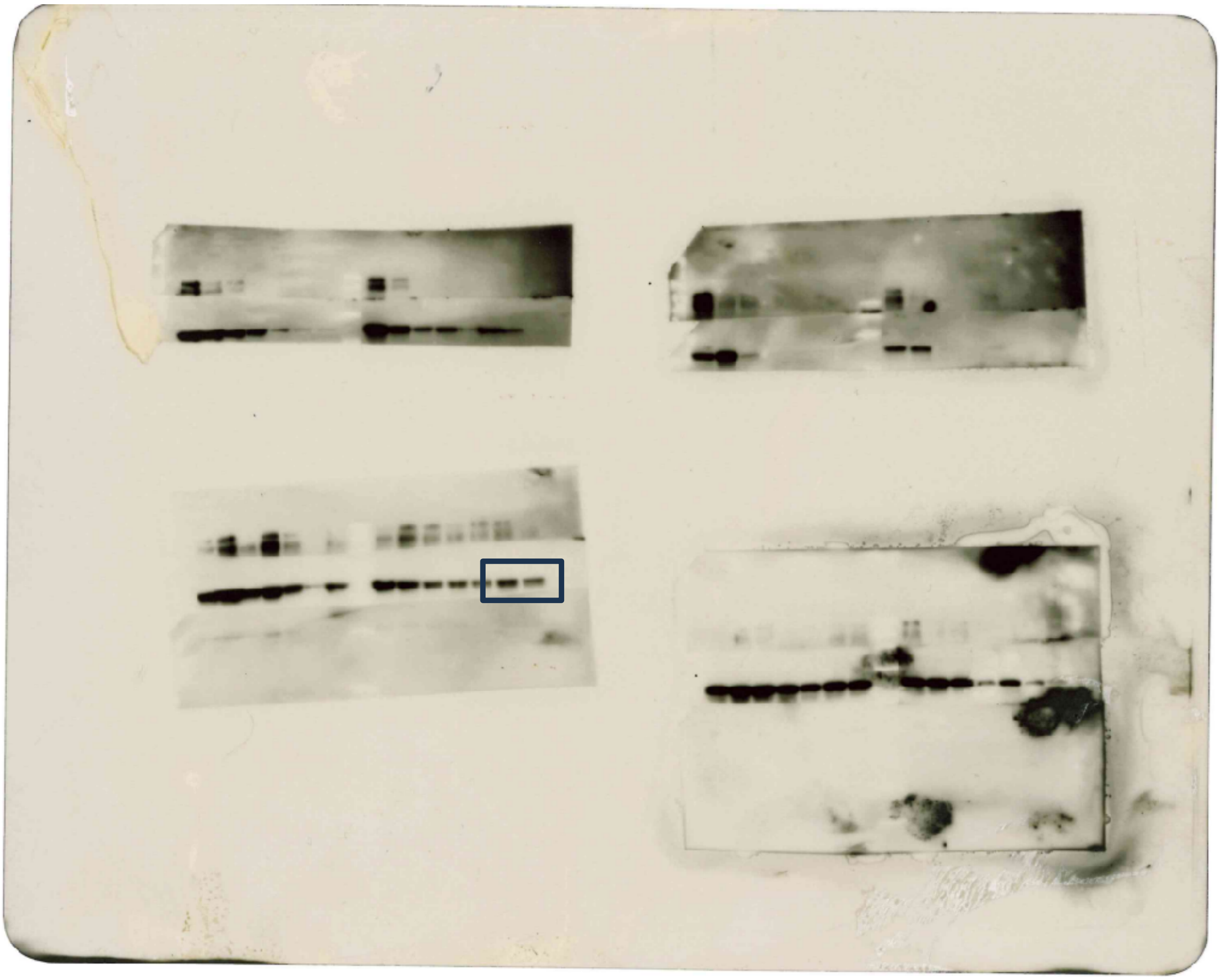

Fig7a
ND1

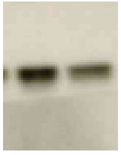

ContCKO

Supplementary Figure S23

a

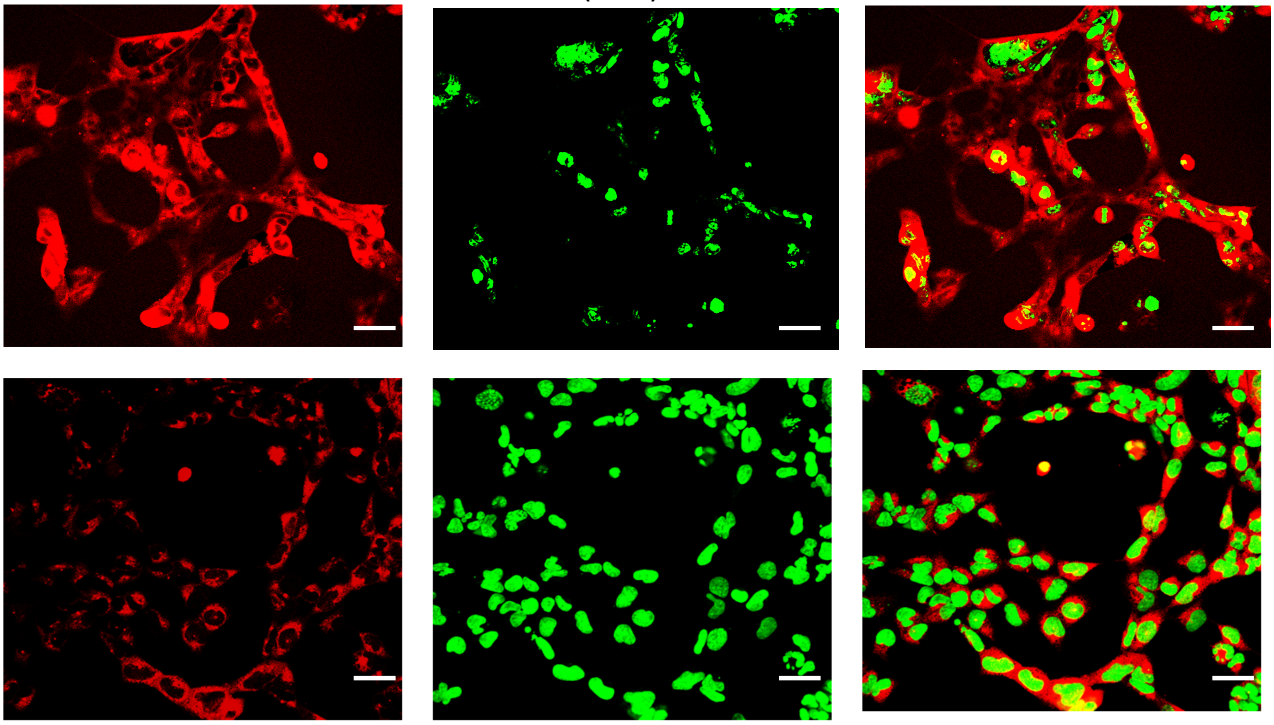

b

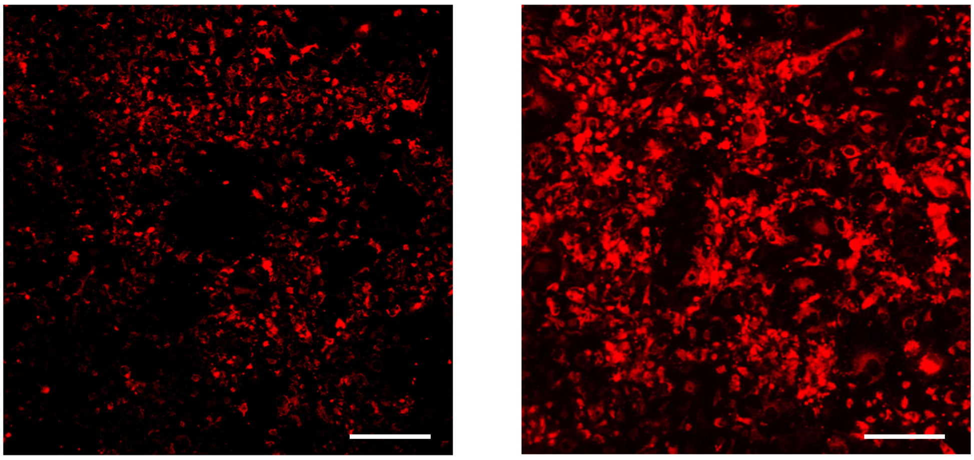

Supplementary Figure S24

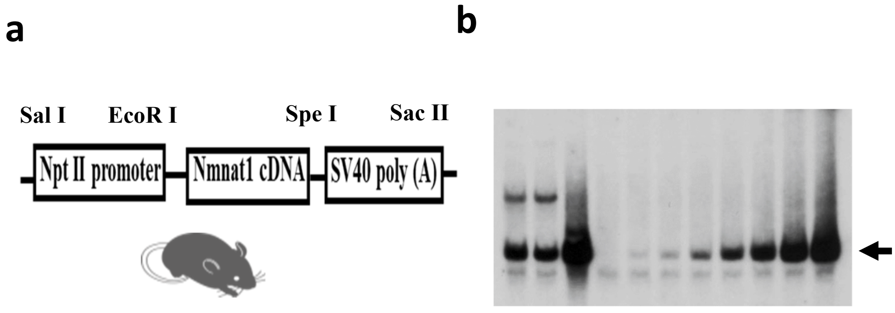

Lane No.	ID	Band intensity (WT=0)	Copy number
1	004 F1-003	101.43	18
2	006 F1-005	99.49	17
3	008 F1-007	192.53	112
WT	Wild Type Genome	0	0
x1	standard (1copy)	7.32	1
x2	(2copy)	12.71	2
x5	(5copy)	34.99	5
x10	(10copy)	72.08	10
x20	(20copy)	110.64	20
x50	(50copy)	152.34	50
x100	(100copy)	176.31	100

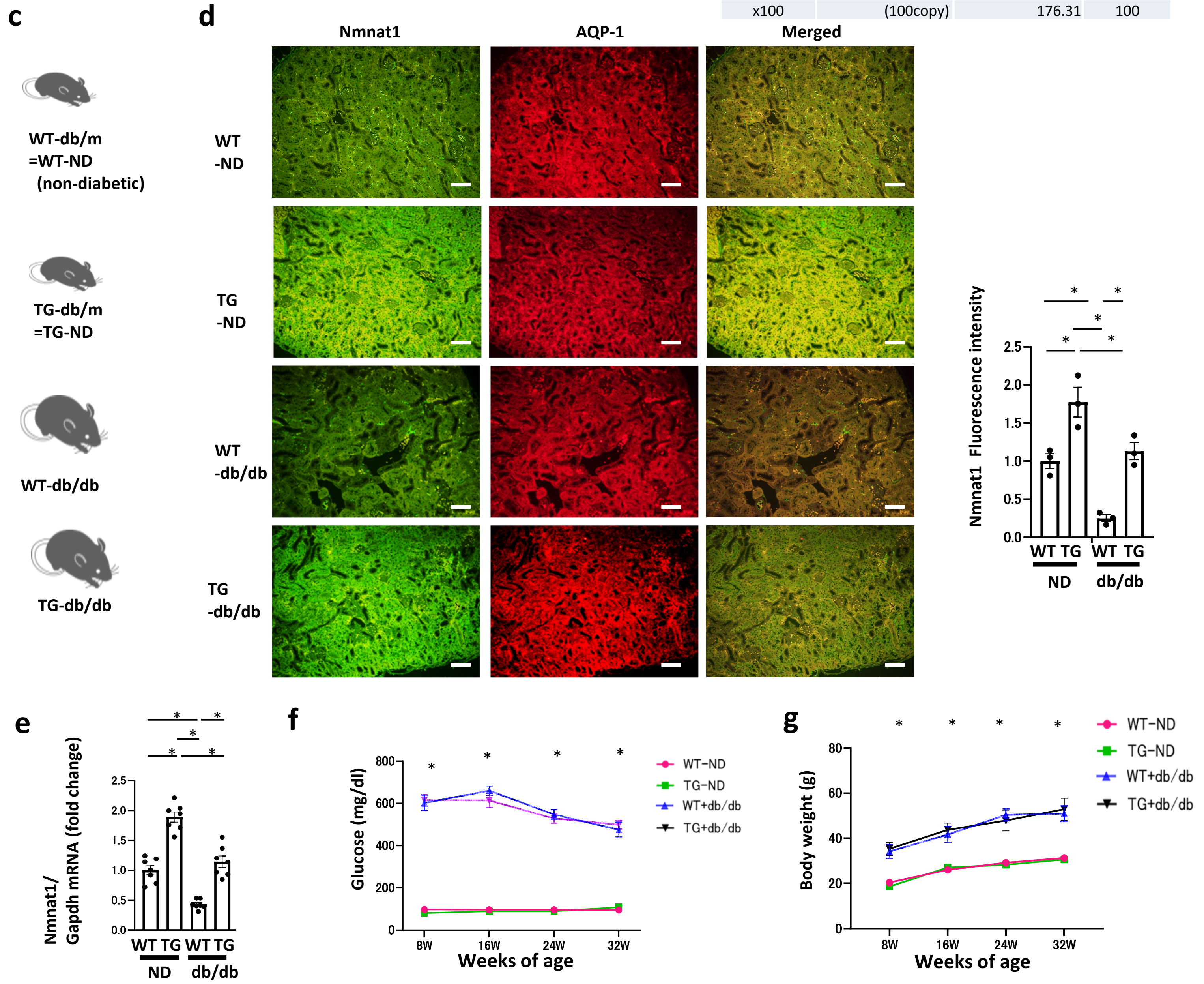

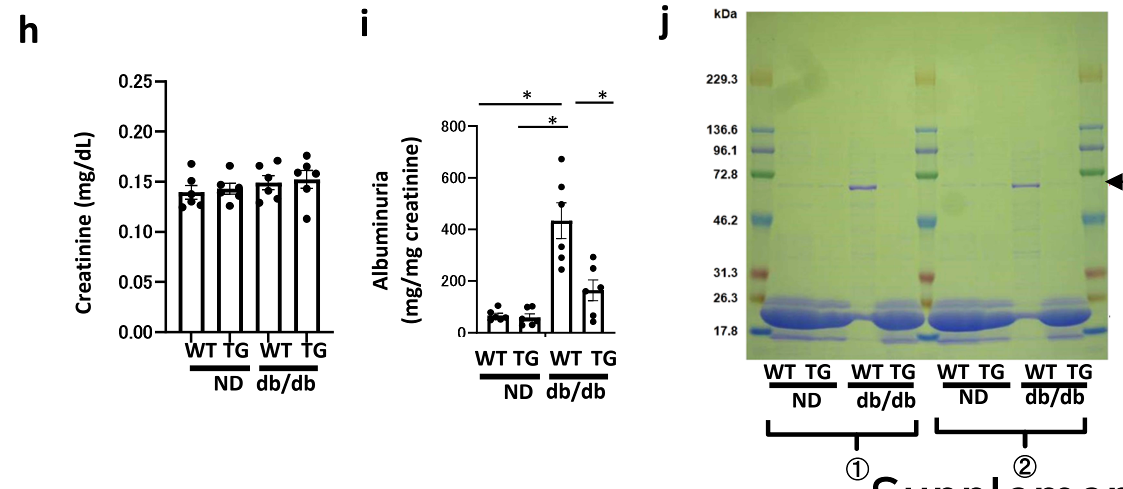

Lane No.	Sample name
1	Marker
2	WT 1
3	TG 1
4	WT+db/db 1
5	TG+db/db 1
6	Marker
7	WT 2
8	TG 2
9	WT+db/db 2
10	TG+db/db 2
11	Marker

Supplementary Figure S25

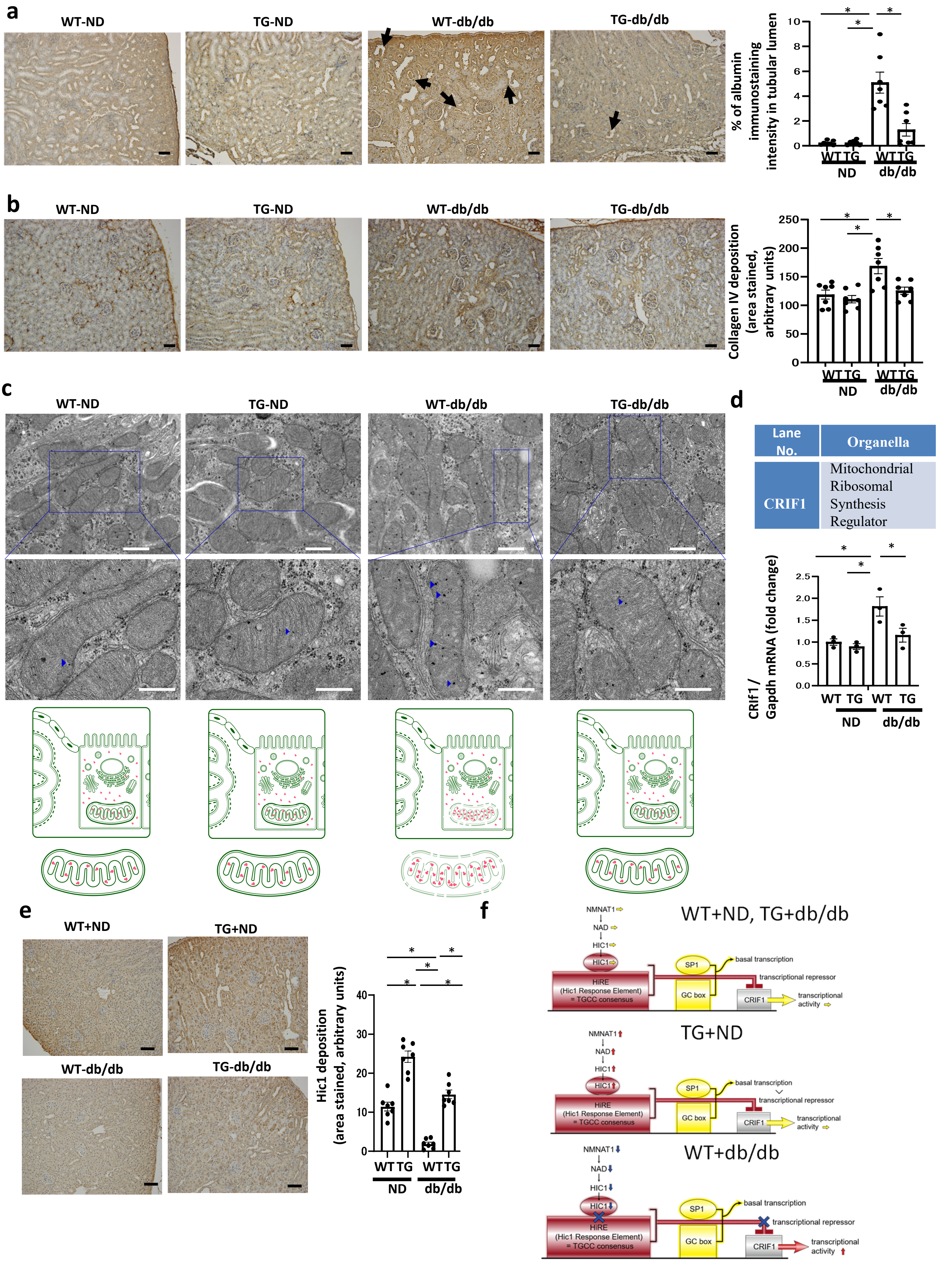

Supplementary Figure S26

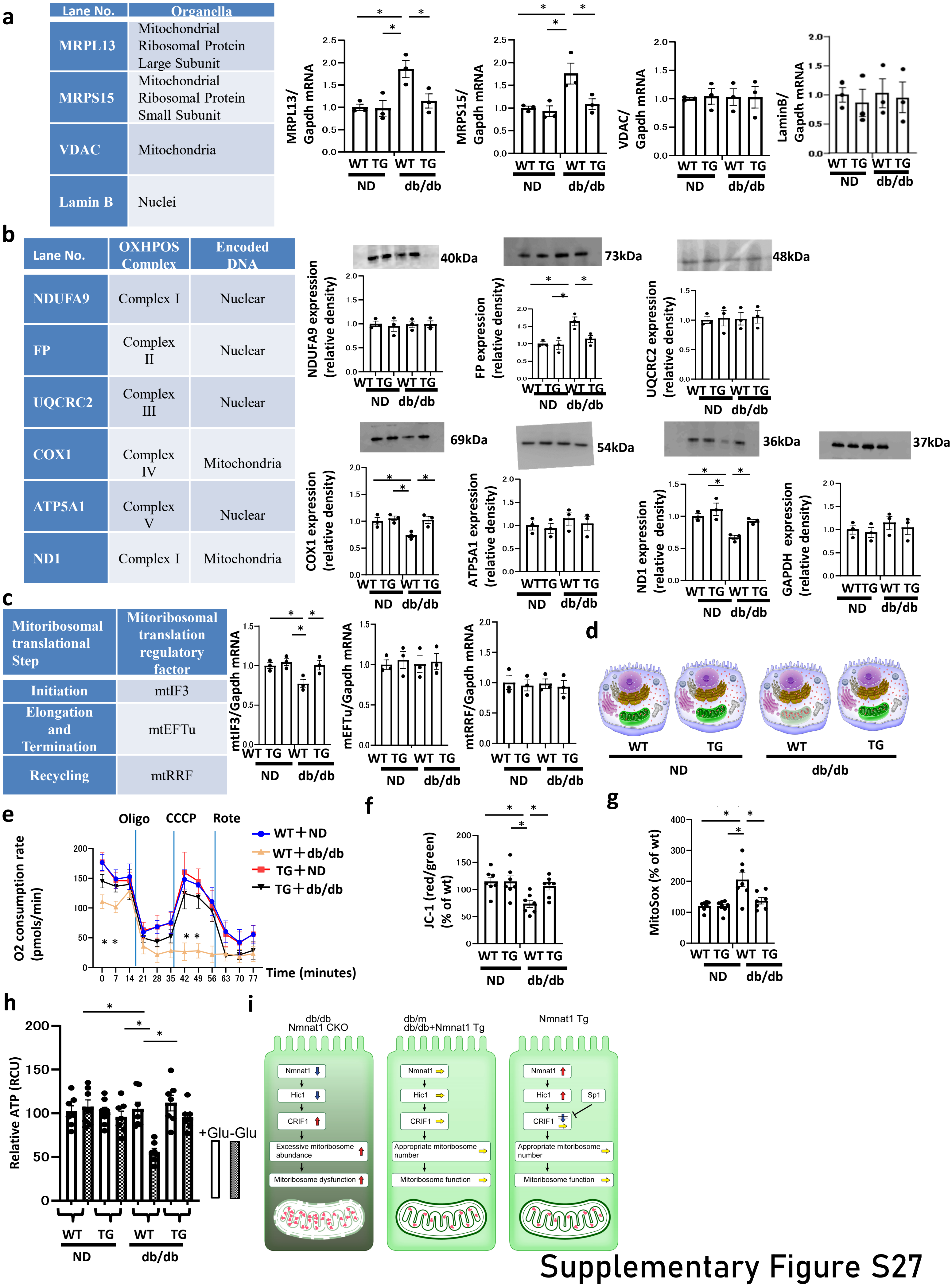

Supplementary Figure S27

Uncropped blots of NDUFA9
in Supplementary Figure 24b

NDUFA9

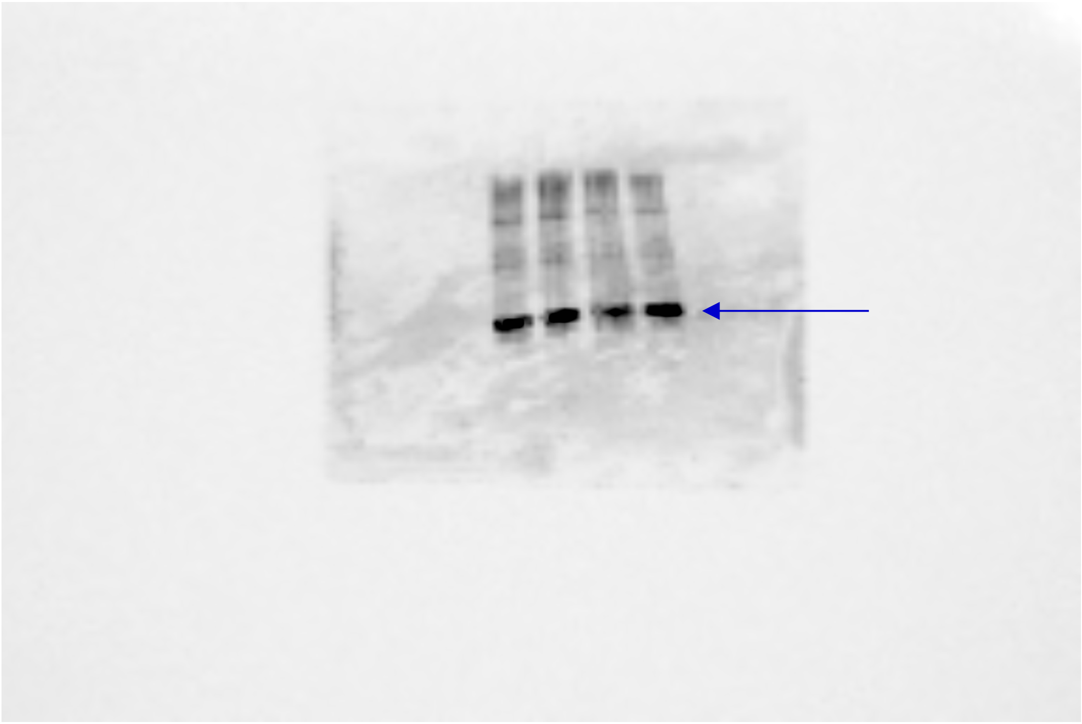

Uncropped blots of FP in Supplementary Figure 24b

FP

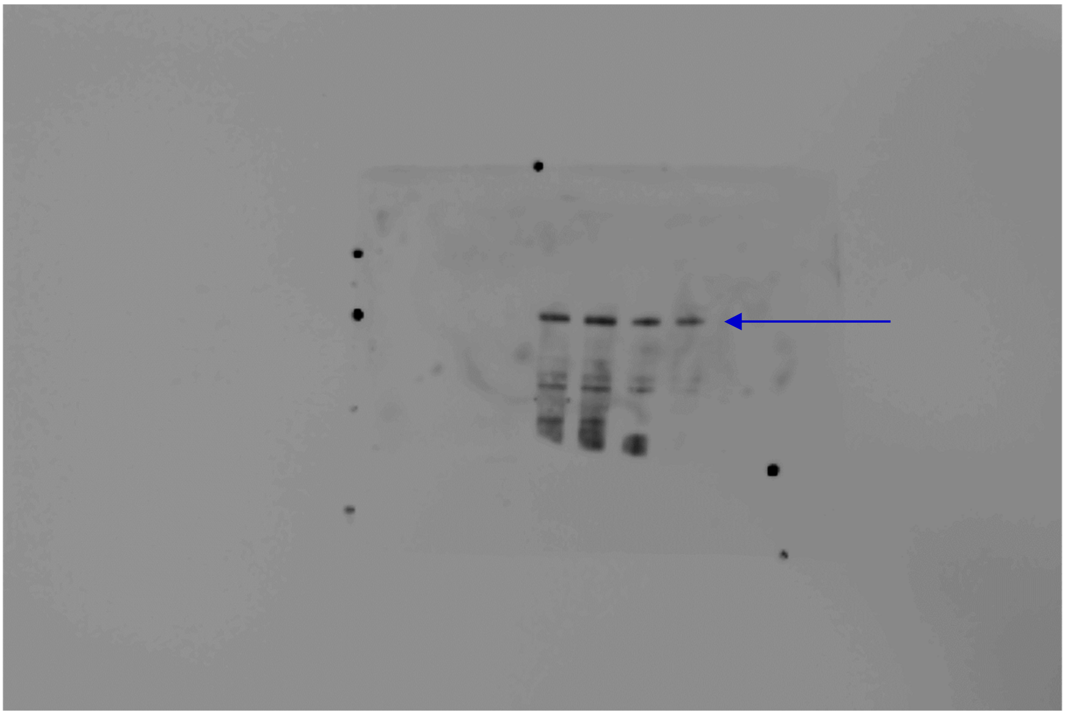

Supplementary Figure S29

Uncropped blots of UQCRC2 in Supplementary Figure 24b

UQCRC2

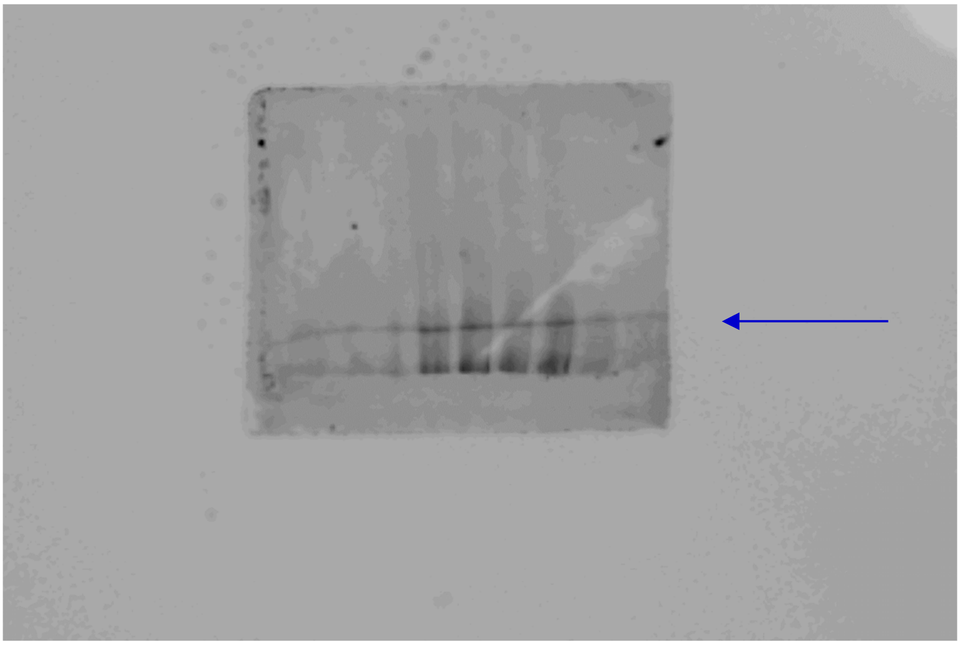

Uncropped blots of COX1 in Supplementary Figure 24b

COX1

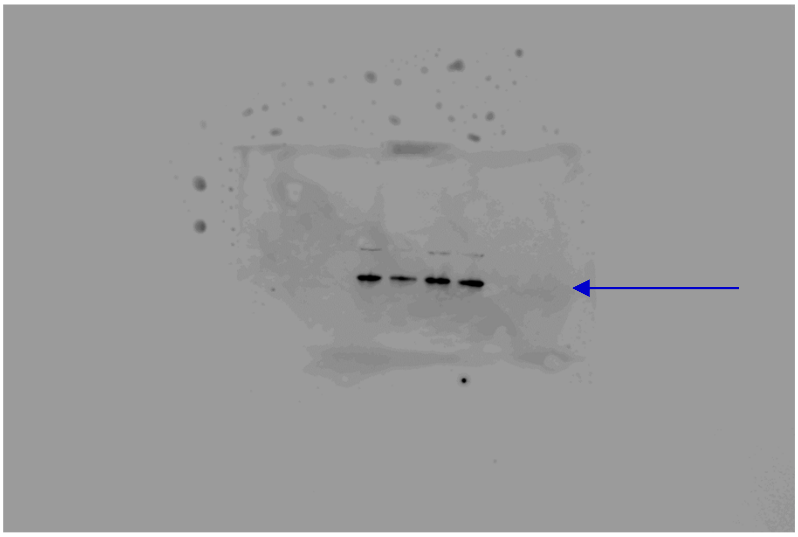

Uncropped blots of ATP5A1 in Supplementary Figure 24b

ATP5A1

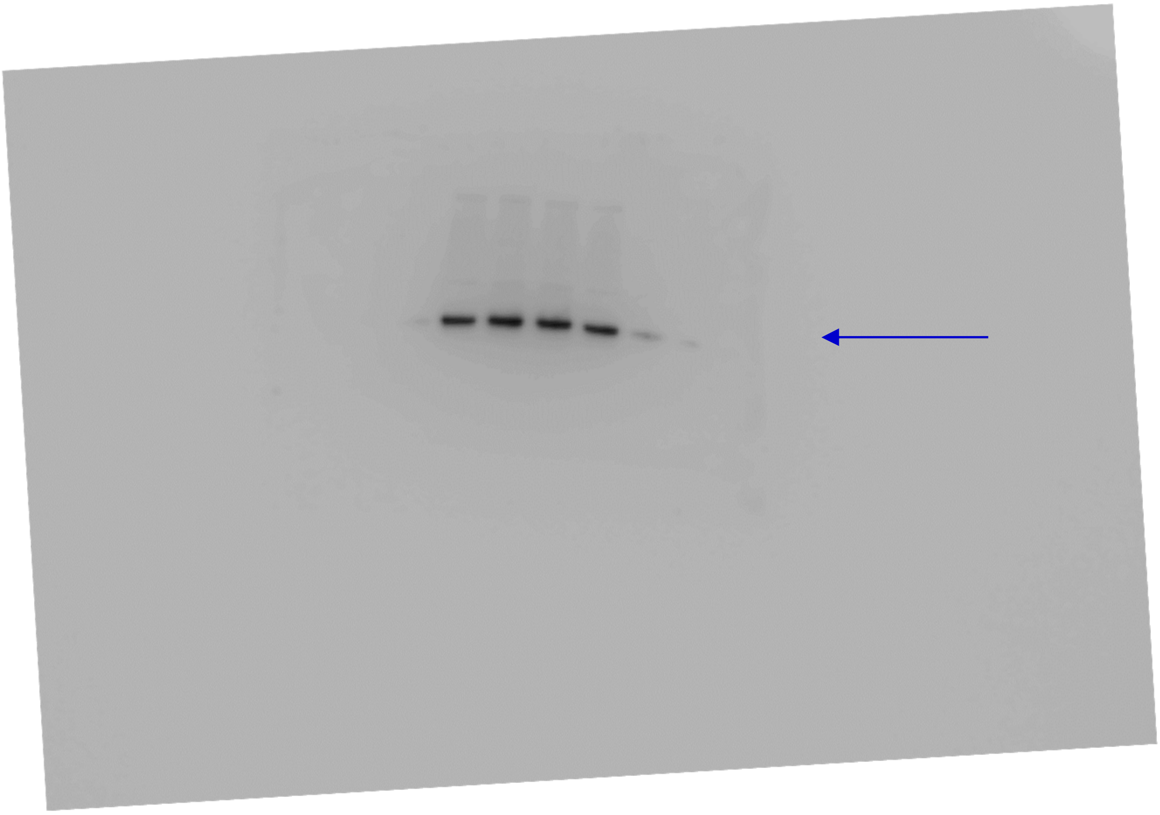

Supplementary Figure S32

Uncropped blots of ND1 in Supplementary Figure 24b

ND1

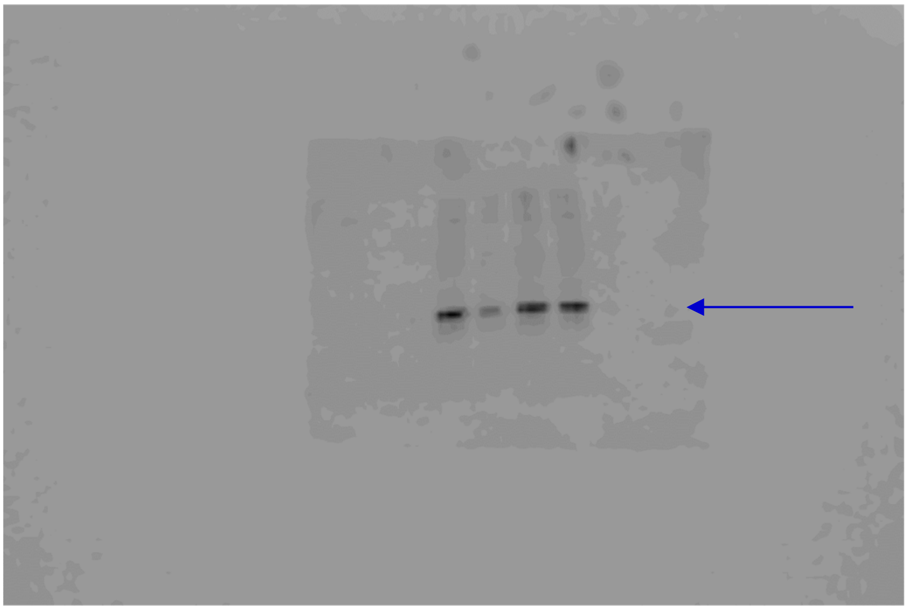

Uncropped blots of GAPDH in Supplementary Figure 24b

GAPDH

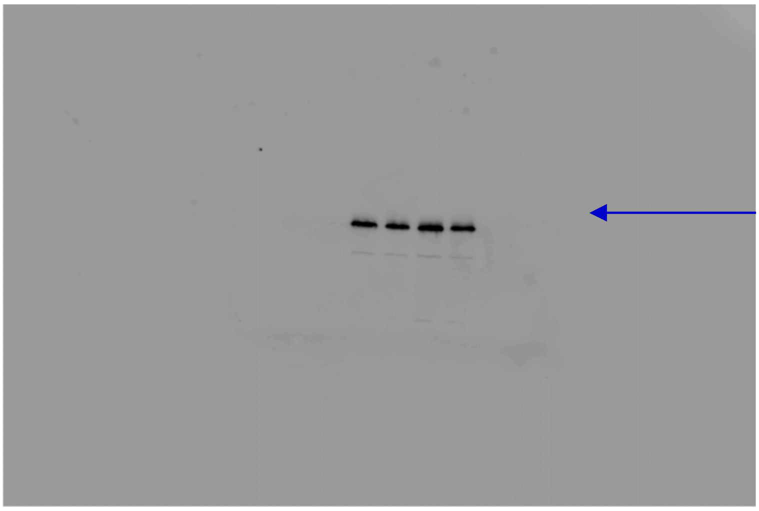

Supplementary Materials

Supplemental Figure S1

Nmnat 2 and 3 levels in db/db mice

Immunolocalization of Nmnat2 (a) and Nmnat3 (b) in the kidneys of 32-week-old nondiabetic control mice (db/m) and diabetic mice (db/db) mice. The protein expression in the control and diabetic kidneys was examined through immunohistochemistry; representative images are shown. Positively expressed proteins were stained brown by 3,3′-diaminobenzidine1 agent. Scale bar, 50 μm; N = 7. Results of immunohistochemical scoring assessing the whole kidneys are expressed as the mean ± SEM. *$p < 0.05$.

Supplementary Figure S2

Nmnat1 immunostaining and clinical parameters in human renal biopsy specimens. Relationships between eGFR, urinary proteinuria, serum creatine levels, and immunostaining for Nmnat1 in kidneys. $n = 11$ subjects. *P* values throughout the figure were determined by Pearson's *R* test.

Supplementary Figure S3

Western blot analysis of renal protein levels of Nmnat1.

(a) The kidney tissue specimens were obtained from CKO and control mice at 32 weeks of age. (b) The kidney tissue specimens were obtained from db/m and db/db mice at 32 weeks old. The results of one representative experiment of the three performed are shown. All data are indicated as the mean ± standard error of the mean. Horizontal bars denote statistically significant differences between groups. *$P < 0.05$. The bar graph in the lower panels illustrates the quantification of the band intensity. Glyceraldehyde 3-phosphate dehydrogenase was used as a control. N = 3 mice per group.

Supplementary Figure S4

Western blot analysis of renal protein levels of megalin, cubilin, and amnioless.

The kidney tissue specimens were obtained from CKO and control mice at 32 weeks of age. The results of one representative experiment of the three performed are shown. All data are indicated as the mean ± standard error of the mean. Horizontal bars denote statistically significant differences between groups. *$P < 0.05$. The bar graph in the lower panels illustrates the quantification of the band intensity. Glyceraldehyde 3-phosphate dehydrogenase was used as a control. N = 3 mice per group.

Supplementary Figure S5

Real-time quantitative reverse transcription analysis of the renal mRNA levels of TGF-β and type IV collagen.

For RT-PCR, the kidney tissue specimens were obtained from CKO and control mice at 32 weeks old. N = 3 mice per group.

Supplementary Figure S6

Western blot analysis of renal protein levels of fibronectin and α-SMA.

The kidney tissue specimens were obtained from CKO and control mice at 32 weeks of age. The results of one representative experiment of the three performed are shown. All data are indicated as the mean ± standard error of the mean. Horizontal bars denote statistically significant differences between groups. $*P < 0.05$. The bar graph in the lower panels illustrates the quantification of the band intensity. Glyceraldehyde 3-phosphate dehydrogenase was used as a control. N = 3 mice per group.

Supplementary Figure S7

Example of higher magnification images using TEM studies from Cont mice and CKO mice.

(a) TEM image showing mitochondria including mitoribosomes. (b) Mitoribosome magnification in (a) was highlighted as a blue box. (c) Higher mitoribosome magnification in (b) was highlighted as a blue box. Scale bar, 500 nm.

Supplementary Figure S8

Western blot analysis of renal protein levels of CRIF1.

The kidney tissue specimens were obtained from CKO and control mice at 32 weeks of age. The results of one representative experiment of the three performed are shown. All data are indicated as the mean ± standard error of the mean. Horizontal bars denote statistically significant differences between groups. $*P < 0.05$. The bar graph in the lower panels illustrates the quantification of the band intensity. Glyceraldehyde 3-phosphate dehydrogenase was used as a control. N = 3 mice per group.

Supplementary Figure S9

Hic1 regulates CRIF1 expression by activating its promoter.

Effects of Hic1 overexpression on CRIF1 promoter activity. Luciferase activity is shown relative to that of the −649 Luc vector in control cells. Values are expressed as means ± SE. $*P < 0.05$ vs. −649Luc in control cells; $^{\#}P < 0.05$ vs. −649Luc in Sirt1-transfected cells. (n = 3 independent experiments). Mutation in CRIF1 promoter activity was also analyzed. −649Luc, WT CRIF1 promoter between −649 and +1; –649 (HiRE m) Luc, mutation in Hic1-binding site.

Supplementary Figure S10

Knockdown effects of Nmnat1 on Hic1 and CRIF1 expression in HK2 cells.

HK2 cells were transiently transfected with siRNAs targeting Nmnat1 control siRNA. Whole cell lysates were prepared 48 h after transfection and analyzed using immunoblotting. Results represent 3 independent experiments including Nmnat1 (top panel), HIC1 (2nd from the top panel), CRIF1 proteins

(3rd from the top panel), and GAPDH (bottom panel). The bar graph denotes the band intensity of each group (*$P < 0.05$ vs. without siRNAs, §$P < 0.05$ vs. Cont siRNA).

Supplementary Figure S11

Real-time quantitative reverse transcription analysis of the renal mRNA levels of Hic1.

For RT-PCR, the kidney tissue specimens were obtained from CKO and control mice at 32 weeks old. N = 3 mice per group.

Supplementary Figures S12–18

First set of uncropped blots of Figure 7a

Supplementary Figures S19–23

Second set of uncropped blots of Figure 7a

Supplementary Figure S24

Fluorescent images of JC-1 and MitoSOX

(a) Representative images of JC-1 staining showing red fluorescence of JC-1 aggregate and green signal of monomer. Scale bar: 50 μm (400×). (b) Representative images of MitoSOX staining (red). Scale bar: 50 μm (400×).

Supplementary Figure S25

Generation and antialbuminuric phenotypes of TG mice

(a) Constructs used for generating TG mice. A fragment is composed of the Npt2 promoter, murine *Nmnat1* cDNA, and SV40 poly(A) sequences. (b) Southern blotting shows copies of the *Nmnat1* TG in mice. Arrows indicate bands indicating TG-derived *Nmnat1*. (c) Schematic showing the 4 mouse groups used in the following research. (d) Representative immunofluorescence images from kidney cryosections derived from each experimental group: WT-ND (db/m), WT-db/db, TG-ND, and TG-db/db mice at 32 weeks old. These sections were stained by immunofluorescence for *Nmnat1* (green) and AQP1 (red). N = 3. (e) The real-time PCR reveals *Nmnat1* existence in all four mouse groups. N = 3. (f) Temporal changes in mean plasma glucose concentrations in mice from each group. We examined the mice at 8, 16, 24, and 32 weeks old. N = 7 mice per group. (g) Body weight changes in mice from each group at 8, 16, 24, and 32 weeks old. N = 7 mice per group. (h) Serum creatine levels in each mouse group at 32 weeks old. N = 6. (i) Urinary albumin excretion in the 4 mouse groups at 32 weeks old. N = 6. (J) SDS-PAGE of mouse urine samples. Urine samples of the 4 mouse groups at 32 weeks old were subjected to a 15% SDS-PAGE before Coomassie blue staining. N = 2 mice per group. TG, transgene; WT, wild type,

Supplementary Figure S26

Antialbuminuric and mitoribosomal protective phenotypes of TG mice

(a) Representative pictures of albumin staining. Arrows indicate albumin casts. The right panel depicts the relative staining intensities. N = 7 mice per group. (b) Representative photomicrographs showing collagen IV immunostaining in each group. The bar graph illustrates the quantitative analysis of collagen IV staining. N = 7 per mice group. (c) Representative electron micrograph in each group. Scale bar = 500 nm. Blue squares indicate the enlarged regions. Illustration depicts the mitoribosomes in the four mouse groups. Expanded images are also presented. Blue arrowheads indicate mitoribosomes. Scale bar = 500 nm. For EM, the kidney tissue specimens were embedded in Epon epoxy resin. Electron micrographs of 10 proximal tubules (PTs) per kidney were randomly obtained for each mouse to evaluate PT morphometry. (d) Real-time quantitative reverse transcription analysis of the renal mRNA levels of CRIF1. GAPDH was used as a control. The kidney tissue specimens for RT-PCR were obtained from 4 mouse groups at 32 weeks old. N = 3 mice per group. (e) Representative photomicrographs showing Hic1 immunostaining in each group. The bar graph illustrates the quantitative analysis. N = 7 per mice group. (a, b, and e) Light micrograph; scale bar = 100 μm. Kidney tissue specimens for immunostaining were obtained from the four mouse groups at 32 weeks old. All data are shown as mean ± standard error of the mean. Horizontal bars indicate statistically significant differences in each group. $*p < 0.05$. (f) Schematic representation of the murine *CRIF1* gene and promoter. The solid boxes indicate HiRE binding to Hic1 and GC box binding to Sp1, highlighted in red and yellow, respectively. GAPDH, glyceraldehyde 3-phosphate dehydrogenase; WT, wild type; TG, transgene; CRIF1, CR6-interacting factor 1; Hic1, hypermethylated in cancer 1; Nmnat, nicotinamide mononucleotide adenylyl transferase; NAD, nicotinamide adenine dinucleotide; HiRE, Hic-responsive element; Sp1, specificity protein 1

Supplementary Figure S27

Mitoribosome protection and mitochondrial protection in TG mice

(a) The real-time quantitative reverse transcription analysis of the renal mRNA levels of intracellular organelle markers. MRPL13 and MRPS15 are mitoribosomal proteins, VDAC is a mitochondrial protein, and Lamin B is a nuclear protein. (b) Western blot analysis of renal protein levels of OXPHOS subunits encoded by nDNA and mtDNA. The kidney tissue specimens were obtained from each group of mice at 32 weeks of age. The results of one representative experiment of the three performed are shown. All data are indicated as the mean ± standard error of the mean. Horizontal bars denote statistically significant differences between groups. $*P < 0.05$. The bar graph in the lower panels illustrates the quantification of the band intensity. Glyceraldehyde 3-phosphate dehydrogenase was used as a control. N = 3 mice per group. (c) Real-time quantitative reverse transcription analysis of the renal mRNA levels of mitoribosomal translational regulators. (a-c) Glyceraldehyde 3-phosphate dehydrogenase was used as a control. The kidneys for RT-PCR were obtained from each mouse group

at 32 weeks old. N = 3 mice per group. (d) Illustration depicting the dysfunctional mitoribosomes and their concomitant mitochondrial dysfunction in WT-db/db mice. These changes in WT-db/db mice were resisted by TG-db/db mice. (e) The OCR of tubular epithelial cells (TECs) isolated from each group of mice was measured using a Seahorse XF-24 flux analyzer. N = 3. (f) The ratio of red/green fluorescence of JC-1 of TECs isolated from each mouse group as a measure of the mitochondrial membrane potential. N = 6. (g) Fluorescence of MitoSox of TECs isolated from each mouse group as a measure of mitochondrial levels of reactive oxygen species. N = 7. (h) The ATP content of TECs isolated from each mouse group. N = 7. All data are presented as mean ± standard errors of the mean. Horizontal bars indicate statistically significant differences between groups. $*p < 0.05$. (i) Scheme depicting the new mitoribosome-mediated mechanism of renal profibrotic changes and tubular mitoribosomal dysfunction and mitochondrial dysfunction in diabetic nephropathy. Under db/db-induced diabetic conditions, *Nmnat1* downregulation decreased Hic1 expression, leading to elevated CRIF1 expression and eventually, mitoribosome excess. Excessive mitoribosomes lead to the mitoribosomal dysfunction accompanied by collagen IV deposition and OXPHOS impairment. TG inhibited these changes. (j) The left scheme shows the highly dense mitoribosomal area, which downregulated mitoribosomal translation in CKO mice and db/db mice. The middle and right schemes depict the appropriate number of mitoribosomes retaining mitoribosomal translation. Horizontal bars represent statistically significant differences in each group. $*p < 0.05$. MRPL13, mitochondrial ribosomal protein L13; MRPS15, mitochondrial ribosomal protein S15; VDAC, voltage dependent anion channel; GAPDH, glyceraldehyde 3-phosphate dehydrogenase; WT, wild type; TG, transgene; ATP8, adenosine triphosphate 8; ATP5A1, ATP synthase F1 subunit alpha; UQCRC2, ubiquinol-cytochrome c reductase core protein 2; cyto b, cytochrome b; mtRRF; mitochondrial ribosome recycling factor; mtIF3, mitochondrial translational initiation factor 3; mtEFTu, mitochondrial elongation factor EFTu; NDUFA9, NADH:ubiquinone oxidoreductase subunit A9; FP, fluorescent protein; CCCP, carbonyl cyanide m-chlorophenyl hydrazone

Supplementary Figure S28-34

Uncropped blots of Supplementary Figure S24b
